# Pyruvate Oxidase Influences the Sugar Utilization Pattern and Capsule Production in *Streptococcus pneumoniae*

**DOI:** 10.1371/journal.pone.0068277

**Published:** 2013-07-03

**Authors:** Sandra M. Carvalho, Vahid Farshchi Andisi, Henrik Gradstedt, Jolanda Neef, Oscar P. Kuipers, Ana R. Neves, Jetta J. E. Bijlsma

**Affiliations:** 1 Instituto de Tecnologia Química e Biológica, Universidade Nova de Lisboa, Oeiras, Portugal; 2 Department of Medical Microbiology, University of Groningen, University Medical Center Groningen, Groningen, The Netherlands; 3 Department of Molecular Genetics, Groningen Biomolecular Sciences and Biotechnology Institute, University of Groningen, Groningen, The Netherlands; Duke University, United States of America

## Abstract

Pyruvate oxidase is a key function in the metabolism and lifestyle of many lactic acid bacteria and its activity depends on the presence of environmental oxygen. In *Streptococcus pneumoniae* the protein has been suggested to play a major role in metabolism and has been implicated in virulence, oxidative stress survival and death in stationary phase. Under semi-aerobic conditions, transcriptomic and metabolite profiling analysis of a *spxB* mutant grown on glucose showed minor changes compared to the wild type, apart from the significant induction of two operons involved in carbohydrate uptake and processing. This induction leads to a change in the sugar utilization capabilities of the bacterium, as indicated by the analysis of the growth profiles of the D39 parent and *spxB* mutant on alternative carbohydrates. Metabolic analysis and growth experiments showed that inactivation of SpxB has no effect on the glucose fermentation pattern, except under aerobic conditions. More importantly, we show that mutation of *spxB* results in the production of increased amounts of capsule, the major virulence factor of *S. pneumoniae*. Part of this increase can be attributed to induction of capsule operon (*cps*) transcription. Therefore, we propose that *S. pneumoniae* utilizes pyruvate oxidase as an indirect sensor of the oxygenation of the environment, resulting in the adaption of its nutritional capability and the amount of capsule to survive in the host.

## Introduction

*Streptococcus pneumoniae* is an important human pathogen as it is a common cause of respiratory pathologies and serious invasive diseases such as pneumonia, sepsis and meningitis; especially infants, the elderly and immuno-compromised individuals are at risk. Annually, over 1 million children die of pneumonia and meningitis and in the US alone 40.000 deaths each year are caused by pneumococcal pneumonia or meningitis [1]. Capsule, an extracellular structure of polysaccharide nature attached to the cell wall and membrane, is one of the main virulence factors of *S. pneumoniae*; its main purpose is to inhibit complement-mediated opsono-phagocytosis and acapsular mutants are avirulent [2–5]. The composition of the capsule varies from strain to strain and up to 93 different capsular serotypes have been identified [6]. Capsular polysaccharide synthesis is intimately connected with central carbon metabolism as it requires activated carbohydrates; a number of these NDP-sugars derive from the glycolytic intermediates glucose 6-phosphate and fructose 6-phosphate [7–9]. A direct correlation between capsule thickness and virulence has been reported [4,10]. The amount of capsule varies throughout infection and decreases dramatically in the proximity of, and while invading, eukaryotic cells [11,12]. In addition, *S. pneumoniae* displays at least two phenotypic variants, transparent and opaque, that are to a large extent determined by the quantity of capsule and have differences in virulence properties [13,14]. Transparent pneumococci are more efficient colonizers of the nasopharynx, produce less capsule, but more cell-wall associated teichoic acids than opaque variants [13–15]. Additionally, the two variants show differential production of various proteins, one of which is the pneumococcal pyruvate oxidase enzyme SpxB [16]. However, the factors determining the occurrence of phase variation and the regulation of the amount of capsule are still not entirely clear, nor the connections that exist between central carbon metabolism and capsule production.

SpxB is reported to play a central role in carbohydrate metabolism under semi-aerobic (microaerobic) conditions and in particular in the generation of the phosphoryl donor metabolite acetyl-phosphate (Ac–P) [17]. The enzyme decarboxylates pyruvate into Ac–P by reducing oxygen (O_2_) to hydrogen peroxide (H_2_O_2_) and consuming inorganic phosphate (P_i_). SpxB activity in *S. pneumoniae* might produce H_2_O_2_ in the mM range [17,18], concentrations higher than those generated by many other species [18,19], and sufficient to kill or inhibit other nasopharyngeal flora members, such as *Haemophilus influenzae* and *Neisseria* *meningitidis* [20]. Furthermore, these amounts have cytotoxic effects on human cells [21–23]. The *spxB* gene has also been designated as a suicide gene since the H_2_O_2_ induces pneumococcal death and reduces survival in stationary phase under semi-aerobic (microaerobic) conditions [24]. In addition, the H_2_O_2_ produced endogenously influences the membrane composition through modulation of fatty-acid biosynthesis F (FabF) protein activity [25,26]. Interestingly, and despite the potential deleterious effect of its product, SpxB has also been associated with streptococcal resistance to H_2_O_2_ [19]. Besides its function in central metabolism, a prominent role of SpxB in the virulence of the bacterium, especially in colonization and pneumonia, is corroborated by a number of studies [17,24,27,28]. Here, we studied the role of SpxB under semi-aerobic conditions as this is likely to reflect the atmospheric conditions encountered by *S. pneumoniae* in various host niches during infection [29]. We describe that the inactivation of *spxB* results in the overproduction of capsule under semi-aerobic conditions, which is partially mediated by increased transcription of the capsule *locus*. Furthermore, we characterized the transcriptomic and metabolic consequences of *spxB* inactivation, which consist of a change in sugar utilization capacities and hypothesize that these effects also contribute to its role in virulence.

## Results

### Inactivation of *spxB* leads to an increase in capsule production

**Figure 1:**
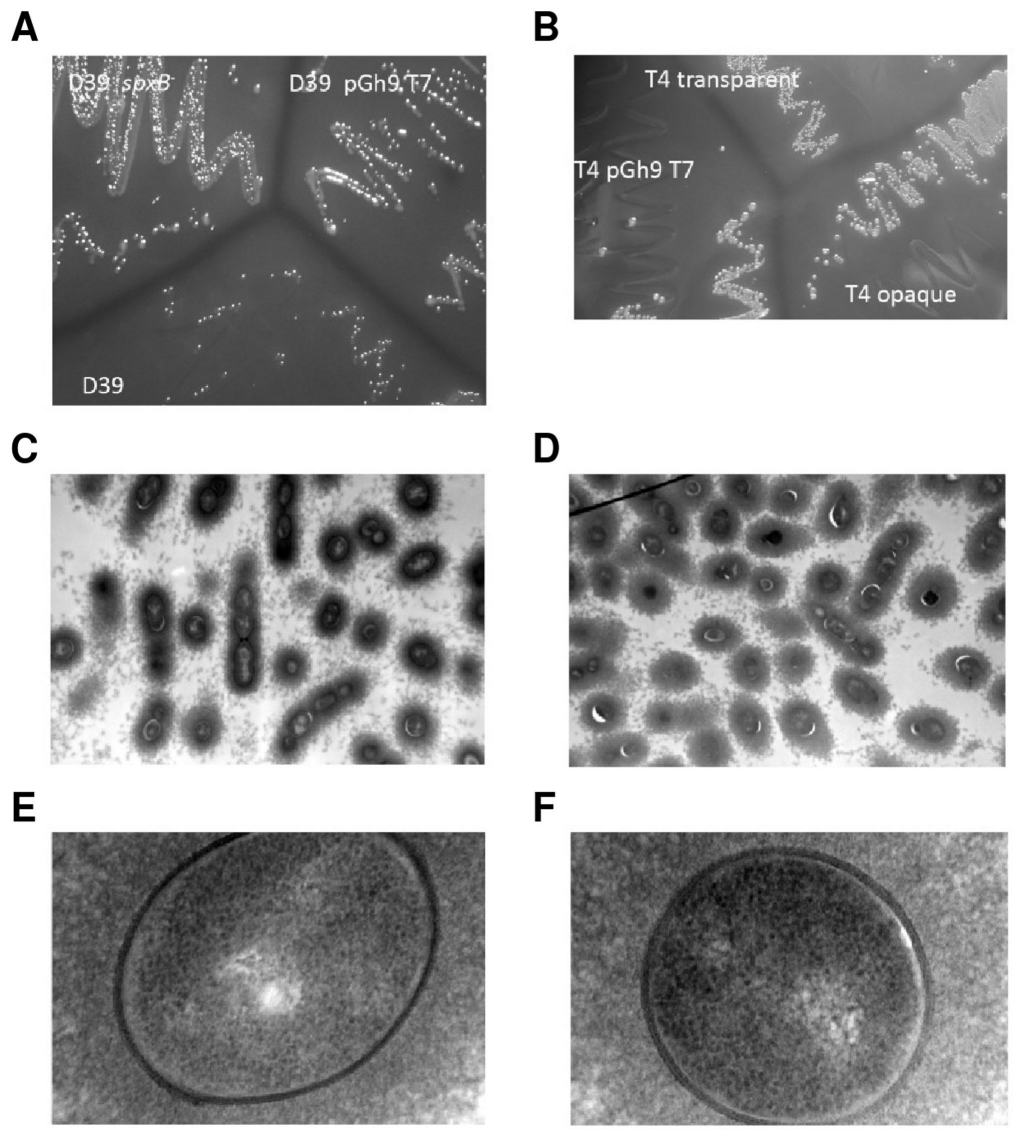
Inactivation of *spxB* led to an increase in capsule production. (A) Phenotype on Glc-M17 agar plate of D39 (D39), D39 containing the pGh9 T7 plasmid (D39pGh9 T7) and D39 in which the plasmid is excised leaving the ISS1 element in *spxB* (D39*spxB*). (B) Phenotype on Glc-M17 agar plate of TIGR4 transparent variant (T4 transparent), TIGR4 opaque variant (T4 opaque) and TIGR4 in which the pGh9 T7 plasmid is inserted into the genome (T4 pGh9 T7). (C–F) TEM pictures of *S. pneumoniae* grown in broth to exponential phase and stained with LRR. (C) D39, 9700 times magnified; (D) D39*spxB* 9700 times magnified; (E) D39, 135.000 times magnified; (F) D39*spxB* 135.000 times magnified.

While generating a transposon library in strain D39 using the pGh9:ISS1 T7 plasmid [30], a colony displaying characteristics of overproducing capsule was isolated; on solid medium this isolate yielded large, mucoidal and slimy colonies (Figure 1A). To ensure that the observed phenotype was genetically linked to the transposon insertion, the chromosomal DNA of this mutant was back-crossed into strain D39 which gave rise to the same phenotype. Transformation into strain TIGR4 also resulted in the same phenotype, indicating that the phenotype was serotype independent (Figure 1B). Comparison of the TIGR4 strain containing the insertion with an opaque and transparent variant of the same strain indicated that the phenotype of the mutant was dissimilar to the opaque phase variant as it seemed to produce even more capsule (Figure 1B). Introduction of the chromosomal DNA into an unencapsulated mutant of D39 or strain R6 [31,32], a spontaneous derivative of D39 displaying higher transformability, increased pyruvate oxidase activity and no capsule, did not result in the mucoidal phenotype (data not shown), which indicates that the observed phenotype is exclusively capsule-dependent. Excision of the pGh9:ISS1 T7 plasmid in two independent colonies of D39 resulted in the same phenotype as the original mutant, as did excision of the plasmid in a D39 strain that was backcrossed with the chromosomal DNA of the original mutant, excluding the possibility that the phenotype was due to a point mutation in the original mutant. Determination of the insertion site showed that the ISS1 element was inserted into the 3’ end of the *spxB* gene.

The *spxB* gene encodes the *S. pneumoniae* pyruvate oxidase, which generates Ac–P and H_2_O_2_ and whose activity plays a key role in pneumococcal colonization and pathogenesis [17,24,27,28]. The amount of H_2_O_2_ produced by the mutant was reduced at least 80% compared to the wild type and PCR analysis showed the insertion of approximately 600-bp extra into the *spxB* gene in all excised mutants (data not shown) and no SpxB protein could be detected (Figure S1), confirming that the insertion of ISS1 disrupted *spxB* and its function.

**Figure 2:**
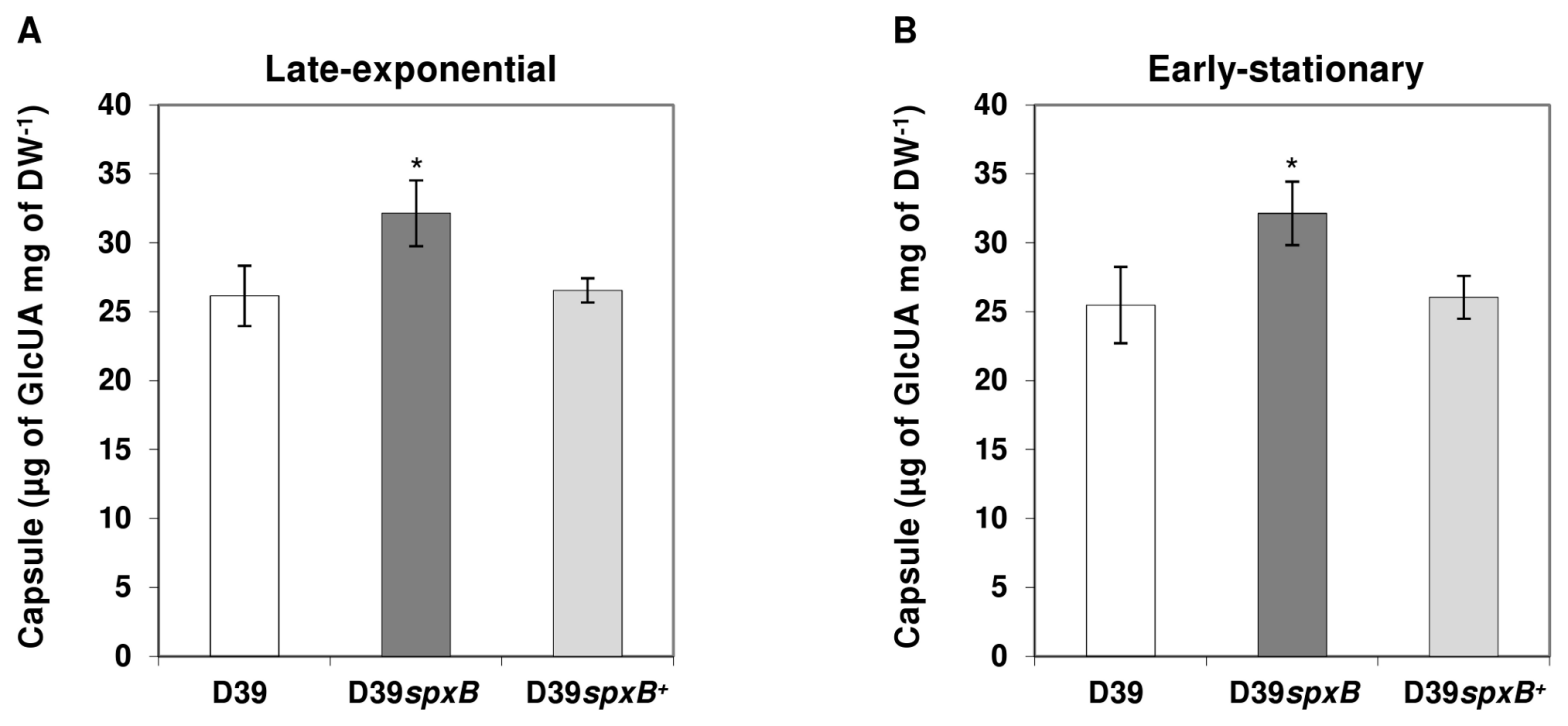
Effect of *spxB* mutation on capsule production. Estimation of capsule was performed based on the determination of its glucuronic acid content in strains D39 (white bars), D39*spxB* (dark grey bars) and D39*spxB* ^*+*^ (complemented strain) (light grey bars) in late-exponential (A) and early-stationary (B) phases of growth. Cultures were grown in CDM containing 1% (wt/vol) Glc at 37^°^C, with pH control (6.5), and under semi-aerobic conditions (for details see Materials & Methods). Determinations were done twice in three independent cultures and the values are means ± SD. * = P≤0.01 students t-test. For D39*spxB* ^*+*^, the capsule was determined twice for each of two independent cultures.

The observed phenotype of the colonies (large, mucoidal and slimy) could be due to the reported absence of death in stationary phase of *spxB* mutants and the ensuing increase in biomass production over time [24] or due to increased capsule production. Therefore, the amount of capsule was determined in both the wild type and the *spxB* mutant (D39*spxB*) in late-exponential (Figure 2A) and early-stationary phases of growth (Figure 2B) in Glc-CDM. The data showed that the amount of capsule in the D39*spxB* mutant significantly increased by circa 30% relative to the wild type levels (students t-test P≤0.01), meaning that the observed larger colonies are to some extent due to increased capsule amounts rather than differences in survival. Furthermore, TEM pictures of bacteria grown to exponential phase in liquid medium, also indicated an increased amount of capsule in the mutant (Figure 1C, 1D) as well as some subtle changes in the cell membrane and or peptidoglycan (Figure 1E, 1F).

In order to determine that the observed phenotype was indeed due to the inactivation of *spxB*, complementation of the *spxB* mutation was performed, which restored the production of SpxB to levels comparable to that of the wild type (Figure S1). The levels of H_2_O_2_ measured (41 ± 4 µM and 80 ± 22 µM at late-exponential and early-stationary phases of growth, respectively) in the complemented strain, indicate restoration of SpxB activity. Importantly, capsule amounts were restored back to the wild type levels in the complemented strain (D39*spxB* ^*+*^) (Figure 2). Thus, in the conditions studied our results show that inactivation of *spxB* increased the amount of capsule in *S. pneumoniae*. In this light, a role of the gene downstream of *spxB*, *spd0637*, in the appearance of the phenotype can be ruled out. Mutations of *spxB* or reduction in SpxB activity resulting in mucoid and or opaque-like appearances is a recurrent observation [18,28,31,32].

### Transcription of capsule (*cps*) operon is increased in the *spxB* mutant

**Figure 3:**
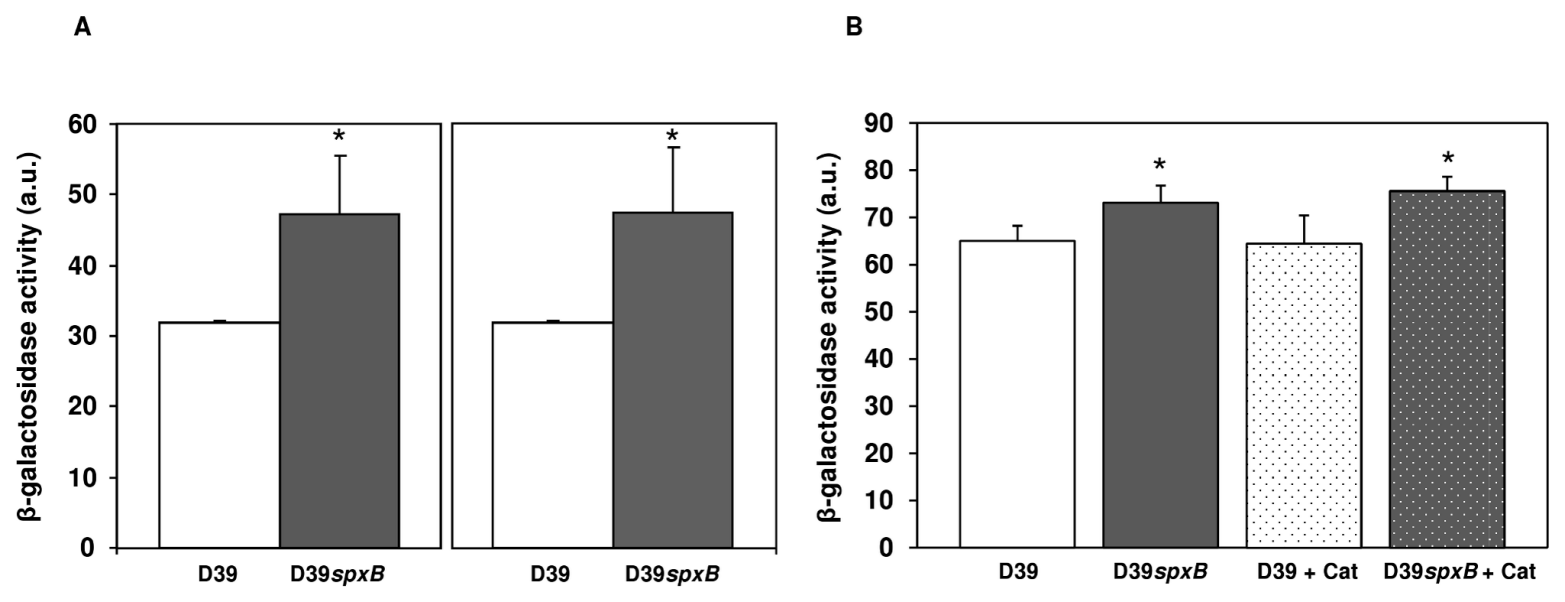
Effect of *spxB* mutation and addition of catalase on *cps* transcription. Transcription of the *cps* promoter was estimated by measuring the β-galactosidase activities of strains D39 (bars with white background), D39*spxB* (bars with grey background) and D39*spxB* and its parent in the presence of 200 U/ml bovine liver catalase (spotted bars) harbouring pORI P*cps* (see Table 1) in mid-exponential (A, left panel and B) and late-exponential (A, right panel) phases of growth. Cultures were grown in CDM containing 1% (wt/vol) Glc (A) or in BHI (B) at 37^°^C, and under semi-aerobic conditions. All the determinations were done at least in triplicate and the values are means ± SD. a.u. = arbitrary units.

Next we determined whether the increase in capsule production was mediated by changes in transcription of the *cps* operon by determining the activity of the *cps* promoter, localized upstream of the first gene, *cpsA*. A fusion with a promoterless *lacZ* was generated in pORI13 [33] and introduced into D39 and the D39*spxB* mutant. Both in mid-exponential phase as well as late-exponential phase, transcription of the *cps* promoter was increased in the D39*spxB* mutant by about 45% relative to the wild type *cps* transcriptional levels in Glc-CDM, which was statistically significant in each case (Figure 3A). Thus, the increased capsule production is at least partially mediated by a change in transcription of the capsule *locus*. To investigate whether the increased transcription of *cps* was due to oxidative stress induced by external H_2_O_2_ or the metabolic activity of SpxB, D39 and the D39*spxB* mutant were grown with and without 200 U/ml bovine liver catalase in BHI. *Cps* transcription in the wild type was not increased to the level of the *spxB* mutant by the detoxification of H_2_O_2_ indicating that the effect is not mediated by the presence of this reactive oxygen species (Figure 3B). Determination of *cps* promoter transcriptional levels of D39 and *spxB* in the presence of catalase was peformed by growing the cells in a complex medium (BHI) (Figure 3B) rather than CDM (Figure 3A). The difference in medium composition might explain the lower induction ratio (about 15%) of *cps* transcription in the *spxB* mutant observed in BHI (Figure 3B) as compared to that in CDM (45% induction) (Figure 3A).

### Deletion of *spxB* has only a minor influence on the transcriptome of D39

To obtain a better insight into why inactivation of *spxB* resulted in an increase in capsule production, the transcriptome of the *spxB* mutant was compared to that of the wild type strain under semi-aerobic conditions (Table 1). Interestingly, inactivation of *spxB* had only a modest influence on the transcriptome of *S. pneumoniae* (Table 1). However, in line with its role in metabolism [19,32,34], two divergent putative operons that are likely involved in carbohydrate metabolism were highly induced (Table 1). The first one (*spd0289* to *spd0292*) contains genes whose homology indicates involvement in the catabolism of ketogluconate [35]. The second putative operon encodes four genes (*spd0293, spd0295-7*) showing homology to a mannose-family PTS system that is predicted, based on homology of the IIC and IID components, to use as substrate N-acetylglucosamine (TransportDB, www.membranetransport.org), and one gene, *spd0294* encoding a glucuronyl hydrolase [36]. A recent report provided evidence for the involvement of the Man-family PTS in the utilization of hyaluronic acid, a glycosaminoglycan consisting of repeating disaccharides, which are composed of D-glucuronic acid and D–N-acetylglucosamine [37]. Contrary to the expectations, no changes were observed in genes of the capsule *locus*. This could be due to the modest increase in capsule amount, below 2 fold, which was the cut off value used.

**Table 1:** Genes differentially regulated in the D39*spxB* mutant grown in Glc-M17.

***SPnumber***	***SPDnumber***	***TIGRannotation***	***Foldchange***	***Bayes.p***
SP0095	SPD0091	conserved hypothetical protein	0.4	8.38E-08
SP0317	SPD0289	4-hydroxy-2-oxoglutarate aldolase/2-deydro-3-deoxyphosphogluconate aldolase	7.4	3.00E-15
SP0318	SPD0290	carbohydrate kinase, PfkB family	9.5	0
SP0319	SPD0291	conserved domain protein	9.6	0
SP0320	SPD0292	oxidoreductase, short chain dehydrogenase/reductase	8.6	0
SP0321	SPD0293	PTS system, IIA component	2.3	7.08E-07
SP0322	SPD0294	glucuronyl hydrolase	3.5	4.66E-12
SP0323	SPD0295	PTS system, IIB component	2.6	4.30E-07
SP0324	SPD0296	PTS system, IIC component	4.6	4.54E-09
SP0325	SPD0297	PTS system, IID component	10.2	0
SP0506		integrase/recombinase, phage integrase family	2.4	1.67E-06
SP0626	SPD0546	branched-chain amino acid transport system II carrier	0.3	3.61E-11
SP0731	SPD0637	conserved domain protein	0.3	2.22E-16

Genes were considered differentially expressed when the fold change was ≥ 2, ≤ 0.5 with a Bayes *p* ≤ 0.00001

It should be noted that the insertion of the ISS1 element at the 3’ end of the *spxB* gene yielded no changes in the transcription level of the gene. Although *spxB* and its downstream gene s*pd0637* do not appear to be located in an operon, expression of s*pd0637* was significantly reduced in the mutant. Theoretically, this could also be the cause of the observed effects of the ISS1 insertion into *spxB*. However, complementation of the *spxB* mutation reverted the phenotype of the colonies back to the wild type appearance, indicating that s*pd0637* repression played no role in the increase in capsule production.

### Growth and fermentation profiles are not affected by *spxB* inactivation under semi-aerobic conditions

**Figure 4:**
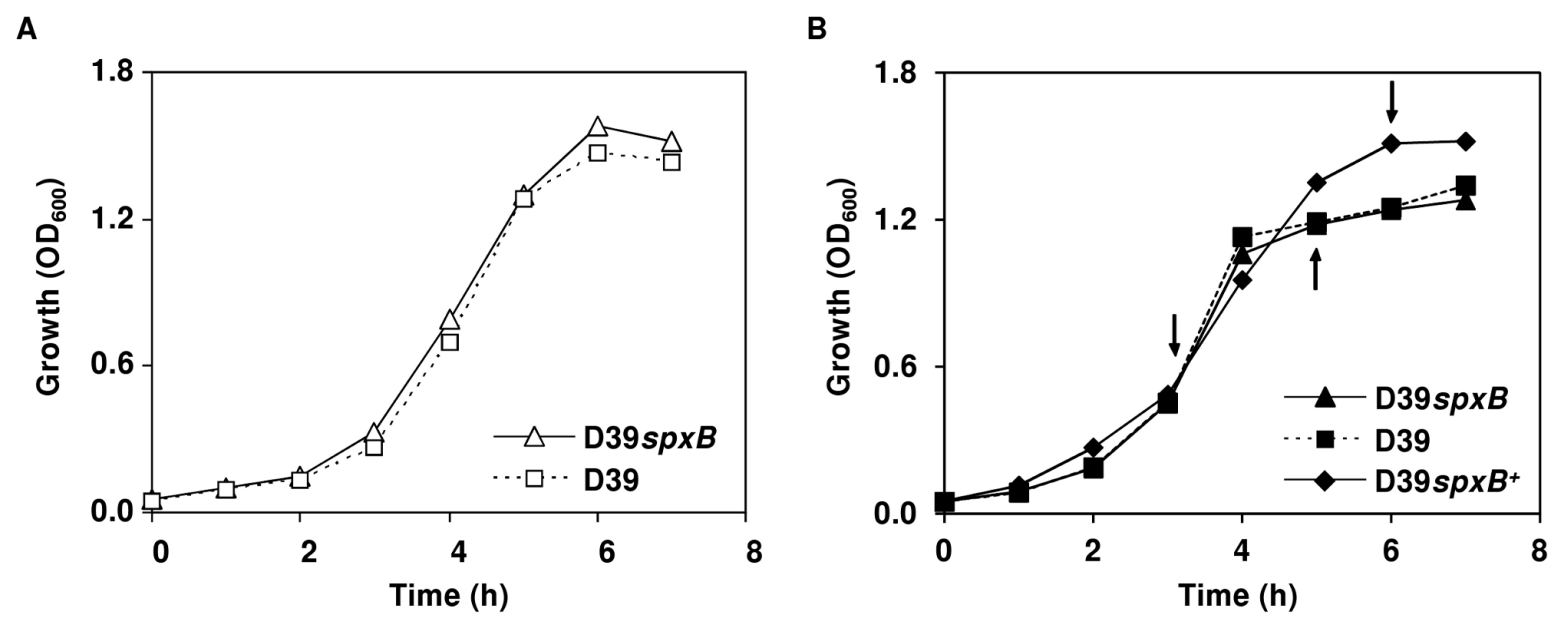
Growth profiles of strains D39, D39*spxB* and D39*spxB* ^*+*^ under semi-aerobic conditions. Cultures were grown in A) M17 without pH control (initial pH 6.5) or B) CDM with pH control (pH 6.5), with 1% (wt/vol) Glc at 37^°^C, in both cases under semi-aerobic conditions (for details see Materials and Methods). The plotted growth curves are from a representative experiment. For each condition at least three independent experiments were performed, except for the complemented strain (D39*spxB* ^*+*^) which was performed twice, and the error was below 15%. Symbols: (triangles), D39*spxB*; (squares), D39; (diamonds), D39*spxB* ^*+*^. Arrows in the CDM grown cultures indicate sampling time for capsule determination and intracellular metabolite analysis (Figures 2 and 5).

Given the metabolic function of SpxB and in order to explore a potential connection between carbohydrate metabolism and capsule production, growth properties of D39*spxB* and its parent strain D39 were determined in Glc-M17 as this was the medium in which the original phenotype was observed. Under these conditions, the *spxB* mutation had no significant effect on the growth profile or fermentation type of *S. pneumoniae* D39 (Figure 4A and Table 2). Both D39 and its *spxB* derivative grew to maximal OD_600_ of about 1.5 in 6 h at similar specific growth rates (Table 2), showing a typical homolactic fermentation pattern (Table 2), with lactate accounting for about 90% of the glucose consumed. Considering the absence of major differences in growth characteristics of strain D39 and its D39*spxB* mutant in the complex medium M17, we wondered whether in a chemically defined medium (CDM) the loss of the metabolic enzyme SpxB would have a more pronounced effect. Furthermore, the use of CDM not only facilitates the interpretation of results, but also allows the use of NMR techniques for metabolic characterization.

**Table 2:** Effect of *spxB* deletion on the end-product yields and growth and energetic parameters of D39 grown in Glc-M17 and Glc-CDM under semi-aerobic conditions^a^.

	**Semi-aerobiosis**			
	**Glc-M17**		**Glc-CDM**	
	**D39*spxB***	**D39**	**D39*spxB***	**D39**
**Product yields^b^**				
Lactate	1.75 ± 0.06	1.78 ± 0.02	1.69 ± 0.03	1.63 ± 0.01
Formate	0.05 ± 0.05	0.10 ± 0.03	0.08 ± 0.01	0.06 ± 0.01
Pyruvate^c^			0.09 ± 0.02	0.04 ± 0.01
Acetate	BDL	BDL	BDL	BDL
**H_2_O_2_ (µM)**	ND	ND	BDL	10 ± 0
**Consumed substrate (%)**	36 ± 2	35 ± 1	28 ± 1	28 ± 0
**Carbon balance^d^**	88 ± 1	89 ± 1	93 ± 1	87 ± 1
**Redox balance^e^**	84 ± 3	83 ± 1	88 ± 1	85 ± 1
**Biomass yield (g mol^-1^ of Glc)**	24.8 ± 0.4	23.7 ± 0.2	24.9 ± 3.1	26.2 ± 1.0
**ATP yield (mol mol^-1^ of Glc)**	1.8 ± 0.0	1.8 ± 0.1	1.8 ± 0.1	1.7± 0.0
**Y_ATP_ (g of biomass mol^-1^ of ATP)**	14.1 ± 0.2	13.3 ± 0.1	14.2 ± 1.3	15.0 ± 0.7
**µ_max_ (h^-1^)**	0.85 ± 0.01	0.87 ± 0.06	0.70 ± 0.05	0.83 ± 0.04

a D39 and D39*spxB* were grown in complex medium (M17) or chemically defined medium (CDM) containing 1% (wt/vol) glucose, under semi-aerobic conditions as described in materials and methods. Product yields and growth and energetic parameters were determined from substrate and fermentation product analysis by HPLC at the time-point of maximal biomass achieved by both strains;

b Product yields: [End-product, mM]/[Glucose consumed, mM];

c Blank cells, negative yields were found for these conditions (cells used pyruvate from the medium);

d Carbon balance is the percentage of carbon in metabolized Glc that is recovered in the fermentation products (lactate and formate) and pyruvate;

e Redox balance is the ratio between [lactate] and 2 x [glucose] multiplied by 100;

Dry weight (DW) was used as a measure of cell mass. ND, not determined. BDL, below detection limit;

Values of at least two independent experiments were averaged and errors are reported as ± SD.

Strains D39 and D39*spxB* were grown in Glc-CDM (Figure 4B, Table 2). The specific growth rates and maximal OD_600_ were lower in Glc-CDM than in the complex medium (Glc-M17). In the defined medium D39 and D39*spxB* displayed identical growth properties, with respect to the biomass yield, lactate and formate yields, and bioenergetic parameters (Table 2). Strain D39 presented, however, a maximal growth rate significantly higher (P value of 0.002) than D39*spxB*. Consistent with inactivation of pyruvate oxidase, the pyruvate yield was 2-fold higher in the D39*spxB* mutant (Table 2). Also in agreement, H_2_O_2_ (about 10 µM) was detected in the extracellular medium during the growth of the parent strain, but not in D39*spxB*. These data corroborate the loss of SpxB function in our mutant strain. It is plausible to assume that the low H_2_O_2_ concentration measured under semi-aerobic conditions arose from a combination of factors: the low dissolved oxygen and H_2_O_2_ decomposition by the action of protection mechanisms against its toxicity. Indeed, we are not the first to report lower H_2_O_2_ production in *S. pneumoniae* than that expected from stoichiometry [34]. Curiously, Glc was still abundant when growth ceased (70% of the initial Glc remained in the culture medium, Table 2). Strains D39 and D39*spxB* were grown under controlled neutral pH, thus growth arrest was likely due to a nutritional limitation other than Glc. Overall, *spxB* mutation slightly decreases the growth rate of D39 under semi-aerobic conditions and increases pyruvate accumulation during Glc metabolism, but does not cause a major change in the fermentation profile.

### Inactivation of *spxB* affects the pools of intracellular metabolites

**Figure 5:**
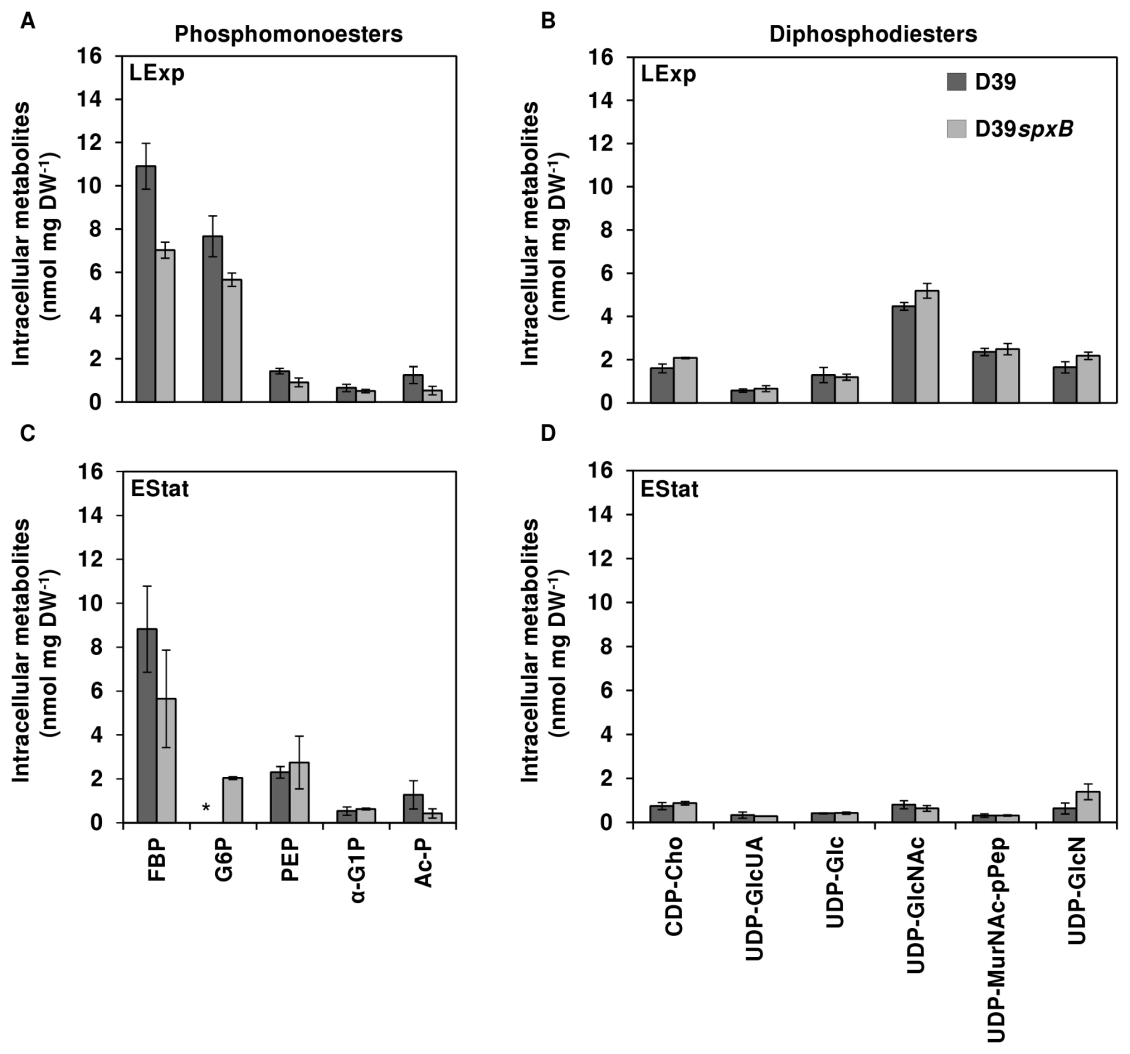
Effect of *spxB* mutation on intracellular phosphorylated metabolites during growth in Glc-CDM under semi-aerobic conditions. Phosphorylated metabolites were measured by ^31^P-NMR in ethanol extracts of D39 and D39*spxB* cells grown to late-exponential (LExp) (A, B) and early-stationary (EStat) (C, D) phases of growth in CDM supplemented with 1% (wt/vol) Glc as in Figure 4B. The results are averages of three to four independent growths and the average accuracy was ± 15%. Symbols: (dark grey bars), D39, (light grey bars), D39*spxB*. FBP, fructose 1,6-bisphosphate; G6P, glucose 6-phosphate; PEP, phosphoenolpyruvate; α-G1P, α-glucose 1-phosphate; Ac–P, acetyl-phosphate; CDP-Cho, CDP-choline; UDP-GlcUA, UDP-glucuronate; UDP-Glc, UDP-glucose; UDP-GlcNAc, UDP-N-acetylglucosamine; UDP-MurNAcp-Pep, UDP-N-acetylmuramoyl-pentapeptide; UDP-GlcN, UDP-glucosamine. LExp, late-exponential phase of growth; EStat, early-stationary phase of growth; A and C, phosphomonoesters; B and D, diphosphodiesters; (*), quantification was impaired by overlapping resonances in NMR spectra.

**Figure 6:**
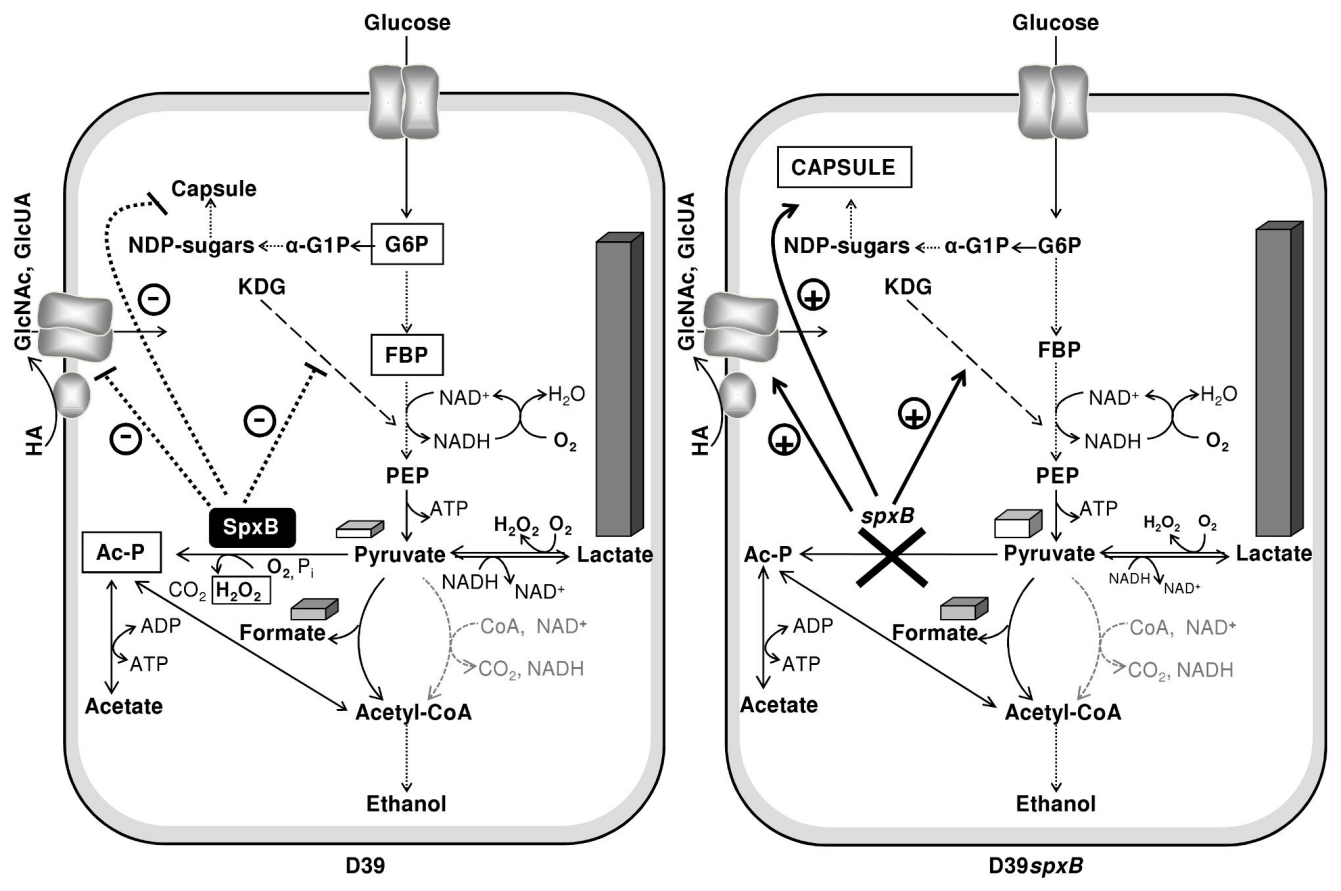
Model of the *spxB* deletion effect on glucose metabolism and capsule production in *S. pneumoniae*. Glucose is converted by the conventional glycolytic pathway to pyruvate. Fermentation products result from the action of different competing enzymes (lactate dehydrogenase, pyruvate formate-lyase, pyruvate oxidase and hypothetically the pyruvate dehydrogenase complex). Overall, our data show that SpxB represses carbohydrate specific pathways (ketogluconate and hyaluronic acid utilization), capsule production, and stimulates formation of acetyl-P and acetate at the expense of pyruvate and lactate. Pyruvate and end-products are represented by three-dimensional bars and the height of the bar represents the relative amount; grey slashed arrows, function not experimentally verified; boxed metabolites indicate higher metabolite accumulation. Thick pointed-line, repression; Thick black arrow, activation. KDG, ketogluconate; HA, hyaluronic acid; GlcNAc, N-acetylglucosamine, GlcUA, glucuronic acid; Ac–P, acetyl-phosphate; α-G1P, α-glucose 1-phosphate; G6P, glucose 6-phosphate; FBP, fructose 1,6-bisphosphate; PEP, phosphoenolpyruvate.

Our data showed that the *spxB* mutation had a modest impact on growth and fermentation products in cultures under semi-aerobic conditions, but clearly affected the production of the polysaccharide capsule (Figure 2). The disparate capsule production could suggest an altered intracellular metabolism. In line, the specific growth rate of D39*spxB* was slightly, but consistently, lower than that of D39. To verify this hypothesis we measured the intracellular metabolites at the partitioning node between glycolysis and the polysaccharide 2 biosynthetic pathway, as well as several NDP-sugars, including the capsule precursors UDP-Glc and UDP-GlcUA (Figure 5). Metabolite concentrations were determined in ethanol extracts obtained during late-exponential and early-stationary phases of growth by ^31^P-NMR (see the time-points indicated by the arrows in Figure 4B). Interestingly, the loss of *spxB* led to a considerable reduction in the levels of the upper glycolytic metabolites glucose 6-phosphate (G6P) and fructose 1,6-bisphosphate (FBP) in late-exponential phase (Figure 5A). G6P is a key metabolite at the hub of glycolysis and several biosynthetic pathways, and its conversion to α-glucose 1-phosphate (α-G1P) is the first step that commits it to the synthesis of many structural polysaccharides, including type 2 polysaccharide (Figure 6). The intracellular concentration of α-G1P was similar in D39 and its D39*spxB* mutant. The same was true for the UDP-activated capsule precursors, UDP-Glc and UDP-GlcUA (Figure 5). These data indicate a redirection of carbon away from glycolysis in the D39*spxB*, and is consistent with the higher capsule production. In addition, no major differences were detected between the levels of other NDP-activated metabolites in D39 and the D39*spxB* mutant. As expected, the product of SpxB activity, the high energy phosphoryl donor acetyl-P (Ac-P), was reduced in the mutant by 65% of the parent strain (Figure 5), which is in the same range as the values recently reported by Ramos-Montañez et al. [32]. Similarly, in the late-exponential phase of growth, the ATP levels were not affected by the loss of SpxB activity (2.3 ± 0.3 and 2.1 ± 0.2 nmol mg DW^-1^, respectively). In general, the intracellular concentrations of phosphorylated metabolites in both strains were lower in early-stationary phase, except for phosphoenolpyruvate (PEP), which increased slightly.

### SpxB skews the sugar utilization profile of *S. pneumoniae*

**Figure 7:**
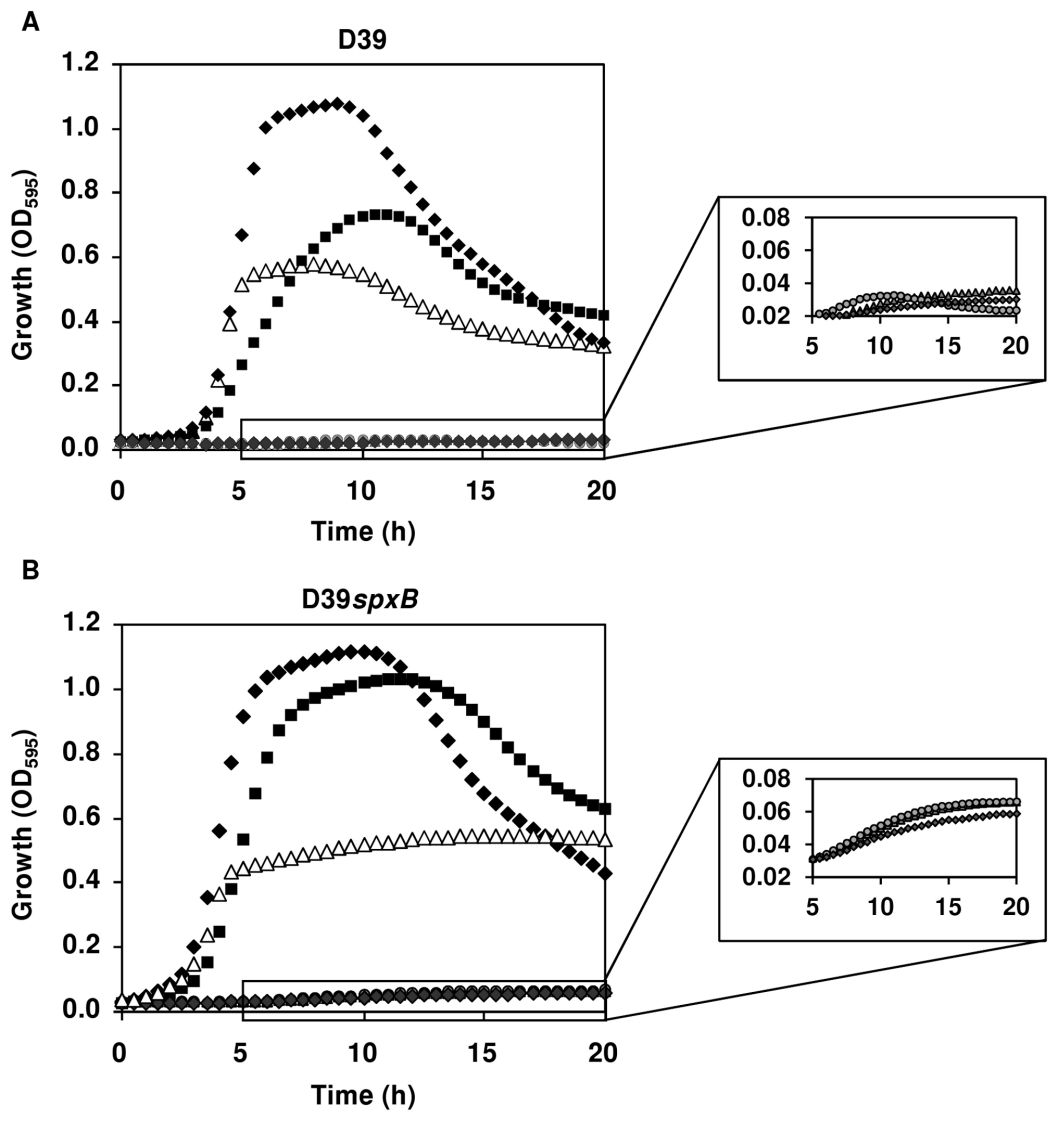
Effect of s*pxB* mutation on the ability to grow on specified sugars. Growth profiles of *S. pneumoniae* D39 (A) and its derivative D39*spxB* (B) in CDM containing 0.25% (wt/vol) of the specified sugar. Cultures were prepared in 250 µl in 96-well microtiter plates and growth monitored at 595 nm and 37^°^C. Symbols: (black diamonds), Glc; (grey diamonds), GlcUA; (grey triangles), Rha; (grey circles), GlcA; (black squares), GlcNAc; (white triangles) 1 GlcUA : 2 Glc: 3 Rha. Zoomed areas: expansions of time-points 5 to 20 h for growths in rhamnose, gluconic acid and glucuronic acid. Each point of the growth curves is an average of triplicate experiments each consisting of two independent cultures, and the error was in all cases below 20%, except for rhamnose which was below 30%.

The fact that the majority of transcriptomic differences between D39 and D39*spxB* were observed for genes potentially involved in central metabolism, and in particular sugar catabolic pathways, prompted us to analyze the growth of the two strains on selected sugars. Genome annotation and homology studies suggested that the genes differentially expressed might play roles in the catabolism of N-acetylglucosamine (GlcNAc), glucuronic acid (GlcUA) and/or gluconic acid (GlcU). Since loss of *spxB* leads to higher amount of capsule polysaccharide, and considering the higher expression of a putative glucuronyl hydrolase (*spd0294*) and the PTS system needed to import its products [37] we surmised an aptitude of the D39*spxB* strain to utilize type 2 polysaccharide derived sugars (GlcUA, Glc, and rhamnose, Rha) as carbon sources. Thus, rhamnose and a sugar mixture mimicking the type 2 capsule (1 GlcUA: 2 Glc: 3 Rha) were also tested. Growth was performed in CDM supplemented with 0.25% (wt/vol) sugar in 96-well microtiter plates (Figure 7), under semi-aerobic conditions. As expected, on Glc D39 showed a modest, but significant (P value < 0.05) higher specific growth rate than the D39*spxB* strain (1.18 ± 0.06 as compared to 0.91 ± 0.06 h^-1^). The maximal OD_595_ was similar for both strains on Glc (about 1.1). Growth on GlcNAc was characterized by identical specific growth rates (around 0.83 h^-1^) for both strains, but lack of *spxB* resulted in 25% increase in the final biomass (from OD_595_ of 0.75 to 1.03). This result supports a role of the PTS system (*spd0293-0297*), which is differentially expressed in our transcriptomic analysis, in the catabolism of GlcNAc. In the sugar mixture, both strains displayed identical specific growth rates and maximal biomass; however, while the OD_595_ values of D39 decreased upon growth arrest, the D39*spxB* mutant showed a slow but steady increase of the OD_595_ values during the time of monitoring (24 h). Also, the D39*spxB* strain showed residual growth on GlcUA, Rha and GlcU, while D39 was totally unable to use these sugars as sole carbon sources for growth. These data strongly indicate that *spxB* considerably influences the nutritional utilization capabilities of *S. pneumoniae*.

### Oxygen affects differently end-product distribution in strain D39 and *spxB* mutant under aerobic conditions

**Figure 8:**
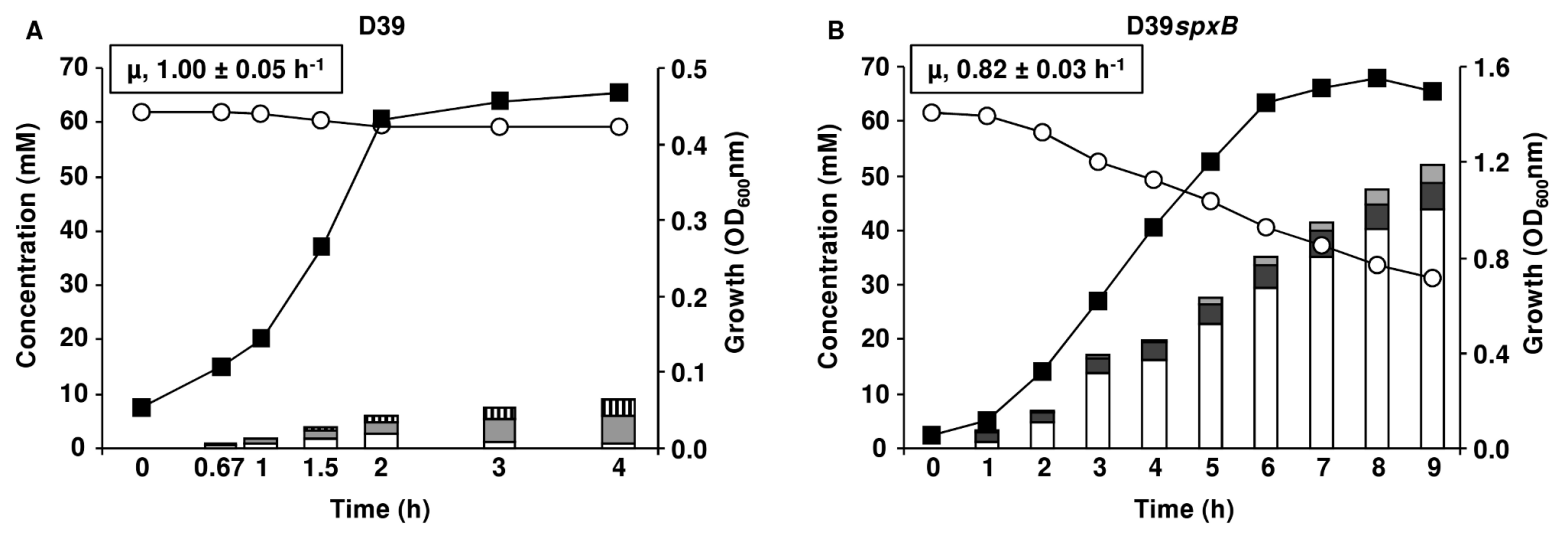
Growth and fermentation profiles in *S. pneumoniae* D39*spxB* and its parent strain D39 under aerobic conditions. Growth curves, substrate consumption and end-products formed by D39 (A) and D39*spxB* (B) strains growing on Glc-CDM, at 37^°^C, with pH control (6.5), and under aerobic conditions (for details see supplemental Materials and Methods S1). Culture supernatant samples for substrate and end-product analysis by HPLC and/or ^1^H-NMR were harvested at denominated time-points along growth (bars in the plots). The plotted growth curves, substrate consumption curves and end-products bars are from a representative experiment. For each condition at least two independent experiments were performed. For the growth and substrate consumption curves the error in each point was below 10%. For the end-products concentrations, the error was below 7% for major products (> 3.5 mM) and 30% for minor products (< 3.5 mM) Symbols: (white circles), substrate consumption; (black squares), growth curve; white bars, lactate; dark grey bars, pyruvate; light grey bars, acetate; stripped bars, hydrogen peroxide. The data on D39 is also presented in [67].

Most studies on SpxB were performed under semi-aerobic conditions and showed a modest effect of SpxB on metabolism. However, molecular oxygen is the substrate for the SpxB catalyzed reaction. Hence, to further explore the role of SpxB activity in pneumococcal carbohydrate metabolism under highly oxygenated conditions that may occur during transmission or on the surface of the mucosa, we grew strain D39 and its derivative *spxB* mutant under a continuous specific air tension of 40% (constant dissolved oxygen concentration of 90 µM at 37^°^C). We expected visible changes in end-product profiles in strain D39, but minor or no changes in D39*spxB*. Indeed, the increase in oxygen availability was translated into a substantial rise in H_2_O_2_ production to about 2 mM in the parent strain D39. The concentration of hydrogen peroxide in supernatants of strain D39*spxB* was in the micromolar range (2 µM), three order of magnitude lower than in D39 (Figure 8 and Table 3). Under aerobic conditions, the maximal biomass reached for strains D39 and D39*spxB* was 0.49 ± 0.03 (time-point 3h) and 1.64 ± 0.13 (time-point 8h), respectively (Figure 8). At these time-points the D39*spxB* mutant consumed approximately 50% of the Glc supplied and the control strain only about 4% (Table 3). Under these conditions, the low biomass generated by D39 is most likely a consequence of high H_2_O_2_ accumulation due to SpxB activity. Loss of *spxB* caused a significant decrease (about 20%) in the maximal growth rate (Table 3), which suggests that SpxB activity stimulates faster growth on Glc under highly oxygenated conditions.

**Table 3:** Effect of *spxB* deletion on the end-product yields and growth and energetic parameters of D39 grown in Glc-CDM under aerobic conditions^a^.

	**Aerobiosis**	
	**Glc-CDM**	
	**D39*spxB***	**D39**
**Product yields^b^**		
Lactate	1.47 ± 0.02	0.47 ± 0.09
Formate	BDL	BDL
Pyruvate^c^	0.16 ± 0.01	
Acetate	0.11 ± 0.00	1.53 ± 0.09
H_2_O_2_	BDL	0.82 ± 0.01
**Consumed substrate (%)**	49 ± 0	4.4 ± 0.6
**Carbon balance^d^**	87 ± 2	100 ± 0
**Redox balance**	ND	ND
**Biomass yield (g mol^-1^ of Glc)**	20.9 ± 2.6	68.1 ± 2.4
**ATP yield (mol mol^-1^ of Glc)**	1.9 ± 0.0	3.5 ± 0.1
**Y_ATP_ (g of biomass mol^-1^ of ATP)**	11.3 ± 1.1	19.3 ± 1.1
**µ_max_ (h^-1^)**	0.82 ± 0.03	1.00 ± 0.05

a D39 and D39*spxB* were grown in chemically defined medium (CDM) containing 1% (wt/vol) glucose, under aerobic conditions (constant air tension of 40%) as described in materials and methods. Product yields and growth and energetic parameters were determined from substrate and fermentation product analysis by HPLC at the time-point of maximal biomass achieved by both strains;

b Product yields: [End-product, mM]/[Glucose consumed, mM];

c Blank cells, negative yields were found for these conditions (cells used pyruvate from the medium);

d Carbon balance is the percentage of carbon in metabolized Glc that is recovered in the fermentation products (lactate and acetate) and pyruvate; Dry weight (DW) was used as a measure of cell mass. ND, not determined. BDL, below detection limit;

Values of at least two independent experiments were averaged and errors are reported as ± SD.

Before reaching stationary phase D39 produced in continuous increments the end-products acetate, H_2_O_2_ and lactate, from Glc, which reached at the time-point of growth arrest (maximal biomass) concentrations of 4.2 ± 0.1 mM, 2.2 ± 0.1 mM and 1.3 ± 0.3 mM, respectively. Interestingly, in the stationary phase the levels of acetate and H_2_O_2_ increased at the expense of lactate, suggesting the occurrence of lactate oxidase activity in this condition (Figure 8). As expected, loss of *spxB* led to a typical homolactic behavior, with lactate as major end-product (maximal concentration 44 ± 0 mM), accounting for 75% of the Glc consumed (Table 3); acetate was also formed in minor amounts and the yield of pyruvate was 2-fold enhanced as compared to semi-aerobic conditions. No formate was observed, suggesting suppression of pyruvate formate-lyase (PFL) activity when the oxygen concentration is kept steady around 0.09 mM. In the D39*spxB* mutant, the H_2_O_2_ detected was below 10 µM. Detection of acetate in the *spxB* mutant while *spxB* is inactivated and PFL suppressed by O_2_ indicates an alternative pathway for the formation of this organic acid. In summary, our data show that the metabolic response to highly oxygenated conditions is largely mediated by SpxB, since growth and fermentation profiles of strain D39*spxB* were barely affected under aerobic conditions.

## Discussion

The role of pyruvate oxidase (SpxB) in the aerobic metabolism of several Gram-positive bacteria is well established [38–41]. In the human pathogen *S. pneumoniae*, a number of studies describe the involvement of SpxB in virulence [17,20,27], fermentation [34] and colony phenotypic variation [16,18,31,42]. However, in depth investigation of the molecular mechanisms underlying these modes of action is far from complete.

In this study we show that inactivation of *spxB* leads to an increase in capsule production. High capsule amounts have been consistently associated with opaque phase variants, which are interestingly also characterized by low SpxB activity [13,14,16]. However, a comparison of phenotypes on plate showed that, although displaying similar properties, the *spxB* mutant is not an opaque variant. Nevertheless, the observation that deletion of *spxB* or a reduction in SpxB activity leads to an opaque-like or mucoid phenotype on plate is recurrent [16,28,31,32]. Additionally, other disruptions in pathways leading to acetate and ATP production also seem to interfere with capsule biosynthesis, as suppressor mutations of *ackA* mutations often resulted in an inactivation of both *spxB* and capsule production [32]. Our data show for the first time, that the mucoid phenotype appearance of D39 colonies lacking SpxB activity is due to an increase in capsule biosynthesis, as indicated by the higher expression levels of the *cps* promoter and glucuronic acid amounts, and is independent of differences in growth or death rate.

The presence of capsule in *S. pneumoniae* is required for both colonization and invasive disease [3,4,10,15,45]. Too much capsule is detrimental for colonization. On the other hand, a thick capsule is optimal for invasive disease, as demonstrated by comparing transparent and opaque variants [15]. Interestingly, a number of studies report that deletion of *spxB* promotes invasive disease in bacteremia models [27] and leads to a defect in nasopharyngeal colonization [17,24,27,28]. The data from our study suggests that this behaviour can partially be due to the increased amounts of capsule present in the D39*spxB*. In concordance with this idea, *spxB* expression is higher in the nasopharynx than in the lungs and the blood [43], probably ensuring that not too much capsule is being produced. Also, *spxB* is higher expressed in transparent variants than in opaque ones, which are poor colonizers [16,44]. It should be noted that although our data show modest changes in capsule amounts between D39 and D39*spxB*, we believe that they are relevant *in vivo* as even relatively small differences in the amounts of capsule can be critical for the expression of virulence [13]. Thus, we would like to propose that SpxB activity in response to the oxygen concentration of its environment is an important signal for *S. pneumoniae* to determine the amount of capsule that needs to be produced. This hypothesis is strengthened by the observation that changes in oxygen seem to induces changes in capsule production in opaque variants [29]. Furthermore, transparent variants were predominantly isolated from the nasopharynx and opaque variants from blood [29]. Growth of D39 under anaerobic conditions also led to a uniform mucoid appearance of the colonies on blood agar plates and capsule amounts similar to those of D39*spxB* in chemically defined medium (data not shown). Additionally, growth of the bacteria in the presence of catalase did not increase *cps* transcription in D39, indicating that the effect is specific for SpxB activity and not by oxidative stress brought about by external H_2_O_2_. How this signaling would occur is currently unclear, one possibility is that the changes in the membrane induced by SpxB activity [25,26] mediate alterations in the production and expression of the capsule *locus*. Capsule synthesis is tightly interconnected with peptidoglycan synthesis and is partly regulated by membrane proteins [45], thus it is likely that disturbances in the membrane or cell wall influence capsule synthesis and vice versa. Although not conclusive, the high magnification TEM pictures of the D39*spxB* mutant suggest subtle changes in the membrane and or peptidoglycan, which could be mediated by *spxB* inactivation and thus result in a change in capsule production.

In other streptococci, CcpA, the main regulator of carbon metabolism in *S. pneumoniae* [46] and other Gram-positive bacteria, represses *spxB* [47,48]. Although there is no *cre* site in the *S. pneumoniae spxB* promoter and the gene was also not found in extensive transcriptome analysis [46], we did attempt to generate a *ΔccpA* mutant in the D39*spxB* background. This failed unless the *ccpA* gene was already ectopically present in the D39*spxB* mutant, indicating that this combination is lethal for *S. pneumoniae* (data not shown). Thus, in *S. pneumoniae* CcpA does not regulate *spxB*, but repression (or activation) of a product of the CcpA regulon does seem necessary to counteract deleterious effects of *spxB* inactivation.

Another alternative to mediate this regulation is SpxR, however no capsule genes were differentially regulated in the transcriptome analysis of this mutant, making it a less likely candidate [28]. It is worth mentioning that while Ramos-Montañez and co-workers used D39 strain NCTC7466 as genetic background, in our work we have characterized a strain with characteristics of a Lilly derivative, at least in what concerns pyruvate oxidase activity [49]. Lilly derivatives show a considerably lower SpxB activity than NCTC strains [28]. Thus, a direct extrapolation from previous work performed with D39 NCTC might not be possible. Interestingly, transcriptome analysis of our D39*spxB* mutant showed more differentially transcribed genes than those reported for a *spxB* mutant in the NCTC isolate [28]. This could be due to allelic variance between NCTC and our isolate, differences in sampling time (phase of growth) and or in the composition of the medium used for growth. The transcriptome analysis of D39*spxB* showed minor changes (13 altered transcripts) compared to the wild type, despite the fact that the experiments were carried out under semi-aerobic conditions and harvest took place during early-exponential growth phase when oxygen is still thought to be present and SpxB activity can be detected. Loss of *spxB* did not affect the transcript levels of regulatory proteins. These findings reinforce the idea that neither SpxR nor CcpA play prominent roles in the signaling pathways mediating the SpxB phenotypes under these conditions.

Of the genes that were differentially expressed, 77% encode functions involved in central metabolism (Table 1). Of these, 9 genes organized in two transcriptional units (*spd0289-92*, *spd0293-97*) were highly induced in the *spxB* mutant. *spd0293-97* encodes a Man-family PTS system and a glucuronyl hydrolase, which have recently been implicated in the metabolism of hyaluronic acid, and in particular in the uptake and degradation of GlcNAc-GlcUA disaccharides [37,50]. The four genes in *spd0289-92* are most likely involved in the degradation of ketogluconic acid, as judged by homology comparisons [35]. Thus, a role for SpxB was surmised on the utilization of carbon sources for growth, which was corroborated by the better growth on GlcNAc compared to the wild type and the observed residual growth of the D39*spxB* strain on sugars potentially present in capsules or host glycans (Figure 7). Growing evidence supports the utilization of host glycans as nutritional sources for pneumococcal growth [37,51,52]. To our knowledge, we are the first to show a link between SpxB and carbohydrate utilization. Based on our results, it is tempting to hypothesize that in specific host microenvironments with oxygen where glucose becomes the major carbon source available, for instance during inflammation or hyperglycemia (e.g. lungs during infection) [53], SpxB activity minimizes the use of alternative sugar utilization pathways (repression of specific catabolic steps involved in the metabolism of sugars other than glucose) in a cost effective manner, which will allow for rapid proliferation and fast energy production, i.e. improved fitness (Figure 6).

We found that under semi-aerobic conditions, SpxB slightly stimulated the pneumococcal rate of growth on glucose and inhibited capsule production (Figure 6 and Table 2, Figures 2 and 3). Importantly, the soluble activated precursors for serotype 2 capsule synthesis are all derivatives of α-glucose 1-phosphate, which originates from the action of α-phosphoglucomutase on the glycolytic intermediate glucose 6-phosphate. The lower accumulation of glucose 6-phosphate and fructose 1,6-bisphosphate in the D39*spxB* mutant indicate a redirection of carbon towards biosynthetic pathways, in particular capsule (Figure 6). The data on intracellular metabolites is therefore in full agreement with our findings in respect to capsule production and growth rate, and allows us to propose that the *spxB* mutation affects the intracellular fluxes of *S. pneumoniae*.

Curiously, under the imposed semi-aerobic conditions the fermentation pattern of the pneumococcus was only marginally affected by loss of SpxB, the major difference being a higher accumulation of pyruvate in the mutant (Figure 6). This difference is consistent with a decreased level of Ac–P in D39*spxB*. In bacteria, a role of the central metabolite Ac–P in linking the nutritional status to global signaling, namely by acting as a phosphoryl donor to two-component response regulators has been proposed [54]. One could argue that similar molecular mechanisms could be in effect in *S. pneumoniae*, i.e., Ac–P phosphorylation of response regulators. However, in a recent study, Ramos-Montañez and co-workers showed that of three response regulators only WalRK_*Spn*_ had changes in expression correlating with the amounts of Ac–P; most of their work did not support phosphorylation of regulators by Ac–P as a general signaling mechanism in *S. pneumoniae* [32]. This is in line with the minimal effect of *spxB* deletion on the transcriptome, inferred in our and other studies. Rather than relaying the metabolic state to regulatory pathways, Ac–P is a major energy reservoir for ATP synthesis. In Ramos-Montañez et al. [32], the amount of ATP was not affected by *spxB* deletion. Likewise, in our study loss of *spxB* did not affect the steady-state ATP amount (2.3 ± 0.3 and 2.1 ± 0.2 nmol mg DW^-1^ in exponential cells of D39 and D39*spxB*, respectively).

It has been suggested that maintenance of wild type ATP levels is essential to cope with H_2_O_2_ imposed stress [19,32]. Our observation that the level of H_2_O_2_ produced under semi-aerobic conditions is 10-fold lower than that expected from a stoichiometry of one molecule of O_2_ consumed per molecule of hydrogen peroxide formed attests to the presence of H_2_O_2_ scavenging mechanisms in strain D39. However, the presence of other O_2_ consuming activities, such as NADH oxidase, cannot be ruled out, but the similarity of the carbon and redox balance values observed for strain D39 (Table 2) is in stark disagreement with the presence of high NADH-oxidase activity.

When environmental oxygen is provided continuously (constant partial air tension of 40%), the yield of strain D39 was dramatically reduced to about 30% of the semi-aerobic level, while no major difference in biomass was detected for strain D39*spxB* (Figure 4 and Figure 8). In terms of fermentative products, oxygenation induced a mild shift to acetate production in the *spxB* mutant, denoting an alternative aerobic acetate producing pathway. The presence of a functional pyruvate dehydrogenase complex in the pneumococcus has been suggested, but is still to be proven [32,34]. On the other hand, lactate production was severely decreased and a switch to acetate and H_2_O_2_, which accumulated up to 2-3 mM, was observed in strain D39. This metabolic shift is in accordance with a higher cell demand for ATP, and is well known to occur in other *Streptococcaceae* as a response to stress conditions (aerobiosis, substrate limitation, poor substrates, etc.) [55,56]. Despite the substantially higher yields of acetate in strain D39, the biomass produced was much lower and the ATP concentration in exponential cells was comparable to that under semi-aerobic conditions (data not shown). An important implication from the combined results is that ATP is being redirected to other purposes, seemingly H_2_O_2_ detoxification [19]. This view is further corroborated by the growth properties of strain D39*spxB* under the same conditions.

Despite the modest response of *spxB* deletion in the transcriptome of strain D39 under semi-aerobic conditions, we show in this study that SpxB affects the pattern of carbohydrate utilization in *S. pneumoniae*, and thereby is likely to contribute to the fitness of this microorganism in specific microenvironments. In line, it is reasonable to assume that the influence of environmental oxygen on capsule production and to a lesser extent on growth of *S. pneumoniae* is partially concurred by modulation of pyruvate oxidase activity. Interestingly, oxygen is one of the main environmental factors varying in host niches. In this light, the observed connection between capsule amount and oxygen-dependent SpxB activity is plausible. Based on our data we propose that in the nasopharynx, where the oxygen is high, improved SpxB activity restrains capsule production facilitating effective colonization. In contrast, in more internal host niches with no or very limited oxygen, SpxB activity is reduced which relieves the repressing effect on capsule production. This leads ultimately to a thicker capsule that allows *S. pneumoniae* to better evade the host immune system.

## Materials and Methods

### Bacterial strains and growth conditions

Strains used in this study are listed in Table 4 and were stored in glycerol at -80 °C. *Streptococcus pneumoniae* was routinely grown as standing cultures in M17 broth (Oxoid) [57] containing 0.5% (wt/vol) glucose (Glc-M17) at 37 °C. When necessary erythromycin at a concentration of 0.25 µg ml^-1^ was used. To detoxify the H_2_O_2_ produced, strains were grown in BHI (Oxoid) with or without the addition of 200 U bovine liver catalase (Sigma, St Louis, USA) [58]. BHI medium was used because it lacks antioxidants such as ascorbic acid, which could interfere or obscure effects of the addition of catalase. For growth on plates, 3% (vol/vol) defibrinated sheep blood (Johnny Rottier, Kloosterzande, the Netherlands) was added to Glc-M17 agar.

**Table 4:** Strains and plasmids used in this study.

**Strains**	**Description**	**Reference/Source**
***S. pneumoniae***		
D39	Serotype 2 strain	[68]
TIGR4	Serotype 4 strain	[69]
D39 pGh9 T7	D39 *spxB*: :pGh9:ISS1; Em^R^	This study
TIGR4 pGh9 T7	TIGR4 *spxB*: :pGh9:ISS1T7; Em^R^	This study
D39*spxB*	D39 *spxB*::ISS1	This study
D39*spxB* ^+^	D39*spxB* harbouring pMU1328::300+*spxB*; Em^R^	This study
D39pORI P*cps*	D39 harbouring pORI P*cps-lacZ*; Em^R^	[33]
D39pORI P*cps spxB*	D39 *spxB*::ISS1 harbouring pORI P*cps-lacZ*; Em^R^	This study
**Plasmids**		
pGh9:ISS1T7	Insertion mutagenesis plasmid with T7 RNA polymerase promoter	[30]
pMU1328::300+*spxB*	Plasmid carrying the *spxB* gene under its native promoter; Em^R^	[19]
pORI13	*ori* ^+^ *repA* ^-^ ; promoterless *lacZ*; Em^R^	[70]
pORI P*cps*	pORI13 carrying *cps* promoter in front of the promoterless *lacZ* ; Em^R^	[33]

Abbreviations: Em^R^, erythromycin resistance

### Generation of mutants in *S. pneumoniae*

*S. pneumoniae* D39 was transformed with pGh9 T7 as described before [30]. To excise the plasmid from the genome of *S. pneumoniae*, which leaves behind one copy of the ISS1 element [59], the strain containing the pGh9 T7 plasmid was grown overnight at 28 °C after which serial dilutions were plated on Glc-M17 agar without antibiotics. Erythromycin sensitive colonies were screened for further study. The site of insertion into the genome was determined using a PCR based method as described in [60].

To generate a D39*spxB* Δ*ccpA* mutant the Δ*ccpA* mutation was amplified from chromosomal DNA of strain TIGR4 Δ*ccpA* using primers SP2000 (5' GAACAGTCCGAAACTATGTC 3') and sp1998 (5' ATTCCAGTCGCTCTGGTATC 3') and the resulting PCR product was used for transformation. To generate a *spxB* strain in which the Δ*ccpA* mutation was ectopically complemented, the chromosomal DNA of the corresponding strain was transformed into the *spxB* strain [61].

### Complementation of the *spxB* mutation

To generate a complemented D39*spxB* strain, the pMU1328::300+*spxB* plasmid, containing the *spxB* gene under its native promoter and an erythromicin resistance marker [19], kindly provided by Dr. Jeffrey Weiser, was transformed into D39*spxB*. Erythromycin resistant colonies were selected for further study.

### Transmission electron microscopy of *S. pneumoniae*

Aliquots of *S. pneumoniae* were quickly thawed at 37 °C and diluted 1:40 in Glc-M17 and grown under semi-aerobic conditions at 37 °C until mid exponential phase (OD_600_ 0.2-0.3) Bacteria were harvested by centrifugation for 10 minutes at 3000 x *g* at 4 °C and subsequently stained with lysine ruthenium red (LRR) and treated as described before [12]. For visualization a Philips EM201 (maximum resolution of ~ 0.5 nm) electron microscope was used.

### Determination of transcription using *lacZ*

To measure transcription of the *cps* operon, a pORI13 derivative containing the D39 *cps* promoter was used in the D39 and D39*spxB* backgrounds [33]. Strains were grown standing in chemically defined medium (CDM) containing 1% Glc (wt/vol) and harvested at mid-exponential (≈ OD_600_ 0.2) or late exponential (≈ OD_600_ 0.4) phase. Specific activity of the *cps* promoter was assayed by measuring β-galactosidase activity as described before [33].

### Transcriptome analysis

For DNA microarray analysis, D39 wild type and two independently obtained D39*spxB* mutants were grown as four, two and two biological replicates, respectively, in Glc-M17 under semi-aerobic conditions and harvested at an OD_595_ of approximately 0.25 (mid-exponential). The RNA of both D39*spxB* strains was compared to D39 and the combined data was analyzed. All other procedures regarding microarray analyses were done as described before [62]. A gene was considered differentially expressed when the fold change was ≥ 2, ≤ 0.5, with a Bayes *p* ≤ 0.00001 and when at least 7 measurements were available. The microarray data is submitted to the GEO database, GSE45971.

### Growth conditions for metabolic analysis

Cells were grown at 37^°^C in static rubber-stoppered bottles (500-ml) in complex medium M17 (Difco^TM^), supplemented with Glc 1% (wt/vol) and without pH control (initial pH 6.5). Prior to inoculation sterile air was bubbled through the medium for 20 min. Alternatively cells were grown in 2-l bioreactors in the CDM described by 46 with the pH controlled at 6.5, and under initial specific air tension of 50-60% (semi-aerobic, maximum dissolved O_2_ concentration of 130 µM) or aerobic (continuous specific air tension of 40%, constant dissolved oxygen concentration of 90 µM) conditions. In the bioreactors, dissolved oxygen was monitored with a polarographic oxygen electrode (Mettler-Toledo International). The electrode was calibrated to zero or 100% by bubbling sterile argon or air through the medium, respectively. A specific air tension of 40% was maintained by automatic control of the airflow and agitation. Irrespectively of the growth conditions, Glc (1% wt/vol) was used as carbon source. Cultures were inoculated to an initial optical density at 600 nm (OD_600_) of 0.05 with cells growing exponentially in the same medium. Growth was monitored by measuring the OD_600_. Specific growth rates (μ) were calculated through linear regressions of the plots of ln(OD_600_) *versus* time during the exponential growth phase. A factor of 0.39, determined from a dry weight (DW) (mg ml^-1^) *versus* OD_600_ curve, was used to convert OD_600_ into dry weight (mg biomass ml^-1^).

### Quantification of fermentation products during growth on glucose

Strains were grown in Glc-M17 or Glc-CDM with pH control (pH 6.5) as described above. Culture samples (2 ml) were taken at different time-points of the growth curves, centrifuged (16,000 × *g*, 2 min, 4^°^C), filtered (Millex-GN 0.22 µm filters), and the supernatant solutions were stored at -20^°^C until analysis by high performance liquid chromatography (HPLC). Substrates and end-products were quantified as before [46] in an HPLC apparatus equipped with a refractive index detector (Shodex RI-101, Showa Denko K.K., Japan) using an HPX-87H anion exchange column (Bio-Rad Laboratories Inc., California, USA) at 60^°^C, with 5 mM H_2_SO_4_ as the elution fluid and a flow rate of 0.5 ml min^-1^. Alternatively, quantification of metabolites in supernatants was performed by ^1^H-NMR in a Bruker AMX300 spectrometer (Bruker BioSpin GmbH). Formic acid (sodium salt) was added to the samples and used as an internal concentration standard. The ATP yield was calculated as the ratio of ATP produced to glucose consumed. The global yields of ATP were calculated from the fermentation products determined at the time-point of growth arrest assuming that all ATP was synthesized by substrate-level phosphorylation.

### Determination of hydrogen peroxide (H_2_O_2_)

Strains were grown in Glc-CDM as described above. Culture samples of 1-ml were harvested at different time-points of the growth curves, centrifuged (16,000 × *g*, 2 min, 4^°^C) and filtered (Millex-GN 0.22 µm filters). The Amplex® Red Hydrogen Peroxide/Peroxidase assay kit (Invitrogen) was used to quantify hydrogen peroxide contents below 10 µM. Determinations of H_2_O_2_ up to 300 µM were performed as described elsewhere [63]. Briefly, 1.25 ml of peroxide reagent (192 mM phosphate, 14.8 mM azide, 0.96 ml l^-1^ Triton X-100, 2 KU l^-1^ horseradish peroxidase (Roche), 0.48 mM 4-aminophenazone and 9.6 mM chromotropic acid) was added to 50 µl of supernatant, mixed and allowed to stand for 5 minutes at room temperature. In the presence of H_2_O_2_, the chromotropic acid was converted by the peroxidase into a blue coloured compound with maximal absorbance at 600 nm. Absorbance was read at 600 nm. Water was used as the blank and standard curves were performed with fresh dilutions of a stabilized solution of 30% (wt/vol) H_2_O_2_. The absorbance of the samples was compared to that of the standard solutions.

### Determination of intracellular metabolites during growth on glucose

Strains were grown in Glc-CDM under semi-aerobic as described above. Ethanol extracts for analysis by ^31^P-NMR and quantification of phosphorylated metabolites in D39*spxB* and control strain D39 at late-exponential (LExp) and early-stationary (EStat) phases of growth (time-points indicated by the arrows in Figure 4B) were prepared as previously described by 46. In brief, a volume of culture was withdrawn from the fermenter, adjusted to obtain approximately 16-18 mg of cell protein for extract, and centrifuged (21,000 *x g*, 15 min, 4^°^C). The pellet was suspended in 28 ml of 5 mM 3-(N-morpholino) propanesulfonic acid (MOPS) buffer, pH 7.2 (Sigma-Aldrich) transferred to 70 ml of cold ethanol (final concentration 70% vol/vol) in an ice bath, and extraction was performed for 30 min with vigorous agitation. Cell debris was removed by centrifugation (35,000 *x g*, 30 min, 4°C), the ethanol was evaporated and the residue lyophilized. The dried extracts were dissolved in 1 ml ^2^H _2_O containing 5 mM EDTA (final pH approximately 6.5) and stored at -20^°^C. Assignment of resonances was based on previous studies [46] or by spiking the sample extracts with the suspected, pure compounds. The concentrations of intracellular metabolites were calculated from the areas of their resonances in ^31^P-spectra by comparison with the area of the resonance due to methylphosphonic acid (Sigma-Aldrich), added as an internal standard, and after application of an appropriate factor for correcting saturation of resonances. The reported values for intracellular phosphorylated compounds are averages of three to four independent growth experiments. The accuracy was around 15-20%. Phosphorus-31 spectra were acquired on a Bruker AVANCE II 500 spectrometer (Bruker BioSpin GmbH) with a phosphorus selective 5-mm-diameter probe head at 30°C with a pulse width of 6.5 µs (flip angle, 70°) and a recycle delay of 1s. Saturation of resonances due to fast pulsing conditions was calculated by comparison with spectra acquired under fully relaxed conditions (recycle delay, 20s). Phosphorus chemical shifts were referenced to the resonances of external 85% H_3_PO_4_ designated at 0.0 ppm.

### Capsular polysaccharide preparation and quantification of the glucuronic acid content

Samples for the determination of the capsular glucuronic acid amounts were prepared as follows: cells grown in Glc-CDM under semi-aerobic conditions as above and harvested during the late-exponential and early-stationary phases of growth (time-points indicated by the arrows in Figure 4B) were centrifuged (6,000 *x g*, 3-7 min, 4^°^C), resuspended in PBS and pelleted at 3000 *x g*, 4^°^C, for 20 min. The pellet was resuspended in 500 µl of 150 mM Tris-HCl (pH 7.0) and 1 mM MgSO_4_ and treated as described elsewhere [64]. The supernatants derived from the two centrifugations referred before were pooled and treated with 20% (wt/vol) trichloroacetic acid for protein precipitation. After 2 h of incubation in an ice bath proteins were pelleted and the exopolysaccharides were precipitated with cold ethanol as in [65]. The glucuronic acid of capsule attached or loosely attached (lost in centrifugations to the supernatants) to the cell wall was quantified by the method for quantitative determination of uronic acids as described in [66]. Capsule measurements were performed in duplicate using samples from six independent cultures.

### Growth of D39*spxB* and D39 strains on specified sugars

To test the ability of specific sugars to support growth of *S. pneumoniae* strains D39 and D39*spxB* cultures of 250 µl were prepared in CDM containing 0.25% (wt/vol) of glucose (Glc), glucuronic acid (GlcUA), rhamnose (Rha), gluconic acid (GlcA), N-acetylglucosamine (GlcNAc) or a 1:2:3 mixture of GlcUA, Glc and Rha, respectively. Cultures were started at an initial OD_595_ of 0.25-0.3 by addition of a preculture, grown on Glc-M17 (0.5% Glc wt/vol), suspended in fresh CDM without sugar. Cultures were grown for 24 h at 37°C in 96-well microtiter plates. Cell density was monitored at 595 nm with a ELx808 microplate spectrophotometer (BioTek Instruments, Inc.) and the growth curves were generated using Gen5^TM^ (BioTek Instruments, Inc.) with readings taken every 30 min. Each growth condition was done in triplicate using two independent precultures. Growth rates and other growth parameters were generated as above and the results were analyzed using the Instat software (GraphPad Software).

## Supporting Information

Figure S1 Detection of SpxB in D39, D39*spxB* and D39*spxB* ^**+**^. Detection of SpxB by Western blot using a ployclonal anti-SpxB serum. D39 (lane 1), D39*spxB* (lane 2) and D39*spxB* ^*+*^ (complemented strain) (lane 3). Strains were grown in BHI to an OD of 0.2-0.25.
(TIF)

Materials and Methods S1 Supplemental materials and methods. (PDF)

## References

[1] Obaro S, Adegbola R (2002) The pneumococcus: carriage, disease and conjugate vaccines. J Med Microbiol 51: 98-104. PubMed: 11863272. 11863272 10.1099/0022-1317-51-2-98

[2] Avery OT, Dubos R (1931) The protective action of a specific enzyme against type III pneumococcus infection in mice. J Exp Med 54: 73-89. doi:10.1084/jem.54.1.73. PubMed: 19869903. 19869903 10.1084/jem.54.1.73 PMC2132045

[3] Kadioglu A, Weiser JN, Paton JC, Andrew PW (2008) The role of *Streptococcus pneumoniae* virulence factors in host respiratory colonization and disease. Nat Rev Microbiol 6: 288-301. doi:10.1038/nrmicro1871. PubMed: 18340341. 18340341 10.1038/nrmicro1871

[4] Magee AD, Yother J (2001) Requirement for capsule in colonization by *Streptococcus pneumoniae* . Infect Immun 69: 3755-3761. doi:10.1128/IAI.69.6.3755-3761.2001. PubMed: 11349040. 11349040 10.1128/IAI.69.6.3755-3761.2001 PMC98386

[5] Hardy GG, Magee AD, Ventura CL, Caimano MJ, Yother J (2001) Essential role for cellular phosphoglucomutase in virulence of type 3 *Streptococcus pneumoniae* . Infect Immun 69: 2309-2317. doi:10.1128/IAI.69.4.2309-2317.2001. PubMed: 11254588. 11254588 10.1128/IAI.69.4.2309-2317.2001 PMC98160

[6] Song JH, Dagan R, Klugman KP, Fritzell B (2012) The relationship between pneumococcal serotypes and antibiotic resistance. Vaccine 30: 2728-2737. doi:10.1016/j.vaccine.2012.01.091. PubMed: 22330126. 22330126 10.1016/j.vaccine.2012.01.091

[7] Iannelli F, Pearce BJ, Pozzi G (1999) The type 2 capsule locus of *Streptococcus pneumoniae* . J Bacteriol 181: 2652-2654. PubMed: 10198036. 10198036 10.1128/jb.181.8.2652-2654.1999 PMC93698

[8] Hardy GG, Caimano MJ, Yother J (2000) Capsule biosynthesis and basic metabolism in *Streptococcus pneumoniae* are linked through the cellular phosphoglucomutase. J Bacteriol 182: 1854-1863. doi:10.1128/JB.182.7.1854-1863.2000. PubMed: 10714989. 10714989 10.1128/jb.182.7.1854-1863.2000 PMC101867

[9] Bentley SD, Aanensen DM, Mavroidi A, Saunders D, Rabbinowitsch E et al. (2006) Genetic analysis of the capsular biosynthetic locus from all 90 pneumococcal serotypes. PLOS Genet 2: e31. doi:10.1371/journal.pgen.0020031. PubMed: 16532061. 16532061 10.1371/journal.pgen.0020031 PMC1391919

[10] Morona JK, Miller DC, Morona R, Paton JC (2004) The effect that mutations in the conserved capsular polysaccharide biosynthesis genes *cpsA*, *cpsB*, and *cpsD* have on virulence of *Streptococcus pneumoniae* . J Infect Dis 189: 1905-1913. doi:10.1086/383352. PubMed: 15122528. 15122528 10.1086/383352

[11] Ogunniyi AD, Giammarinaro P, Paton JC (2002) The genes encoding virulence-associated proteins and the capsule of *Streptococcus pneumoniae* are upregulated and differentially expressed *in vivo* . Microbiology 148: 2045-2053. PubMed: 12101293. 12101293 10.1099/00221287-148-7-2045

[12] Hammerschmidt S, Wolff S, Hocke A, Rosseau S, Müller E et al. (2005) Illustration of pneumococcal polysaccharide capsule during adherence and invasion of epithelial cells. Infect Immun 73: 4653-4667. doi:10.1128/IAI.73.8.4653-4667.2005. PubMed: 16040978. 16040978 10.1128/IAI.73.8.4653-4667.2005 PMC1201225

[13] Kim JO, Weiser JN (1998) Association of intrastrain phase variation in quantity of capsular polysaccharide and teichoic acid with the virulence of *Streptococcus pneumoniae* . J Infect Dis 177: 368-377. doi:10.1086/514205. PubMed: 9466523. 9466523 10.1086/514205

[14] Kim JO, Romero-Steiner S, Sørensen UB, Blom J, Carvalho M et al. (1999) Relationship between cell surface carbohydrates and intrastrain variation on opsonophagocytosis of *Streptococcus pneumoniae* . Infect Immun 67: 2327-2333. PubMed: 10225891. 10225891 10.1128/iai.67.5.2327-2333.1999 PMC115974

[15] Nelson AL, Roche AM, Gould JM, Chim K, Ratner AJ et al. (2007) Capsule enhances pneumococcal colonization by limiting mucus-mediated clearance. Infect Immun 75: 83-90. doi:10.1128/IAI.01475-06. PubMed: 17088346. 17088346 10.1128/IAI.01475-06 PMC1828419

[16] Overweg K, Pericone CD, Verhoef GG, Weiser JN, Meiring HD et al. (2000) Differential protein expression in phenotypic variants of *Streptococcus pneumoniae* . Infect Immun 68: 4604-4610. doi:10.1128/IAI.68.8.4604-4610.2000. PubMed: 10899862. 10899862 10.1128/iai.68.8.4604-4610.2000 PMC98388

[17] Spellerberg B, Cundell DR, Sandros J, Pearce BJ, Idanpaan-Heikkila I et al. (1996) Pyruvate oxidase, as a determinant of virulence in *Streptococcus pneumoniae* . Mol Microbiol 19: 803-813. doi:10.1046/j.1365-2958.1996.425954.x. PubMed: 8820650. 8820650 10.1046/j.1365-2958.1996.425954.x

[18] Pericone CD, Bae D, Shchepetov M, McCool T, Weiser JN (2002) Short-sequence tandem and nontandem DNA repeats and endogenous hydrogen peroxide production contribute to genetic instability of *Streptococcus pneumoniae* . J Bacteriol 184: 4392-4399. doi:10.1128/JB.184.16.4392-4399.2002. PubMed: 12142409. 12142409 10.1128/JB.184.16.4392-4399.2002 PMC135236

[19] Pericone CD, Park S, Imlay JA, Weiser JN (2003) Factors contributing to hydrogen peroxide resistance in *Streptococcus pneumoniae* include pyruvate oxidase (SpxB) and avoidance of the toxic effects of the fenton reaction. J Bacteriol 185: 6815-6825. doi:10.1128/JB.185.23.6815-6825.2003. PubMed: 14617646. 14617646 10.1128/JB.185.23.6815-6825.2003 PMC262707

[20] Pericone CD, Overweg K, Hermans PW, Weiser JN (2000) Inhibitory and bactericidal effects of hydrogen peroxide production by *Streptococcus pneumoniae* on other inhabitants of the upper respiratory tract. Infect Immun 68: 3990-3997. doi:10.1128/IAI.68.7.3990-3997.2000. PubMed: 10858213. 10858213 10.1128/iai.68.7.3990-3997.2000 PMC101678

[21] Duane PG, Rubins JB, Weisel HR, Janoff EN (1993) Identification of hydrogen peroxide as a *Streptococcus pneumoniae* toxin for rat alveolar epithelial cells. Infect Immun 61: 4392-4397. PubMed: 8406830. 8406830 10.1128/iai.61.10.4392-4397.1993 PMC281171

[22] Hirst RA, Sikand KS, Rutman A, Mitchell TJ, Andrew PW et al. (2000) Relative roles of pneumolysin and hydrogen peroxide from *Streptococcus pneumoniae* in inhibition of ependymal ciliary beat frequency. Infect Immun 68: 1557-1562. doi:10.1128/IAI.68.3.1557-1562.2000. PubMed: 10678974. 10678974 10.1128/iai.68.3.1557-1562.2000 PMC97315

[23] Braun JS, Sublett JE, Freyer D, Mitchell TJ, Cleveland JL et al. (2002) Pneumococcal pneumolysin and H_2_O_2_ mediate brain cell apoptosis during meningitis. J Clin Invest 109: 19-27. doi:10.1172/JCI200212035. PubMed: 11781347. 11781347 10.1172/JCI12035 PMC150815

[24] Regev-Yochay G, Trzcinski K, Thompson CM, Lipsitch M, Malley R (2007) SpxB is a suicide gene of *Streptococcus pneumoniae* and confers a selective advantage in an in vivo competitive colonization model. J Bacteriol 189: 6532-6539. doi:10.1128/JB.00813-07. PubMed: 17631628. 17631628 10.1128/JB.00813-07 PMC2045178

[25] Pesakhov S, Benisty R, Sikron N, Cohen Z, Gomelsky P et al. (2007) Effect of hydrogen peroxide production and the Fenton reaction on membrane composition of *Streptococcus pneumoniae* . Biochim Biophys Acta 1768: 590-597. doi:10.1016/j.bbamem.2006.12.016. PubMed: 17292324. 17292324 10.1016/j.bbamem.2006.12.016

[26] Benisty R, Cohen AY, Feldman A, Cohen Z, Porat N (2010) Endogenous H_2_O_2_ produced by *Streptococcus pneumoniae* controls FabF activity. Biochim Biophys Acta 1801: 1098-1104. doi:10.1016/j.bbalip.2010.06.004. PubMed: 20601114. 20601114 10.1016/j.bbalip.2010.06.004

[27] Orihuela CJ, Gao G, McGee M, Yu J, Francis KP et al. (2003) Organ-specific models of *Streptococcus pneumoniae* disease. Scand J Infect Dis 35: 647-652. doi:10.1080/00365540310015854. PubMed: 14620149. 14620149 10.1080/00365540310015854

[28] Ramos-Montañez S, Tsui HC, Wayne KJ, Morris JL, Peters LE et al. (2008) Polymorphism and regulation of the *spxB* (pyruvate oxidase) virulence factor gene by a CBS-HotDog domain protein (SpxR) in serotype 2 *Streptococcus pneumoniae* . Mol Microbiol 67: 729-746. PubMed: 18179423. 18179423 10.1111/j.1365-2958.2007.06082.x

[29] Weiser JN, Bae D, Epino H, Gordon SB, Kapoor M et al. (2001) Changes in availability of oxygen accentuate differences in capsular polysaccharide expression by phenotypic variants and clinical isolates of *Streptococcus pneumoniae* . Infect Immun 69: 5430-5439. doi:10.1128/IAI.69.9.5430-5439.2001. PubMed: 11500414. 11500414 10.1128/IAI.69.9.5430-5439.2001 PMC98654

[30] Bijlsma JJ, Burghout P, Kloosterman TG, Bootsma HJ, de Jong A et al. (2007) Development of Genomic Array Footprinting for Identification of Conditionally Essential Genes in *Streptococcus pneumoniae* . Appl Environ Microbiol 73: 1514-1524. doi:10.1128/AEM.01900-06. PubMed: 17261526. 17261526 10.1128/AEM.01900-06 PMC1828782

[31] Allegrucci M, Sauer K (2008) Formation of *Streptococcus pneumoniae* non-phase-variable colony variants is due to increased mutation frequency present under biofilm growth conditions. J Bacteriol 190: 6330-6339. doi:10.1128/JB.00707-08. PubMed: 18658260. 18658260 10.1128/JB.00707-08 PMC2566003

[32] Ramos-Montañez S, Kazmierczak KM, Hentchel KL, Winkler ME (2010) Instability of *ackA* (acetate kinase) mutations and their effects on acetyl phosphate and ATP amounts in *Streptococcus pneumoniae* D39. J Bacteriol 192: 6390-6400. doi:10.1128/JB.00995-10. PubMed: 20952579. 20952579 10.1128/JB.00995-10 PMC3008541

[33] Kloosterman TG, Bijlsma JJ, Kok J, Kuipers OP (2006) To have neighbour’s fare: extending the molecular toolbox for *Streptococcus pneumoniae* . Microbiology 152: 351-359. doi:10.1099/mic.0.28521-0. PubMed: 16436423. 16436423 10.1099/mic.0.28521-0

[34] Taniai H, Iida K, Seki M, Saito M, Shiota S et al. (2008) Concerted action of lactate oxidase and pyruvate oxidase in aerobic growth of *Streptococcus pneumoniae*: role of lactate as an energy source. J Bacteriol 190: 3572-3579. doi:10.1128/JB.01882-07. PubMed: 18344365. 18344365 10.1128/JB.01882-07 PMC2395018

[35] Yum DY, Lee BY, Pan JG (1999) Identification of the *yqhE* and *yafB* genes encoding two 2, 5-diketo-D-gluconate reductases in *Escherichia coli* . Appl Environ Microbiol 65: 3341-3346. PubMed: 10427017. 10427017 10.1128/aem.65.8.3341-3346.1999 PMC91502

[36] Maruyama Y, Nakamichi Y, Itoh T, Mikami B, Hashimoto W et al. (2009) Substrate specificity of streptococcal unsaturated glucuronyl hydrolases for sulfated glycosaminoglycan. J Biol Chem 284: 18059-18069. doi:10.1074/jbc.M109.005660. PubMed: 19416976. 19416976 10.1074/jbc.M109.005660 PMC2709336

[37] Marion C, Stewart JM, Tazi MF, Burnaugh AM, Linke CM et al. (2012) *Streptococcus pneumoniae* can utilize multiple sources of hyaluronic acid for growth. Infect Immun 80: 1390-1398. doi:10.1128/IAI.05756-11. PubMed: 22311922. 22311922 10.1128/IAI.05756-11 PMC3318431

[38] Sedewitz B, Schleifer KH, Götz F (1984) Physiological role of pyruvate oxidase in the aerobic metabolism of *Lactobacillus plantarum* . J Bacteriol 160: 462-465. PubMed: 6480562. 6480562 10.1128/jb.160.1.462-465.1984 PMC214746

[39] Seki M, Iida K, Saito M, Nakayama H, Yoshida S (2004) Hydrogen peroxide production in *Streptococcus pyogenes*: involvement of lactate oxidase and coupling with aerobic utilization of lactate. J Bacteriol 186: 2046-2051. doi:10.1128/JB.186.7.2046-2051.2004. PubMed: 15028688. 15028688 10.1128/JB.186.7.2046-2051.2004 PMC374426

[40] Goffin P, Muscariello L, Lorquet F, Stukkens A, Prozzi D et al. (2006) Involvement of pyruvate oxidase activity and acetate production in the survival of *Lactobacillus plantarum* during the stationary phase of aerobic growth. Appl Environ Microbiol 72: 7933-7940. doi:10.1128/AEM.00659-06. PubMed: 17012588. 17012588 10.1128/AEM.00659-06 PMC1694206

[41] Lorquet F, Goffin P, Muscariello L, Baudry JB, Ladero V et al. (2004) Characterization and functional analysis of the *poxB* gene, which encodes pyruvate oxidase in *Lactobacillus plantarum* . J Bacteriol 186: 3749-3759. doi:10.1128/JB.186.12.3749-3759.2004. PubMed: 15175288. 15175288 10.1128/JB.186.12.3749-3759.2004 PMC419957

[42] Belanger AE, Clague MJ, Glass JI, Leblanc DJ (2004) Pyruvate oxidase is a determinant of Avery’s rough morphology. J Bacteriol 186: 8164-8171. doi:10.1128/JB.186.24.8164-8171.2004. PubMed: 15576764. 15576764 10.1128/JB.186.24.8164-8171.2004 PMC532437

[43] LeMessurier KS, Ogunniyi AD, Paton JC (2006) Differential expression of key pneumococcal virulence genes in vivo. Microbiology 152: 305-311. doi:10.1099/mic.0.28438-0. PubMed: 16436418. 16436418 10.1099/mic.0.28438-0

[44] Li-Korotky HS, Lo CY, Zeng FR, Lo D, Banks JM (2009) Interaction of phase variation, host and pressure/gas composition: Pneumococcal gene expression of PsaA, SpxB, Ply and LytA in simulated middle ear environments. Int J Pediatr Otorhinolaryngol, 73: 1417–22. PubMed: 19682756. 19682756 10.1016/j.ijporl.2009.07.007 PMC2891361

[45] Yother J (2011) Capsules of *Streptococcus pneumoniae* and other bacteria: paradigms for polysaccharide biosynthesis and regulation. Annu Rev Microbiol 65: 563-581. doi:10.1146/annurev.micro.62.081307.162944. PubMed: 21721938. 21721938 10.1146/annurev.micro.62.081307.162944

[46] Carvalho SM, Kloosterman TG, Kuipers OP, Neves AR (2011) CcpA ensures optimal metabolic fitness of *Streptococcus pneumoniae* . PLOS ONE 6: e26707. doi:10.1371/journal.pone.0026707. PubMed: 22039538. 22039538 10.1371/journal.pone.0026707 PMC3198803

[47] Zheng L, Chen Z, Itzek A, Ashby M, Kreth J (2011) Catabolite control protein A controls hydrogen peroxide production and cell death in *Streptococcus sanguinis* . J Bacteriol 193: 516-526. doi:10.1128/JB.01131-10. PubMed: 21036992. 21036992 10.1128/JB.01131-10 PMC3019840

[48] Zheng L, Itzek A, Chen Z, Kreth J (2011) Environmental influences on competitive hydrogen peroxide production in *Streptococcus gordonii* . Appl Environ Microbiol 77: 4318-4328. doi:10.1128/AEM.00309-11. PubMed: 21571883. 21571883 10.1128/AEM.00309-11 PMC3127700

[49] Lanie JA, Ng WL, Kazmierczak KM, Andrzejewski TM, Davidsen TM et al. (2007) Genome sequence of Avery’s virulent serotype 2 strain D39 of *Streptococcus pneumoniae* and comparison with that of unencapsulated laboratory strain R6. J Bacteriol 189: 38-51. doi:10.1128/JB.01148-06. PubMed: 17041037. 17041037 10.1128/JB.01148-06 PMC1797212

[50] Bidossi A, Mulas L, Decorosi F, Colomba L, Ricci S et al. (2012) A functional genomics approach to establish the complement of carbohydrate transporters in *Streptococcus pneumoniae* . PLOS ONE 7: e33320. doi:10.1371/journal.pone.0033320. PubMed: 22428019. 22428019 10.1371/journal.pone.0033320 PMC3302838

[51] Burnaugh AM, Frantz LJ, King SJ (2008) Growth of *Streptococcus pneumoniae* on human glycoconjugates is dependent upon the sequential activity of bacterial exoglycosidases. J Bacteriol 190: 221-230. doi:10.1128/JB.01251-07. PubMed: 17981977. 17981977 10.1128/JB.01251-07 PMC2223752

[52] Terra VS, Homer KA, Rao SG, Andrew PW, Yesilkaya H (2010) Characterization of novel beta-galactosidase activity that contributes to glycoprotein degradation and virulence in *Streptococcus pneumoniae* . Infect Immun 78: 348-357. doi:10.1128/IAI.00721-09. PubMed: 19841081. 19841081 10.1128/IAI.00721-09 PMC2798215

[53] Philips BJ, Meguer JX, Redman J, Baker EH (2003) Factors determining the appearance of glucose in upper and lower respiratory tract secretions. Intensive Care Med 29: 2204-2210. doi:10.1007/s00134-003-1961-2. PubMed: 14647890. 14647890 10.1007/s00134-003-1961-2

[54] Wolfe AJ (2010) Physiologically relevant small phosphodonors link metabolism to signal transduction. Curr Opin Microbiol 13: 204-209. doi:10.1016/j.mib.2010.01.002. PubMed: 20117041. 20117041 10.1016/j.mib.2010.01.002 PMC2847653

[55] Price CE, Zeyniyev A, Kuipers OP, Kok J (2011) From meadows to milk to mucosa - adaptation of Streptococcus and Lactococcus species to their nutritional environments. FEMS Microbiol Rev. PubMed: 22212109. 10.1111/j.1574-6976.2011.00323.x 22212109

[56] Neves AR, Pool WA, Kok J, Kuipers OP, Santos H (2005) Overview on sugar metabolism and its control in *Lactococcus lactis* - the input from in vivo NMR. FEMS Microbiol Rev 29: 531-554. doi:10.1016/j.fmrre.2005.04.005. PubMed: 15939503. 15939503 10.1016/j.femsre.2005.04.005

[57] Terzaghi BE, Sandine WE (1975) Improved Medium for Lactic Streptococci and Their Bacteriophages. Appl Microbiol 29: 807-813. PubMed: 16350018. 16350018 10.1128/am.29.6.807-813.1975 PMC187084

[58] Gualdi L, Hayre JK, Gerlini A, Bidossi A, Colomba L et al. (2012) Regulation of neuraminidase expression in *Streptococcus pneumoniae* . BMC Microbiol 12: 200. doi:10.1186/1471-2180-12-200. PubMed: 22963456. 22963456 10.1186/1471-2180-12-200 PMC3509027

[59] Maguin E, Prévost H, Ehrlich SD, Gruss A (1996) Efficient insertional mutagenesis in lactococci and other gram-positive bacteria. J Bacteriol 178: 931-935. PubMed: 8550537. 8550537 10.1128/jb.178.3.931-935.1996 PMC177749

[60] Karlyshev Pallen MJ, Wren BW (2000) Single Primer PCR procedure for Rapid Identification of Transposon Insertion Sites. BioTechniques 28: 1078-1082. PubMed: 10868271. 10868271 10.2144/00286bm05

[61] Iyer R, Baliga NS, Camilli A (2005) Catabolite control protein A (CcpA) contributes to virulence and regulation of sugar metabolism in *Streptococcus pneumoniae* . J Bacteriol 187: 8340-8349. doi:10.1128/JB.187.24.8340-8349.2005. PubMed: 16321938. 16321938 10.1128/JB.187.24.8340-8349.2005 PMC1317011

[62] Neef J, Andisi VF, Kim KS, Kuipers OP, Bijlsma JJ (2011) Deletion of a cation transporter promotes lysis in *Streptococcus pneumoniae* . Infect Immun 79: 2314-2323. doi:10.1128/IAI.00677-10. PubMed: 21422174. 21422174 10.1128/IAI.00677-10 PMC3125841

[63] Meiattini F (1988) Inorganic peroxides. In: Bergmeyer H Grabl M Bergmeyer J Methods of enzymatic analysis. Weinheim: Verlag Chemie pp. 566-571.

[64] Morona JK, Morona R, Paton JC (2006) Attachment of capsular polysaccharide to the cell wall of *Streptococcus pneumoniae* type 2 is required for invasive disease. Proc Natl Acad Sci U S A 103: 8505-8510. doi:10.1073/pnas.0602148103. PubMed: 16707578. 16707578 10.1073/pnas.0602148103 PMC1482522

[65] Ramos A, Boels IC, de Vos WM, Santos H (2001) Relationship between glycolysis and exopolysaccharide biosynthesis in *Lactococcus lactis* . Appl Environ Microbiol 67: 33-41. doi:10.1128/AEM.67.1.33-41.2001. PubMed: 11133425. 11133425 10.1128/AEM.67.1.33-41.2001 PMC92509

[66] Blumenkrantz N, Asboe-Hansen G (1973) New method for quantitative determination of uronic acids. Anal Biochem 54: 484-489. doi:10.1016/0003-2697(73)90377-1. PubMed: 4269305. 4269305 10.1016/0003-2697(73)90377-1

[67] Carvalho SM, Kuipers OP, Neves AR (2013) Environmental and nutritional factors that affect growth and metabolism of the pneumococcal serotype 2 strain D39 and its nonencapsulated derivative strain r6. PLOS ONE 8: e58492. doi:10.1371/journal.pone.0058492. PubMed: 23505518. 23505518 10.1371/journal.pone.0058492 PMC3591343

[68] Avery OT, MacLeod CM, McCarty M (1944) Studies on the chemical nature of the substance inducing transformation of pneumococcal types : induction of transformation by a desoxyribonucleic acid fraction isolated from pneumococcus type III. J Exp Med 79: 137-158. doi:10.1084/jem.79.2.137. PubMed: 19871359. 19871359 10.1084/jem.79.2.137 PMC2135445

[69] Tettelin H, Nelson KE, Paulsen IT, Eisen JA, Read TD et al. (2001) Complete genome sequence of a virulent isolate of *Streptococcus pneumoniae* . Science 293: 498-506. doi:10.1126/science.1061217. PubMed: 11463916. 11463916 10.1126/science.1061217

[70] Sanders JW, Venema G, Kok J, Leenhouts K (1998) Identification of a sodium chloride-regulated promoter in *Lactococcus lactis* by single-copy chromosomal fusion with a reporter gene. Mol Gen Genet 257: 681-685. doi:10.1007/s004380050697. PubMed: 9604892. 9604892 10.1007/s004380050697

