# Supplementary figures and images for: Pyruvate Oxidase Influences the Sugar Utilization Pattern and Capsule Production in Streptococcus pneumoniae
Source: PLoS One. 2013 Jul 3;8(7):e68277. doi: 10.1371/journal.pone.0068277 (PMC3701046; doi:10.1371/journal.pone.0068277)

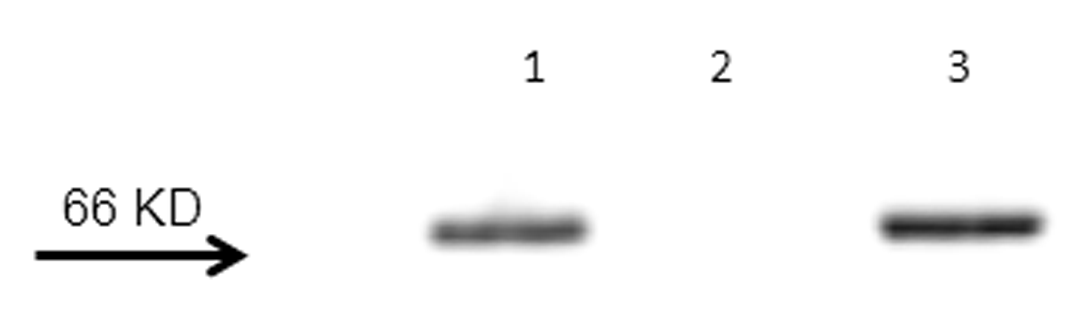

Supplement: Figure S1 — Detection of SpxB by Western blot using a ployclonal anti-SpxB serum. D39 (lane 1), D39spxB (lane 2) and D39spxB + (complemented strain) (lane 3). Strains were grown in BHI to an OD of 0.2-0.25. (TIF) [file pone.0068277.s001.tif]
