# Supplementary material for: Pyruvate Oxidase Influences the Sugar Utilization Pattern and Capsule Production in Streptococcus pneumoniae
Source: PLoS One. 2013 Jul 3;8(7):e68277. doi: 10.1371/journal.pone.0068277 (PMC3701046; doi:10.1371/journal.pone.0068277)
Supplement: Materials and Methods S1 — (PDF) [file pone.0068277.s002.pdf]

Environmental and Nutritional Factors That Affect Growth and Metabolism of the Pneumococcal Serotype 2 Strain D39 and Its Nonencapsulated Derivative Strain R6

Sandra M. Carvalho[1], Oscar P. Kuipers[2] and Ana Rute Neves[1*]

[1]Instituto de Tecnologia Química e Biológica, Universidade Nova de Lisboa, Oeiras, Portugal.

[2]Department of Molecular Genetics, Groningen Biomolecular Sciences and Biotechnology Institute, University of Groningen, Groningen, The Netherlands.

Running title: Factors that affect growth and metabolism of *S. pneumoniae*

***Corresponding author:** Ana Rute Neves
E-mail: rute@biosustain.dtu.dk
Tel. +4521126170.
Current address: Novo Nordisk Foundation Center for Biosustainability, Technical University of Denmark, Hørsholm, Denmark.

Abstract

Links between carbohydrate metabolism and virulence in *Streptococcus pneumoniae* have been recurrently established. Nevertheless, a deep understanding of basic physiology and metabolism of this human pathogen is missing. We developed a chemically defined medium (CDM) and defined growth conditions that allow a high yield of strains D39 and R6 and for application of *in vivo* NMR to study metabolism. The utilization of the defined medium enabled the evaluation of different environmental and nutritional factors on growth and fermentation patterns of strains D39 and R6 under controlled conditions of pH, temperature and gas atmosphere. Surprisingly, the same growth conditions impacted differently on D39 capsulated, and its isogenic nonencapsulated derivative R6. A semi-aerobic atmosphere and a raised concentration of uracil, a fundamental component of the D39 capsule, improved considerably D39 growth rate and biomass. In contrast, in strain R6, the growth rate was enhanced by strictly anaerobic conditions and uracil had no effect on biomass. In the presence of oxygen, the difference in the growth rates was mainly attributed to a lower activity of pyruvate oxidase in strain D39. Our data indicate an intricate connection between capsule production in strain D39 and uracil availability. In this study, we have also successfully applied the *in vivo* NMR technique to study sugar metabolism in *S. pneumoniae* R6. Glucose consumption, end-products formation and evolution of intracellular metabolite pools were monitored online by ^{13}C-NMR. Additionally, the pools of NTP and inorganic phosphate were followed by ^{31}P-NMR after a pulse of glucose. These results represent the first metabolic profiling data obtained non-invasively for *S. pneumoniae*, and pave the way to a better understanding of regulation of central metabolism.

Introduction

Streptococcus pneumoniae is a commensal organism of the human nasopharynx, and an opportunistic bacterium that can cause a number of serious diseases such as pneumonia, meningitis and septicaemia (reviewed in [1]). According to the World Health Organization (WHO), diseases caused by *S. pneumoniae* constitute a major global public health problem, leading to an estimated 1 million deaths per year in children under the age of five (http://www.who.int/nuvi/pneumococcus/en/). This high mortality is exacerbated by the rate at which the organism acquires resistance to traditional antibiotics. Therefore, it is without surprise that the study of *S. pneumoniae* pathogenesis has focused on factors influencing host-pathogen interactions, such as toxins, cell wall components and capsule (reviewed in [2]). However, factors that enable persistence and proliferation of the pneumococcus in different host niches, as well as the mechanisms coordinating expression of genes required to adapt to different niches have largely been neglected. As a strictly fermentative bacterium, carbohydrates are most likely the only nutrients from which the pneumococcus can obtain sufficient energy to support growth. This view is strengthened by the large portion of the pneumococcal genome that is devoted to carbohydrate uptake and metabolism [3-5]. It is, therefore, not surprising that genes involved in central metabolic processes, namely carbohydrate transport and utilization, recurrently appear in genome-wide studies aimed at identifying genes essential for virulence [6]. Growing evidence adds to these findings by showing that carbohydrate transport systems, metabolic enzymes and a global regulator of carbon metabolism (CcpA) directly contribute to *S. pneumoniae* colonization and disease [7-15]. These studies establish a link between regulation of virulence and carbohydrate metabolism, denoting a far greater importance of basic metabolic physiology than previously imagined. Recently, it was recognized that a true understanding of metabolism is perhaps more difficult to attain than that of any other cellular system [16], because metabolism is influenced by a vast number of regulatory activities at different cellular levels, and metabolism itself feeds back to all the other cellular processes, including metabolic networks.

In accordance, lack of correlation between metabolic behaviors and changes in transcript levels [17-19], emphasize the importance of examining metabolic operation in detail. Capturing the essence of complex regulatory mechanisms as those involved in carbohydrate metabolism demands the use of well-defined physiological conditions.

A powerful technique for studying metabolism in a non-invasive way is *in vivo* NMR spectroscopy. This methodology provides real time information on the pools of intracellular metabolites and metabolic fluxes and can also be used to identify metabolic bottlenecks and regulatory sites (reviewed in [20]). The application of NMR to study metabolism is largely facilitated by the use of a proper chemically defined medium (CDM) for growth [21]. In CDM all the components and respective concentrations are defined, facilitating data interpretation and improving reproducibility between experiments [22,23]. CDM formulations for *S. pneumoniae* are available, but the maximal pneumococcal biomass formed in these media is generally below an optical density value of 1 [24-26]. Low biomass yields are inadequate when *in vivo* NMR is to be used for metabolic studies, as this technique requires the utilization of dense cell suspensions. Furthermore, paramagnetic ions (*e.g.* Mn^{2+}, Fe^{2+}), which are well known for lowering the sensitivity of NMR spectroscopy (reviewed in [20]), are generally present at relatively high concentrations in the defined media for the pneumococcus.

Most cultivation optimizations for streptococcal growth have been performed in complex media with the goal of producing capsular polysaccharide on a large-scale for industrial application (*e.g.* manufactured vaccines) [27,28]. To our knowledge, there is no specific data on growth and cultivation conditions in CDM supporting high biomass production of the laboratory model *S. pneumoniae* serotype 2 strain D39 and its acapsular derivative R6 [4,5,24,29]. Curiously, and despite the historical significance of these two strains, a thorough comparative metabolic characterization is missing. In this work, we optimized a CDM and growth conditions that support high yields of strains D39 and R6. Growth and fermentation profiles in the improved CDM were obtained for both strains under controlled conditions of pH, gas atmosphere and temperature. The effect of oxygen, glucose

and nucleobases on the growth physiology of the two strains was assessed. Dissimilarities in growth profiles were tentatively interpreted on the basis of the reported differences in the genetic content of strains D39 and R6 [5]. Finally, the optimized cultivation conditions achieved for strain R6 were proven suitable for *in vivo* NMR experiments. The metabolism of glucose was studied non-invasively in real time. Time series for the consumption of glucose, end-products formation and accumulation of the glycolytic intermediate fructose 1,6-bisphosphate (FBP) were obtained by ^{13}C-NMR with a time resolution of 30 s, while the pools of NTP and inorganic phosphate were investigated by ^{31}P-NMR. The application of this technique to *S. pneumoniae* is expected to extend our knowledge on the intricate metabolic operation of this human pathogen.

Results and Discussion

Chemically defined medium for high yield streptococcal growth

The study of metabolic complexity is largely facilitated by the application of well defined environmental and nutritional conditions. In this respect, bacterial growth in media for which the exact component concentrations are known (chemically defined media) is preferred. A number of CDM formulations have been described before for *S. pneumoniae* [24-26]. However, these CDM were not suitable to obtain enough biomass for *in vivo* NMR experiments due to the presence of high concentrations of paramagnetic ions and to the low biomass yields obtained during growth. Paramagnetic ions broaden the line width of NMR spectra, decreasing significantly the spectral quality and hampering detection of intracellular metabolites. Thus, we sought for a CDM formulation devoid of paramagnetic ions that could be used routinely in the laboratory to grow *S. pneumoniae* with a concomitant high biomass production. A CDM enabling the acquisition of high quality NMR data has been described for the closely related bacterium *Lactococcus lactis* [21]. We used the lactococcal medium as starting point to develop a CDM intended for all physiological studies performed in our laboratory. However, a simplification and optimization of the medium was not carried out,

since our aim was not to design a minimal medium containing only essential nutrients. Conversely, our goal was to obtain a CDM for high yield streptococcal growth. Thus, initial growth tests, in which the lactococcal CDM was supplemented with additional nutrients, were performed with *S. pneumoniae* strain R6 in standing rubber-stoppered bottles using 60 mM of glucose as carbon source. Choline-HCl and pyruvate (Table 1) are chemicals generally used in CDM for *S. pneumoniae* growth [24-26]. The growth dependency of *S. pneumoniae* on exogenous choline, which is used to decorate its unusual teichoic acids, is well known [30], and was confirmed in our conditions (data not shown). Furthermore, we verified that increasing the choline-HCl concentration from 5 to 10 mg l^{-1} increased the final biomass by 30%. Sodium pyruvate (0.1 g l^{-1}, Table 1) is an ingredient of our CDM, but a 10-fold concentration reduction or even its omission had no effect on biomass or growth rate of strains (data not shown). Possibly, the acetate present in our CDM (Table 1) masks the effect arising from pyruvate depletion [31]. Moreover, individual addition of $CuSO_4$, $FeCl_3$, $CoCl_2$, or $MnSO_4$ to a final concentration of 0.05 mg l^{-1} in the medium had no effect on the growth profile of *S. pneumoniae* R6 (data not shown). Also, supplementation with tyrosine (0.025 g l^{-1}) or inositol (0.5 mg l^{-1}) showed no effect, while ascorbate (0.5 g l^{-1}) affected the growth negatively and complex nitrogenous sources (soytone and casein EH) improved the final biomass by about 50%. The latter result might indicate a preference of *S. pneumoniae* for oligopeptides over single amino acids. Oligopeptide transporters have been identified in *S. pneumoniae* [4,32]. The undefined composition of the complex nitrogenous sources hinders, however, their inclusion in our CDM. Thus, the final composition of the medium was set as in Table 1, and typical growth curves for precultures and cultures obtained under semi-aerobic conditions (standing rubber-stoppered bottles, cultivation conditions as in Materials and Methods) for strains R6 and D39 are shown in Fig. 1.

Batch cultivations under controlled environmental conditions

S. pneumoniae is a strictly fermentative organism that relies on the energy obtained during the conversion of sugars into pyruvate for growth. To fulfill the redox balance, the NAD^+ consumed in glycolysis is primarily recycled through reduction of pyruvate to lactate, causing acidification of the medium and ultimately growth arrest. It is well established that increasing acidities progressively inhibit growth of *Streptococcaceae* [33,34]. Thus, we hypothesized that biomass production could be improved by maintaining the medium pH at 6.5, a value determined in independent experiments to be beneficial for growth of *S. pneumoniae* (data not shown). To circumvent medium acidification we resorted to high performance bioreactors to perform batch cultivations of *S. pneumoniae*. Bioreactors allow for the tight control of pH, as well as other growth parameters, such as gas atmosphere and temperature, and provide increased working volumes. Hence, strain R6 was grown in a 2-l fermentor vessel under controlled conditions of pH (6.5), temperature (37ºC) and gas atmosphere (anaerobiosis), and the effects of pH control and preculture age were examined in CDM supplemented with 60 mM glucose.

(i) Effect of pH control and preculture age on growth of *S. pneumoniae* R6. Preliminary results pinpointed the age of the starting preculture as a determinant factor in the physiology of growth. This observation was confirmed in batch cultivations in which all other parameters were kept constant, except for the age of the preculture used to initiate growth (Fig. 2 and Table 2). Indeed, both the growth rate (μ, h^{-1}) and the maximal cell mass (OD_{Max}) were considerably higher when late-exponentially phase precultures (6-7 hours at 37ºC) rather than early-stationary precultures (8-9 hours at 37ºC) were used for inoculation. Moreover, no growth was detected during the 14 h of monitoring when precultures in late-stationary phase (18 h) were used for inoculation. Although a full explanation cannot be put forward, it is likely that with ageing larger fractions of the cell population switch to the lytic phase and are thereby unable to divide [35-37]. Cell viability was not determined under these conditions, but a repeatedly observed decrease in the maximal OD_{600} after 10-11 hours of

incubation at 37ºC is indicative of cell death/lysis. The initial growth retardation can be understood on this basis, but the reason as to why the maximal biomass is much lower remains unclear. It is well documented that both the metabolic status and gene expression levels are dramatically affected in cells entering stationary phase [37-39], and consequently the observed dissimilar behaviors can lie in complex mechanisms, the study of which is out of the scope of this work. Considering the results, the utilization of exponentially harvested *S. pneumoniae* cells to initiate cultures was applied and is routinely used in our laboratory. Implementation of this procedure circumvented an array of growth defects, such as long lag phases, inability to enter exponential phase or low biomass production.

As for pH control, in accordance to our hypothesis R6 cultures grown at constant pH of 6.5, under semi-aerobic conditions (Fig. 3A), showed a 2-fold increase in maximal biomass (OD_{Max} 3.6 as compared to 1.7-1.9 in cultures without pH control) and identical growth rate (Table 3 and Fig. 1A). Thus, pH is a key parameter to control when high biomass yields are to be obtained.

(ii) Growth and fermentation profiles of *S. pneumoniae* D39 at constant pH of 6.5. pH control was shown to positively affect the performance in terms of OD_{Max} of the unencapsulated D39-derivative strain R6 (Fig. 3A, Fig. 1A and Table 3). Considering the genomic relatedness of strain R6 to its progenitor strain D39 [5], we expected the latter to perform similarly when grown under the same conditions (CDM with 60 mM glucose, semi-aerobiosis, pH 6.5, and 37ºC). To our surprise, strain D39 reached an OD_{Max} value of 1.3 ± 0.1, slightly lower than that under non-controlled pH conditions (1.6 ± 0.2), and reduced by 64% when compared to OD_{Max} value of strain R6 (3.6 ± 0.2). The growth rate was independent of pH control, and higher than that of strain R6 by 15%. Intrigued by these results we questioned whether the dissimilar growth profiles could be due to an altered central metabolism. Accordingly, we examined substrate consumption and the pattern of end-products resulting from the fermentation of glucose (for a metabolic scheme see Fig. 4). Under controlled pH conditions, strain R6 stopped growing due to glucose limitation (glucose was totally consumed in the early-stationary phase after 9 h of growth, Fig. 3A and Table 4).

A similar behavior had previously been reported for *L. lactis* grown under constant pH in the CDM improved for NMR [21], and is common among the *Streptococcaceae*. In contrast, growth arrest in D39 cultures occurred at time-point 5 h while glucose was still abundant in the medium (Fig. 3A and Table 4), suggesting that a factor (nutritional or environmental) other than the carbon substrate is limiting growth. Both strains showed, however, typical homolactic fermentation, lactate being by far the major end-product. Minor amounts of mixed-acid fermentation products were also detected in supernatants of both strains, but pyruvate accumulation was only observed for strain D39 (Table 4). To our knowledge, in *S. pneumoniae* three competing enzymes, lactate dehydrogenase, pyruvate formate-lyase and pyruvate oxidase, might catalyze the conversion of pyruvate to end-products (Fig. 4). The pyruvate dehydrogenase complex has been postulated (grey lines in scheme portrayed in Fig. 4), but its occurrence remains to be proved [31,40]. The distribution of end-products is thereby dependent on the specific activities and the affinities of each enzyme for pyruvate as well as the presence or absence of oxygen (Fig. 4). Production of formate (3.4 ± 0.4 mM), ethanol (1.3 ± 0.3 mM) and acetate (2.2 ± 0.5 mM) in strain R6 is consistent with pyruvate formate-lyase activity, since formate is produced in an equimolar amount to the sum of acetate and ethanol [12]. Formate (1.1 ± 0.1 mM) was also detected in the culture medium of strain D39, but ethanol and acetate were most likely below the detection limit (0.4 - 0.5 mM) of the HPLC technique. Considering this end-product distribution, the mixed-acid products accounted only for 3% of the glucose used in each strain. Accumulation of pyruvate in the medium suggests impairment of the activities at the pyruvate node in strain D39. In view of the fermentation type exhibited by *S. pneumoniae*, the most obvious candidate to be affected is lactate dehydrogenase (LDH). However, the activity values determined for LDH in cell extracts of strains D39 and R6 (Table 5) were in the same range, indicating a similar expression of LDH in both strains. The *in vivo* activity could, however, be directly influenced by metabolic regulation. In view of the higher glucose consumption rate of strain D39 relative to strain R6, it is reasonable to hypothesize dissimilar accumulation of intracellular metabolites, potentially involved in metabolic regulation (Table 4). Considering that LDH

activity does not seem to be in great excess, since a lactate flux of 1.5 µmol min^{-1} mg^{-1} prot. can be estimated from a maximal glucose consumption rate of 0.92 µmol min^{-1} mg^{-1} prot. and a lactate yield of 1.63, direct modulation could easily explain the pyruvate accumulation. To the best of our knowledge a thorough biochemical characterization of the pneumococcal LDH is not available and, hence, potential activity inhibitors and/or activators unknown.

The gene encoding pyruvate oxidase, *spxB*, is among the 81 allelic variants in strain R6 and D39 [5]. A major consequence of this genetic variation is the different pyruvate oxidase activity values reported in the literature for D39 and R6 strains [41,42], and fully corroborated by our own activity measurements in fresh lysates of cells grown aerobically (Table 5). Furthermore, the detection of H_2O_2 in the cultivation medium of strain D39 grown semi-aerobically is indicative of *in vivo* activity under the conditions studied. Thus, the lower pyruvate oxidase activity of strain D39 could in part explain the higher accumulation of pyruvate in the growth medium. In addition, this metabolic trait is likely reinforced in strain D39 by the higher NADH oxidase activity (Table 5), which overcomes the need to regenerate NAD^+ through pyruvate reduction (Fig. 4). However, these mechanisms cannot account for the total pyruvate accumulation (0.78 ± 0.08 mM), mainly because under semi-aerobic conditions the oxidase activities are limited by the oxygen in the medium (initial concentration of *circa* 0.13-0.14 mM), which decreased to undetectable levels in about 60 min for strain D39 (Fig. S1). The rate of oxygen consumption was lower in strain D39 than in R6 (Fig. S1), which is consistent with the lower pyruvate oxidase activity, but not the higher NADH oxidase activity. Considering that the specific pyruvate oxidase activity is lower than that of NADH oxidase, the latter results can only be explained assuming a higher affinity of the pyruvate oxidase for oxygen.

The carbon and redox recoveries were consistently lower for strain D39 (Table 4). Based on this observation it is tempting to speculate that in the capsulated strain carbon is being re-directed from merely catabolic processes to biosynthesis.

Effect of oxygen on growth and metabolism of *S. pneumoniae* D39 and R6

Our data under semi-aerobic conditions establishes substantial differences in the growth profiles of strains D39 and R6. The level of oxygenation influences a number of cellular processes in *S. pneumoniae*, including central metabolism and competence [40,43]. Thus, we deemed important to examine the effect of oxygen availability on the growth of strains D39 and R6.

(i) Growth and fermentation profiles of *S. pneumoniae* D39 and R6 under anaerobic conditions (argon atmosphere). The growth profiles of strains D39 and R6 under strictly anaerobic conditions are depicted in Fig. 3B. The values of OD_{Max} obtained, 1.4 ± 0.2 and 3.7 ± 0.0 for strains D39 and R6, respectively, were similar to those observed in semi-aerobic conditions (Table 3). Under anoxic conditions, while the growth rate of strain D39 was decreased by 30%, that of strain R6 was slightly higher as compared to semi-aerobic conditions (Table 3). Likewise under semi-aerobiosis, glucose was still abundant when growth of strain D39 ceased (66% of the initial glucose remained in the culture medium at the time-point of maximal biomass, 8h, Table 4), whereas strain R6 consumed all the glucose present in the medium (time-point 7h, Table 4). The distribution of end-products was comparable to that observed under semi-aerobic conditions, except for the accumulation of pyruvate, lactate and formate in strain D39. In D39, at the time-point of growth arrest (maximal biomass, 8h), pyruvate, lactate and formate reached concentrations of 0.12 ± 0.01 mM, 38 ± 6 mM and 1.5 ± 0.1 mM, respectively. The concentrations of these end-products under semi-aerobic conditions at the time-point of growth arrest were 0.78 ± 0.08 mM, 28 ± 0 mM and 1.1 ± 0.2 mM, respectively. Considering that pyruvate reduction under O_2 nil conditions is only dependent on the activities of LDH and PFL (Fig. 4), most likely the lower accumulation of pyruvate results from the higher pressure to regenerate NAD^+ via the dehydrogenases downstream of pyruvate. In strain R6, lactate reached a concentration of 107 ± 6 mM, accounting for 89% of the glucose consumed and formate (3.7 ± 0.7 mM), acetate (2.8 ± 0.5 mM) and ethanol (0.86 ± 0.16 mM) were produced at a 2:1:1 ratio,

denoting activity of PFL. Assuming that all ATP is formed by substrate level phosphorylation the higher ATP yield (mol ATP mol^{-1} glucose) in strain R6 (Table 4) is consistent with higher acetate production.

Considering the fermentation profiles exhibited by strains D39 and R6, the differences in the growth rates between semi-aerobic and anaerobic conditions can tentatively be explained on the basis of pneumococcal metabolic activities. The 30% decline in D39´s growth rate when switching the atmosphere from semi-aerobiosis to anaerobiosis can partly be due to the decreased glucose consumption rate (0.92 ± 0.04 in semi-aerobiosis as compared to 0.60 ± 0.16 µmol min^{-1} mg^{-1} prot. in anaerobiosis) (Table 4), which in turn can be attributed to a lower NAD^+ recycling capacity under anoxic conditions, as NADH oxidases are inoperative. Under semi-aerobic conditions, while oxygen is available (60 min), activity of NADH oxidase presumably enables a faster NAD^+ regeneration, and consequently a higher glucose consumption rate (Table 4). It is worth noting that the activity of NADH oxidase measured in cell extracts of strains R6 and D39 (Table 5) was considerably higher than that found for other related bacteria [44,45]. The lower growth rate of R6 under semi-aerobic conditions can be a direct consequence of the activity of pyruvate oxidase (Table 5). The involvement of its product, H_2O_2, in oxidative stress and genetic instability in *S. pneumoniae* is documented [46,47].

(ii) Growth and fermentation profiles of *S. pneumoniae* D39 in aerobic conditions (continuous supply of 40% air). We then examined the effect of supplying a constant oxygen tension on the growth of strains D39 and R6. Under aerobic conditions the maximal biomass (OD_{Max}) reached for strains D39 (0.49 ± 0.03) and R6 (0.23 ± 0.02) were, respectively, 3-fold and 16-fold lower than those observed under semi-aerobic conditions (Fig. 3C and Table 3). This drastic decrease in the OD_{Max} contrasts with the higher growth rates obtained for both strains (around 1 h^{-1}) (Table 3). Noteworthy, at the time-point of transition to stationary phase of growth (time-point 2h), strains D39 and R6 consumed only 3.7 % and 2.8 % of the glucose supplied, respectively (Fig. 5 and Table 6). In both strains, the presence of oxygen (at a concentration of 0.09 mM) shifted the metabolism from lactate

to acetate and H_2O_2 production, denoting a high activity of pyruvate oxidase (SpxB) (Fig. 4 and Fig. 5). In strain R6 this shift was more pronounced, as indicated by the higher yields of acetate and H_2O_2 (Table 6). This observation is in good agreement with the higher activity of pyruvate oxidase measured for this strain and, possibly, with lower activity of the competing enzyme, NADH oxidase (Table 5 and Fig. 4). In contrast to work by others, in our study, a considerable reduction of NADH oxidase activity was observed in cells grown aerobically relative to semi-aerobically grown cells (Table 5) [48,49]. Under these aeration conditions, formate was not detected in the culture medium, indicating total inhibition of PFL (Fig. 5 and Table 6). Interestingly, growth arrest was observed when the levels of acetate and H_2O_2 in the medium reached the values of about 2 and 1 mM, respectively, independently of the strain (Fig. 5). After the transition to stationary growth phase (time-point 2h), no glucose was significantly consumed (Fig. 5 and Table 6) and the levels of acetate and H_2O_2 increased at the expense of lactate, showing lactate oxidase (LOX coded by *lcto*) activity (Fig. 4 and Fig. 5). In accordance, in the early-stationary phase of growth (time-point 3h) the yields of lactate decreased and the yields of acetate and H_2O_2 increased (Table 6). *S. pneumoniae* strain GTC13809, when grown aerobically, displayed similar metabolic features [40]. This, however, is not a general mechanism among lactic acid bacteria [45,50,51]. Most commonly, activity of lactate and/or pyruvate oxidase is only apparent after glucose depletion, a phenomenon that allows additional metabolism after glucose starvation [50,51]. The advantage of pyruvate recycling via lactate oxidase is extra generation of ATP in the ensuing conversion to acetate (Fig. 4). Therefore, in accordance with higher acetate production, the higher ATP yields (mol ATP mol^{-1} glucose) determined for strains D39 and R6 under aerobic conditions were expected (Table 6).

In aerobic conditions, the surplus of ATP produced by *S. pneumoniae* D39 and R6 strains most likely enhanced their growth rates, leading to increased biomass yields (g mol^{-1} glucose), but did not impact on the maximal biomass (OD_{Max}) achieved (Table 3 and Table 6). This observation suggests a re-direction of ATP away from biosynthetic purposes into the maintenance of cellular processes, an event that can be triggered by a stressful condition,

like high accumulation of H_2O_2. Considering the excess of nutrients and substrate present in the medium at the time-point of growth arrest and the control of pH to 6.5, the most plausible candidate affecting biomass production is H_2O_2. In accordance, studies have shown induction of pneumococcal death by H_2O_2 [37,42]. Moreover, the concentration of H_2O_2 accumulated at the time-point of growth arrest (Fig. 5), about 1 mM, is in good agreements with the reported minimal inhibitory concentration (MIC) necessary to prevent growth of strain D39 [46]. Therefore, the higher maximal biomass achieved by strain D39 can be explained by the 3-fold lower H_2O_2 to biomass ratio in this strain as compared to R6 (compare 5.7 ± 0.1 in D39 to 15.5 ± 0.1 nmol H_2O_2 mg DW^{-1} in R6 at time-point 2h). A major drawback in the aerobic metabolism of sugars by *S. pneumoniae* is its poor capacity to break down H_2O_2. Indeed, the pneumococcus does not possess the typical defence mechanisms against oxidative stress, such as catalase activity or expression of homologues of the OxyR/PerR transcriptional regulators [3-5]. However, production of H_2O_2 in the mM range is also a competitive advantage used by *S. pneumoniae* to kill or inhibit other potential nasopharyngeal flora members, including *H. influenzae* and *N. meningitides* [46,47]. In this context, metabolic activities downstream of pyruvate in *S. pneumoniae* could have arisen from an evolutionary adaptation to the oxygen-rich environment of the nasopharynx, which is abundantly populated by other competitor microorganisms.

Manipulation of the concentration of nutrients in culture medium

Our data show that both growth and metabolic profiles of strains D39 and R6 are differently affected by oxygen. Semi-aerobiosis (50-60% initial air tension) supported the highest growth parameters (growth rate and biomass) in strain D39. Therefore, this condition was chosen for further studies

Recently Hathaway and co-workers showed that capsule is a cost in energetic terms and probably competes for energy with the other metabolic processes [19]. We show that growth arrest of strain D39 occurs well before glucose depletion, independently of the

parameters tested. Conversely, total consumption of glucose was detected for strain R6 under anaerobic and semi-aerobic conditions. Considering that capsule is a major difference between the two strains, it is plausible to assume that its production is an additional cost in nutritional terms at the expense of biomass (Fig. 4). This hypothesis was investigated by varying the amounts of medium components presumably required for capsule synthesis. In *S. pneumoniae* strain D39, serotype 2 capsule is a major virulence factor formed by repeating units of glucose, glucuronic acid and rhamnose in the proportion of 1:2:3 [52]. The precursors of these sugar monomers (UDP-glucose, UDP-glucuronic acid and dTDP-rhamnose) require UTP and dTTP for their synthesis [52]. The sugar moiety in the NDP-sugars derives from the glycolytic intermediate glucose 6-phosphate (Fig. 4). Hence, in addition to ATP generation, glucose is also used for capsule biosynthesis. In our conditions, glucose (~1% wt/vol) is apparently in excess in D39 cultivations, and thereby the nucleobases are the most promising candidates as growth-limiting nutrients. Thus, the effect of varying the nucleobases in the culture medium was assessed. To completely rule out glucose as the limiting nutrient, fermentations at a lower and a higher initial glucose concentration were also performed.

(i) Effect of glucose concentration on growth of *S. pneumoniae* D39 and R6. Fig. 6 shows the growth profiles of strains D39 and R6 in the presence of different glucose concentrations. The growth profile of strain D39 was not significantly changed when 0.5% rather than 1% (wt/vol) glucose was used as carbon source (Fig. 6A). The growth parameters, growth rate (0.80 ± 0.06) and OD_{max} (1.5 ± 0.0), were identical to those observed on 1% glucose (Table 7). This behavior was not unexpected considering that D39 had consumed only 28% of the glucose in medium containing 1% (wt/vol) of the sugar. On the other hand, strain R6 showed a decrease in maximal biomass of about 40% when grown on 0.5% as compared to 1% glucose (Fig. 6B and Table 7). Interestingly, triplicating the glucose in the culture medium led to an initial 2-fold decrease in the growth rate of strain D39 (from time-point 0 to time-point 2h, Fig. 6A), when comparing with the growth on 1% glucose, and a lag of 1h in strain R6 (Fig. 6B). However, after the time-point 2h, strain D39 recovered to

88% of the growth rate on 1% glucose. The growth rate of strain R6 on 3% glucose was 1.5-fold lower than that on 1% glucose. Unexpectedly, the maximal biomass achieved by strain R6 on 3% glucose was slightly lower than that on 1% glucose (Fig. 6B and Table 7). In the dairy *L. lactis*, a positive correlation between biomass and sugar concentration has been reported up to concentrations of 2.5% (wt/vol); at an external concentration of 5%, a slight decrease (about 10%) in maximal biomass was observed [53]. Thus, *S. pneumoniae* seems to be more sensitive to high glucose concentrations than the dairy *L. lactis*. A complete explanation for the different behaviors is difficult to put forward, but it might be related to the environments sensed by these microorganisms in their natural habitats: while the dairy *L. lactis* is continuously exposed to a high sugar concentration in milk (~150 mM lactose), *S. pneumoniae* thrives in the nasopharynx where free sugars, and in particular glucose (< 1 mM), are low [6]. Lipid bilayers are impermeable to glucose, thus the inhibitory effect of glucose (3% wt/vol) most likely occurs in the cellular membrane. Since the growth of strains D39 and R6 was not limited or inhibited by 1% (wt/vol) of glucose, this concentration was used in subsequent studies. Furthermore, our data clearly shows that glucose is not the limiting-nutrient in pH-controlled batch cultures of strain D39.

(ii) Effect of nucleobases concentration on growth of *S. pneumoniae* D39 and R6. The nucleobases present in our CDM (Table 1) are the purines adenine, guanine and xanthine and the pyrimidine uracil. To investigate the effect of this group of nucleobases on the growth of strains D39 and R6, their concentrations were raised simultaneously from 10 to 30 mg l^{-1} in the culture medium. This increase had a marked positive effect on the maximal biomass reached by strain D39 but did not improve growth of strain R6 (Fig. 7). The OD_{Max} reached by strain D39 in CDM containing 30 mg l^{-1} of nucleobases was 3.2 ± 0.0, a value 2.5-fold higher than that obtained in CDM with 10 mg l^{-1} of nucleobases (1.3 ± 0.1) (Fig. 7A). The growth rate and the growth profile of strains D39 and R6, respectively, were not affected by increasing the concentration of nucleobases (Fig. 7). The data indicate that capsule biosynthesis demands a group of nucleobases or a particular nucleobase. In line, dissimilar behaviors were also expected between strains D39 and R6 grown in CDM without

nucleobases. Growth of strain D39 in the absence of nucleobases was characterized by a long lag-phase and an OD_{Max} of about 3 at time-point 27h (data not shown). On the other hand, strain R6 exhibited no lag-phase, a growth rate of about 0.54 h^{-1}, and an OD_{Max} of approximately 2.3, in CDM without nucleobases (data not shown). Our data indicate that strains D39 and R6 are able to synthesize nucleobases. The presence of genes encoding their biosynthetic pathways in the genome sequences of strains R6 and D39 fully corroborates our results [4,5]. Furthermore, the maximal biomass obtained for strain D39 grown without nucleobases (OD_{Max} 3), was 2.3-fold higher than that achieved by the same strain on 10 mg l^{-1} of nucleobases (OD_{Max} 1.3), implying that the pathways for their synthesis were repressed when the nucleobases were present in the medium. Thus, growth was limited by concentration.

(iii) Effect of uracil on growth and glucose fermentation in *S. pneumoniae* D39 and R6. Given the results above, we deemed important to determine if the positive effect of the nucleobases on growth of strain D39 was due to a particular base or the ensemble. Of the four nucleobases present in our CDM (Table 1), uracil is a constituent of UDP-glucose and UDP-glucuronic acid, precursors of D39 serotype 2 capsule repeating units (Fig. 4) [52]. Transporters for uracil are predicted in the genome sequence of strain D39 [5]. Thus, we hypothesized uracil to be the limiting nutrient. Thymine nucleosides are formed from uracil nucleosides in the salvage pathway of pyrimidine biosynthesis, (reviewed in [54]), and to our knowledge transporters for thymine in *Streptococcaceae* have not been described (reviewed in [54]). To test our hypothesis, strain D39 was grown in microtiter plates in CDM containing 0.25% (wt/vol) glucose (initial pH 6.5), and each nucleobase was added individually to a final concentration of 30 mg l^{-1}. Among the nucleobases tested, only an increase in uracil concentration led to improved growth of strain D39 (Fig. S2). Therefore, the effect of uracil *per se* was investigated. D39 was grown in static rubber-stoppered bottles in CDM containing 1% (wt/vol) glucose, without pH control (initial pH 6.5), and uracil was added to final concentrations of 40, 30, 10, 5, 3.3, 1, 0.67 and 0 mg l^{-1}. Interestingly, incrementing the final concentration of uracil in the culture medium from 0.67 mg l^{-1} to 30 mg l^{-1}, led to an increase

in maximal biomass from about 0.2 to 2.2 of OD_{Max} (Fig. 8A). Notably, this increase was linear from 0.67 mg l^{-1} to 10 mg l^{-1} uracil (Fig. 8B). The maximum growth rate (approximately 0.8 h^{-1}) was not significantly affected, except for the lower concentrations of 3.3, 1 and 0.67 mg l^{-1}, which exhibited a rate of about 0.74, 0.53 and 0.48 h^{-1}, respectively. A saturation at around 30 mg l^{-1} of uracil was found, as increasing the concentration even further, *i.e.* to 40 mg l^{-1}, did not improve growth (Fig. 8). Strain D39 was able to grow in uracil-free medium, as expected from genome analysis [5], and the growth profile displayed a lag of 6h, a growth rate of 0.42 h^{-1} and 1.8 of $OD_{Max.}$ In lactic acid bacteria, pyrimidine nucleobases are synthesized *de novo* using bicarbonate (HCO_3^-) or CO_2 and amino acids as substrates (reviewed in [54]).

In pH-controlled batch cultivations, a 3-fold increase in the concentration of uracil had a similar effect on growth (μ, 0.80 ± 0.02 h^{-1} and OD_{Max} of 3.2 ± 0.1) as that produced when all the four nucleobases were 3 times increased (Fig. 7A). This result unequivocally shows that uracil is the sole base stimulating biomass production in the capsulated strain D39. Indeed, the increment of uracil concentration did not affect the growth rate of strain R6. Curiously, strain D39 showed a more pronounced shift to mixed-acid fermentation in glucose-CDM containing 30 mg l^{-1} uracil (Fig. 9). Lactate was the major end-product, accounting for 76% of the glucose consumed (Table 8), and formate (6.3 ± 1.3 mM) was produced in a ratio of 2:1:1 relative to ethanol (2.8 ± 1.1 mM) and acetate (3.1 ± 0.4 mM), which is ascribed to PFL activity. The pyruvate accumulated in this condition (*circa* 0.44 mM) was 2-fold lower than in medium containing 10 mg l^{-1} uracil (Table 4 and Table 8). This represents a shift to mixed-acid products 3-fold higher than that when uracil was limiting (Fig. 9A and 9C). The levels of acetate accumulated by strain D39 in uracil-enriched medium were at least 2-fold higher than those accumulated in normal CDM. However, the ATP yields (mol ATP mol^{-1} glucose) were similar between both conditions (*circa* 1.8 and 1.7 in 30 and 10 mg l^{-1} uracil, respectively) and relative to strain R6 (Table 4 and Table 8). Our data establish a positive correlation between uracil supply and production of capsule in *S. pneumoniae* serotype 2. Under the conditions studied, we showed that uracil is the factor limiting growth of strain D39, while the

acapsular strain R6 is irresponsive to the uracil concentration in the medium. The bioenergetic parameters fully support this view. In summary, we developed a CDM and growth conditions that support high yield of strains D39 and R6. For strain D39, a better growth performance was observed in CDM (Table 1) containing 1% (wt/vol) glucose and supplemented with uracil to a final concentration of 30 mg l^{-1}, under controlled conditions of temperature (37ºC), pH (6.5) and gas atmosphere (semi-aerobic, initial air tension of 50-60%). For strain R6, the best growth was observed in CDM containing 1% (wt/vol) glucose, under controlled conditions of temperature (37ºC), pH (6.5) and gas atmosphere (strictly anaerobic). The optimized conditions were used to perform *in vivo* NMR studies.

Glucose metabolism monitored by *in vivo* NMR

The use of the *in vivo* NMR technique to study bacterial metabolism has been reported before [21]. However, due to the low sensitivity of this technique, dense cell suspensions are usually required. Thus, we sought to prepare dense cell suspensions of strains D39 and R6. However, and despite several attempts and condition variations we were unable to pellet strain D39 by centrifugation. This is most likely a consequence of electrostatic repulsion due to its heavily negative charged capsule. In contrast, compact pellets were obtained upon centrifugation of R6 cultures, which enabled the preparation of dense cell suspensions and the application of *in vivo* NMR techniques to study sugar metabolism. For *in vivo* NMR studies, strain R6 was grown in CDM containing 1% (wt/vol) glucose, at 37ºC, pH 6.5 and under strictly anaerobic conditions (argon atmosphere). Cells were harvested in the late-exponential phase of growth (OD_{600} = 1.9, as in Fig. 3B), washed twice in 5 mM KP_i or MES buffer with 2% (wt/vol) choline and then suspended in 50 mM KP_i or MES buffer with 2% (wt/vol) choline to a protein concentration of 13-14 mg ml^{-1}. Choline was added to prevent cell lysis [55-57]. In buffer containing choline, lysis was marginal during the time span of ^{13}C-NMR experiments (30 min). The OD_{600} value of the suspension decreased by less than 3% in 30 min and 6.5% in 1h, while in the absence of choline the optical density values had

decreased by 40% and 85% after 30 and 60 min, respectively (Fig. S3). Glucose metabolism of R6 resting cells was monitored under controlled conditions of pH (6.5), temperature (37ºC) and gas atmosphere (anaerobic) using the on-line NMR system developed by Neves *et al.* (1999) [21].

(i) Pools of metabolites by *in vivo* ^{13}C-NMR. The time course for glucose consumption and product formation under anaerobic conditions is shown in Fig. 10. The end-products of [1-^{13}C]glucose (20 mM) metabolism were lactate (35.8 ± 0.4) mM), acetate (2.6 ± 0.4 mM) and glycerol (0.36 ± 0.04 mM). As expected lactate was the major end-product accounting for 89% of the glucose consumed. Interestingly, a 2 min delay for glucose consumption was observed after the pulse of glucose, which was then consumed at a maximal rate of 0.32 µmol min^{-1} mg^{-1} of protein (Fig. 10). The pool of fructose 1,6-bisphosphate (FBP) increased to a steady concentration of about 30 mM, and declined to undetectable levels at the onset of glucose exhaustion (Fig. 10). FBP was the only glycolytic metabolite detected. The glycolytic dynamics in *S. pneumoniae* are considerably different from those reported for the closely related organism *L. lactis* (reviewed in [20]). In *L. lactis*, at the onset of glucose depletion the pool of FBP declines to an intermediate level, and thereafter decreases slowly to undetectable concentrations (reviewed in [20]). Moreover, in *L. lactis*, 3-phosphoglycerate (3-PGA) and phospho*enol*pyruvate (PEP) accumulate after glucose depletion (reviewed in [20]). In the dairy bacterium, slow depletion of FBP and accumulation of 3-PGA and PEP were rationalized as resulting from progressive obstruction at the level of pyruvate kinase (PK), a glycolytic enzyme regulated at the metabolic level by FBP (activator) and inorganic phosphate (inhibitor) (reviewed in [20]). Based on the established differences between the metabolic profiles in *L. lactis* and *S. pneumoniae*, a different regulatory mechanism at the level of pyruvate kinase can be envisaged for the pathogen.

(ii) Pools of NTP and P_i by *in vivo* ^{31}P-NMR. To obtain information on the energetic status of the cells, *in vivo* ^{31}P-NMR spectra were obtained during the metabolism of glucose by resting cells of strain R6. Glucose (20 mM) was supplied at a time designated zero (0 min)

and the time course for formation of NTP and total P_i is shown in Fig. 11. Upon glucose addition, the pool of inorganic phosphate (P_i) decreased from about 60 to 2 mM, in 7 min, consistent with glycolytic usage. In accordance, this was also the time required for total consumption of glucose in the *in vivo* ^{13}C-NMR experiment of strain R6. During glucose consumption the NTP pools reached values of about 2.2 mM, which were maintained until the time-point 10 min, and disappeared afterwards. The pool of P_i became visible well after NTP depletion (after glucose disappearance) reaching values of approximately 60 mM. The build-up of the P_i pool in *S. pneumoniae* differs from that in *L. lactis*, for which P_i accumulates much earlier. Moreover, the NTP pools in *L. lactis* are 3-fold higher than in *S. pneumoniae* R6, and remain high beyond glucose depletion [20,21]. The levels of NTP detected *in vivo* in *S. pneumoniae* are similar to the concentrations measured *in vitro* [13]. In *S. pneumoniae*, the dynamics of glycolytic intermediates at the onset of glucose exhaustion (Fig. 10) could in part be explained by the delay in P_i accumulation, and thus lack of inhibition of PK. However, our own preliminary results indicate that the pneumococcal PK is less sensitive to P_i than its lactococcal homologue (data not shown). The effects of FBP and P_i on PK activity are currently under investigation in our laboratory.

Conclusions

A chemically defined medium and growth conditions supporting high biomass yields of *S. pneumoniae* strains D39 and R6 have been developed. Importantly, during this process an extensive comparative metabolic characterization between these strains under controlled conditions of pH, temperature and gas atmosphere has been accomplished. The results of these studies showed that strains D39 and R6 display a better growth performance under different environmental and nutritional conditions. In strain D39 the growth rate was stimulated under semi-aerobic conditions, while in strain R6 strictly anaerobic conditions rendered optimal growth. The better performance of D39 in semi-aerobic conditions was attributed to the lower activity of pyruvate oxidase and higher activity of NADH oxidase as

compared to R6. Furthermore, the maximal biomass achieved by strain D39 was substantially enhanced when a supplement of uracil was added to the culture medium, whereas R6 was irresponsive to this nucleobase. Our metabolic data showed that, more than an energetic burden capsule is a cost in nutritional terms and uracil is the limiting nutrient. This view indicates a direct link between capsule production and the requirement for uracil. Pyrimidine nucleobases and nucleosides are often unavailable as exogenous nutrients (re viewed in [58]). However, in blood, where capsule of *S. pneumoniae* is an absolute requirement, uridine was detected at a homeostatic concentration of about 3-4 µmol l^{-1} [59]. Replacement of uracil by uridine in CDM (Table 1) did not change the growth profile of strain D39 in this culture medium (data not shown). We hypothesize that uracil may be an important signal for capsule synthesis.

The surprising marked physiological differences between strains D39 and R6, shown in our studies substantiate the view that findings in strain R6 cannot be generalized to D39, at least when growth physiology is concerned [5]. Finally, the chemically defined medium optimized in this study was suitable for the application of *in vivo* NMR to study sugar metabolism. We are the first to obtain time series data on concentrations of metabolite pools online and non-invasively for *S. pneumoniae*. Our data suggest a unique regulation of glycolysis in *S. pneumoniae* as compared to other *Streptococcaceae*.

Materials and Methods

Bacterial strains, stocks preparation and storage

The *S. pneumoniae* strains used in this study were the serotype 2 D39 and its unencapsulated derivative R6 obtained from the Department of Molecular Biology of the University of Groningen. This strain D39 displays characteristics of the D39 Lilly isolate [5,29]. ***Long-term storage of strains D39 and R6.*** Permanent stock cultures (1 ml) were prepared in cryogenic vials from cells grown in M17 broth (Difco) supplemented with 0.5% (wt/vol) glucose (Glc-M17), harvested in exponential phase, and were maintained at -80ºC in

25% (vol/vol) glycerol. ***Preparation of stocks.*** D39 and R6 cells scraped from the permanent frozen stocks were grown overnight (~14 h) in 5 ml Glc-M17, at 37ºC and stored as 1 ml aliquots in 10% (vol/vol) glycerol at -80ºC. ***Working stocks preparation.*** Strains D39 and R6 were cultured by transferring 1 ml aliquot of the frozen stock cultures into 50 ml of Glc-M17, followed by incubation at 37ºC until late-exponential phase (OD_{600} 0.8 - 1.0). Cultures were then centrifuged (5750 × *g*, 7 min, 4ºC), the supernatants discarded and the pellets were concentrated 2-fold in fresh M17. Aliquots of 1 ml were stored in 10% (vol/vol) glycerol at -80ºC.

S. pneumoniae growth studies

S. pneumoniae was grown in the CDM described in Table 1 prepared in bi-distilled water (Millipore E-POD), except when stated otherwise. Growth was monitored hourly by measuring the optical density at 600 nm (OD_{600}). Batch cultivations were initiated at an OD_{600} of 0.05-0.06 by the addition of a preculture (3-4% vol/vol). The precultures were prepared as follows: 1 ml working stock (D39 or R6) was used to inoculate 80 ml of CDM buffered with disodium β-glycerophosphate and containing 60 mM glucose, in 100-ml static rubber-stoppered bottles; cultures were incubated 6-7 h (OD_{600} 0.8 - 1.0) at 37ºC, without pH control (initial pH 6.5). Incubation was kept below 7 h to avoid using stationary phase cells for inoculation. Preculturing allows for adjustment of the bacteria to the culture medium and decreases culture variability. Specific growth rates (µ) were calculated through linear regressions of the plots of ln(OD_{600}) *versus* time during the exponential growth phase. Figures throughout this chapter show individual growth curves from representative experiments.

Batch cultivations without pH control. For *S. pneumoniae* cultures grown without pH control (initial pH 6.5) the CDM was buffered with disodium β-glycerophosphate (higher buffering capacity than phosphate buffer) and supplemented with 60 mM glucose. Routinely, cells were cultivated in static rubber-stoppered bottles (semi-aerobic conditions, 80 ml in

100-ml bottles); for aerobic conditions cells were grown in shake flasks (CDM volume 1/5 of the flasks total capacity) in an orbital shaker (AGITORB 200, Aralab) at 150 rpm. For each growth condition at least two independent experiments were performed. The error in each point of the growth curves was always below 15%.

The uracil-dependency on growth of strain D39 was assessed by cultivating cells in semi-aerobic conditions as above, except that uracil was omitted or added to the medium in the following concentrations: 0.67, 1, 3.3, 5, 10, 30 and 40 mg l^{-1}. Two independent experiments were performed at least for each growth condition. The error in each point of the growth curves was always below 10%.

The effect of nucleobases (xanthine, adenine, guanine and uracil) on the growth of strain D39 was tested as follows: cultures of 250 µl were prepared in CDM containing 0.25% (wt/vol) glucose and each nitrogenous base was added individually to a final concentration of 30 mg l^{-1}. Cultures were started at an initial OD_{595} of 0.25-0.3, by addition of an exponential growing preculture suspended in fresh CDM without nucleobases, and grown for 24 h at 37°C in 96-well microtiter plates. Growth was monitored every 30 min at 595 nm with a ELx808 microplate spectrophotometer (BioTek Instruments, Inc.), and growth curves generated by using Gen5™ (BioTek Instruments, Inc.). Each growth condition was done in triplicate using two independent precultures. The error in each point of the growth curves was always below 5%.

Batch cultivations in bioreactors with pH control. D39 and R6 strains were grown in CDM in a 2-l bioreactor (Sartorius Biostat® B plus) with the pH-controlled at 6.5, and under anaerobic (argon atmosphere), semi-aerobic (initial specific air tension of 50-60%) or aerobic (continuous specific air tension of 40%) conditions. For anaerobic growth, the medium was degassed by flushing argon overnight preceding inoculation, and the headspace was continuously sparged with argon at a rate of 50 ml min^{-1} during growth. For the semi-aerobic conditions and aerobic growth, dissolved oxygen was monitored with a polarographic oxygen electrode (Mettler-Toledo International). The electrode was calibrated to zero or 100% by bubbling sterile argon or air through the medium, respectively. The continuous specific air

tension of 40% in the culture medium was maintained by automatic control of the airflow and agitation. Specific air tension consumption over time in growth under semi-aerobic conditions was registered in the MFCS/DA software (B. Braun Biotech International) coupled to the fermentation unit. Independently of the growth condition, glucose was used as carbon source at a final concentration of about 60 mM, except when the effect of glucose concentration on growth was tested. For these experiments, glucose was added to the medium at concentrations of 0.5%, 1% and 3% (wt/vol). To investigate the effect of nucleobases (Table 1) on growth, their concentrations were raised from 10 to 30 mg l^{-1} in the culture medium. To test the effect of uracil *per se* on growth of strains D39 and R6 the concentration of this nucleobase was increased from 10 to 30 mg l^{-1}. The pH was kept at 6.5 by the automatic addition of 10 M NaOH, and the temperature was set to 37ºC. Under anaerobic and semi-aerobic conditions culture homogenization was achieved by maintaining an agitation speed of 70 rpm. For each growth condition at least two independent experiments were performed. The error in each point of the plotted growth curves was always below 20%.

Kinetics of oxygen consumption

Oxygen concentration in the culture medium was calculated using the equation of Henry's Law: X_{O2} (mol O_2 mol^{-1} H_2O) = P_{O2}/H. The partial pressure of oxygen (P_{O2}, atm) in the culture medium was calculated by multiplying the percentage of air in the culture medium by the percentage of O_2 in an atmosphere saturated with air (20.95% (vol/vol) of O_2). The Henry's Law constant (H) for O_2 at 37ºC is 5.18 x 10^{-4} atm mol^{-1} O_2 mol^{-1} H_2O. Water concentration (mol H_2O l^{-1} H_2O) is 55.5 M. The oxygen consumption rate (q_s^{max} in Fig. S1) was estimated from a first-order derivative of a polynomial fit of the observed O_2 consumption time series. Dry weight (DW) was used as a measure of cell mass.

Quantification of glucose and fermentation products

Strains were grown in CDM supplemented with glucose and with pH control. Culture samples (2 ml) were taken at different time-points of growth, centrifuged (16,000 × *g*, 2 min, 4ºC), filtered (Millex-GN 0.22 µm filters) and the supernatant solutions were stored at -20ºC until analysis by high performance liquid chromatography (HPLC). Substrates and end-products were quantified as before [13], in an HPLC apparatus equipped with a refractive index detector (Shodex RI-101, Showa Denko K. K.) using an HPX-87H anion exchange column (Bio-Rad Laboratories Inc.) at 60ºC, with 5 mM H_2SO_4 as the elution fluid and a flow rate of 0.5 ml min^{-1}. Alternatively, quantification of metabolites in the supernatant solutions was performed by ^{1}H-NMR in a Bruker AMX300 spectrometer (Bruker BioSpin GmbH). Formic acid (sodium salt) was added to the samples and used as an internal concentration standard. The ATP yield was calculated as the ratio of ATP produced to glucose consumed. The global yields of ATP were calculated from the fermentation products determined at the time-point of growth arrest assuming that all ATP was synthesized by substrate-level phosphorylation. A factor of 0.39, determined from a DW (mg ml^{-1}) *versus* OD_{600} curve, was used to convert OD_{600} into dry weight (mg biomass ml^{-1}). For the aerobic samples, hydrogen peroxide was quantified in fresh supernatant solutions as described below.

Determination of hydrogen peroxide (H_2O_2)

Hydrogen peroxide was determined in supernatants of cultures grown under semi-aerobic and aerobic conditions and with pH set to 6.5. Culture samples of 1-ml were harvested at different time-points of the growth curves, centrifuged (16,000 × *g*, 2 min, 4ºC) and filtered (Millex-GN 0.22 µm filters). The Amplex® Red Hydrogen Peroxide/Peroxidase assay kit (Invitrogen) was used to quantify H_2O_2 contents below 10 µM. Determinations of H_2O_2 up to 300 µM were performed as described elsewhere [60]. Briefly, 1.25 ml of peroxide reagent (192 mM phosphate, 14.8 mM azide, 0.96 ml l^{-1} Triton X-100, 2 KU l^{-1} horseradish peroxidase (Roche), 0.48 mM 4-aminophenazone and 9.6 mM chromotropic acid) was added to 50 µl of supernatant, mixed and allowed to stand for 5 minutes at room temperature. In the

presence of H_2O_2, the chromotropic acid was converted by the peroxidase into a blue coloured compound with maximal absorbance at 600 nm. Absorbance was read at 600 nm. Water was used as the blank and standard curves were performed with fresh dilutions of a stabilized solution of 30% (wt/vol) H_2O_2. The absorbance of the samples was compared to that of the standard solutions.

Enzymatic activities

(i) Pyruvate oxidase activity. Cell lysates and pyruvate oxidase activity were performed as described in [41] with minor modifications. Cells grown aerobically with pH control were harvested in late-exponential phase of growth (R6, OD_{600} 0.16 ± 0.00; D39, OD_{600} 0.29 ± 0.06), centrifuged (5750 × *g*, 5 min, 4ºC), and the pellets washed twice in one volume of 50 mM KP_i, pH 7.4. Cells were re-suspended in 0.1 volume of the same buffer containing 0.1% Triton X-100, and incubated for 10 min at 37ºC. Reactions for determination of pyruvate oxidase activity contained 50 mM potassium phosphate (pH 6.0), 5 mM $MgSO_4$, 0.5 mM thiamine pyrophosphate, 0.1 mM FAD, 15 mM sodium pyruvate, 0.2 U ml^{-1} horseradish peroxidase, 100 µM Amplex® Red Reagent (Invitrogen) and 10 µl of cell lysate. Standard curves were performed with fresh dilutions of a stabilized solution of 30% (wt/vol) of H_2O_2. The assays were incubated at 37ºC and the absorbance of the reaction was read in a SmartSpec^{TM}Plus spectrophotometer (BioRad) at 563 nm every 5 min for 1h.

(ii) NADH oxidase activity. NADH oxidase activity was determined in cells grown without pH control under semi-aerobic and aerobic conditions. Cells were harvested in late-exponential phase of growth (Fig. 1), and cell lysates prepared as above. NADH oxidase activity was assayed spectrophotometrically (Beckman DU70) at 37ºC in a total volume of 1 ml containing 100 mM Tris-HCl buffer, pH 7.2, 5 mM $MgCl_2$, and 0.29 mM NADH. The reaction was initiated by the addition of an adequate amount of cell lysate and monitored by the decrease in absorbance at 340 nm. One unit of enzyme activity was defined as the

amount of enzyme catalyzing the conversion of 1 µmol of substrate per minute under the experimental conditions used.

(iii) Lactate dehydrogenase activity. Lactate dehydrogenase (LDH) activity was determined in cells grown without pH control under semi-aerobic conditions. Cells were grown until late-exponential phase, centrifuged (5750 × *g*, 5 min, 4ºC), washed with KP_i 10 mM (pH 7.0) and suspended in the same buffer. The cell suspensions were disrupted in a French Press (SLM Aminco Instruments, Golden Valley, MN, USA) at 36 MPa. LDH activity was assayed spectrophotometrically by NADH measurement, as described elsewhere [61], except that the temperature was kept at 37ºC.

In vivo ^{13}C-NMR experiments

S. pneumoniae R6 cells (2l) were grown under anaerobic conditions with pH control as described above, harvested in the late-exponential phase of growth (OD_{600} 1.9), centrifuged (5750 × *g*, 7 min, 4ºC), washed twice with 5 mM KP_i buffer with 2% (wt/vol) choline, pH 6.5 (5750 × *g*, 5 min, 4ºC) and suspended to a protein concentration of 13-14 mg ml^{-1} in 50 mM KP_i with 2% (wt/vol) choline, pH 6.5. Deuterium oxide (^2H_2O) was added to a final concentration of 6% (vol/vol) to provide a lock signal. NMR experiments were performed using the on-line system described elsewhere, which consists of a mini-bioreactor (50 ml working volume) coupled to NMR detection with a circulating system that allows for non-invasive studies of metabolism under controlled conditions of pH, gas atmosphere and temperature [21]. Glucose specifically labeled with ^{13}C on carbon one (20 mM) was added to the cell suspension at time-point zero and spectra (30 s) acquired sequentially after its addition. The time course of glucose consumption, product formation, and changes in the pools of intracellular metabolites were monitored *in vivo*. At the end of the *in vivo* NMR experiment the cell suspension was passed through a French press: the resulting cell extract was incubated at 80-90°C (10 min) in a stoppered tube, cooled down on ice and cell debris and denatured macromolecules were removed by centrifugation. The supernatant (NMR-

extract) was used for quantification of end-products and minor metabolites as below. Due to the fast pulsing conditions used for acquiring *in vivo* ^{13}C-spectra, correction factors for resonances due to C1 and C6 of FBP (0.73 ± 0.02) were determined to convert peak intensities into concentrations as described by Neves *et al.* (2002) [45], except that the temperature was kept at 37ºC. The quantitative kinetic data for intracellular metabolites were calculated as described elsewhere [21]. The lower limit for *in vivo* NMR detection of intracellular metabolites under these conditions was 3-4 mM. Intracellular metabolite concentrations were calculated using a value of 3.0 µl (mg of protein)$^{-1}$ determined for the intracellular volume of *S. pneumoniae* as in Ramos-Montañez *et al.* (2010) [31]. Although individual experiments are illustrated in each figure, each type of *in vivo* NMR experiment was repeated at least twice and the results were highly reproducible. The values reported are averages of two experiments and the accuracy varied from 5% to 15% in the case of metabolites with concentrations below 5 mM.

In vivo ^{31}P-NMR experiments

Cell suspensions were prepared as above, except that 50 mM MES buffer, pH 6.5, was used. Glucose (20 mM) was added to the cell suspension at time-point zero and spectra (2 min 6 s) acquired sequentially after its addition. Pools of NTP and inorganic phosphate (P_i) were obtained in real time non-invasively.

Quantification of products by NMR

Lactate and acetate were quantified in NMR-extracts by ^{1}H-NMR [21]. Formic acid (sodium salt) was added to the samples and used as an internal concentration standard. The concentration of minor products (glycerol, glycerate, alanine, aspartate, ethanol) and metabolic intermediates that remained inside the cells (3-phosphoglycerate, 3-PGA) was determined from the analysis of ^{13}C spectra of NMR-extracts as described by Neves *et al.*

(1999) [21]. The concentration of labeled lactate determined by ^{1}H-NMR was used as a standard to calculate the concentration of the other metabolites in the sample.

NMR Spectroscopy

Carbon-13 and phosphorus-31 spectra were acquired at 125.77 MHz and 202.48 MHz, respectively on a Bruker AVANCE II 500 MHz spectrometer (Bruker BioSpin GmbH). All *in vivo* experiments were run using a quadruple nuclei probe head at 37ºC as described elsewhere [21]. Acquisition of ^{31}P-NMR and ^{13}C-NMR spectra was performed as described by Neves *et al.* (1999) [21]. For calculation of the correction factors ^{13}C-NMR spectra were acquired with a 60º flip angle and a recycle delay of 1.5 s (saturating conditions) or 60.5 s (relaxed conditions). Carbon and phosphorus chemical shifts are referenced to the resonance of external methanol and H_3PO_4 (85% vol/vol) designated at 49.3 and 0.0 ppm, respectively.

References

1. Mitchell TJ (2003) The pathogenesis of streptococcal infections: from tooth decay to meningitis. Nat Rev Microbiol 1: 219-230.

2. Kadioglu A, Weiser JN, Paton JC, Andrew PW (2008) The role of *Streptococcus pneumoniae* virulence factors in host respiratory colonization and disease. Nat Rev Microbiol 6: 288-301.

3. Tettelin H, Nelson KE, Paulsen IT, Eisen JA, Read TD, et al. (2001) Complete genome sequence of a virulent isolate of *Streptococcus pneumoniae*. Science 293: 498-506.

4. Hoskins J, Alborn WE, Arnold J, Blaszczac LC, Burgett S, et al. (2001) Genome of the bacterium *Streptococcus pneumoniae* strain R6. J Bacteriol 183: 5709-5717.

5. Lanie JA, Ng WL, Kazmierczak KM, Andrzejewski TM, Davidsen TM, et al. (2007) Genome sequence of Avery's virulent serotype 2 strain D39 of *Streptococcus pneumoniae* and comparison with that of unencapsulated laboratory strain R6. J Bacteriol 189: 38-51.

6. Shelburne SA, Davenport MT, Keith DB, Musser JM (2008) The role of complex carbohydrate catabolism in the pathogenesis of invasive streptococci. Trends Microbiol 16: 318-325.

7. Giammarinaro P, Paton JC (2002) Role of RegM, a homologue of the catabolite repressor protein CcpA, in the virulence of *Streptococcus pneumoniae*. Infect Immun 70: 5454-5461.

8. Iyer R, Baliga NS, Camilli A (2005) Catabolite control protein A (CcpA) contributes to virulence and regulation of sugar metabolism in *Streptococcus pneumoniae*. J Bacteriol 187: 8340-8349.

9. Kaufman GE, Yother J (2007) CcpA-dependent and -independent control of beta-galactosidase expression in *Streptococcus pneumoniae* occurs via regulation of an upstream phosphotransferase system-encoding operon. J Bacteriol 189: 5183-5192.

10. Iyer R, Camilli A (2007) Sucrose metabolism contributes to *in vivo* fitness of *Streptococcus pneumoniae*. Mol Microbiol 66: 1-13.

11. Trappetti C, Kadioglu A, Carter M, Hayre J, Iannelli F, et al. (2009) Sialic acid: a preventable signal for pneumococcal biofilm formation, colonization, and invasion of the host. J Infect Dis 199: 1497-1505.

12. Yesilkaya H, Spissu F, Carvalho SM, Terra VS, Homer KA, et al. (2009) Pyruvate formate lyase is required for pneumococcal fermentative metabolism and virulence. Infect Immun 77: 5418-5427.

13. Carvalho SM, Kloosterman TG, Kuipers OP, Neves AR (2011) CcpA ensures optimal metabolic fitness of *Streptococcus pneumoniae*. PLoS One 6: e26707.

14. Marion C, Burnaugh AM, Woodiga SA, King SJ (2011) Sialic acid transport contributes to pneumococcal colonization. Infect Immun 79: 1262-1269.

15. Marion C, Stewart JM, Tazi MF, Burnaugh AM, Linke CM, et al. (2012) *Streptococcus pneumoniae* can utilize multiple sources of hyaluronic acid for growth. Infect Immun 80: 1390-1398.

16. Heinemann M, Sauer U (2010) Systems biology of microbial metabolism. Curr Opin Microbiol 13: 337-343.

17. Griffin TJ, Gygi SP, Ideker T, Rist B, Eng J, et al. (2002) Complementary profiling of gene expression at the transcriptome and proteome levels in *Saccharomyces cerevisiae*. Mol Cell Proteomics 1: 323-333.

18. Daran-Lapujade P, Rossell S, van Gulik WM, Luttik MA, de Groot MJ, et al. (2007) The fluxes through glycolytic enzymes in *Saccharomyces cerevisiae* are predominantly regulated at posttranscriptional levels. Proc Natl Acad Sci U S A 104: 15753-15758.

19. Hathaway LJ, Brugger SD, Morand B, Bangert M, Rotzetter JU, et al. (2012) Capsule type of *Streptococcus pneumoniae* determines growth phenotype. PLoS Pathog 8: e1002574.

20. Neves AR, Pool WA, Kok J, Kuipers OP, Santos H (2005) Overview on sugar metabolism and its control in *Lactococcus lactis* - the input from *in vivo* NMR. FEMS Microbiol Rev 29: 531-554.

21. Neves AR, Ramos A, Nunes MC, Kleerebezem M, Hugenholtz J, et al. (1999) *In vivo* nuclear magnetic resonance studies of glycolytic kinetics in *Lactococcus lactis*. Biotechnol Bioeng 64: 200-212.

22. Cocaign-Bousquet M, Garrigues C, Novak L, Lindley ND, Loubière P (1995) Rational development of a simple synthetic medium for the sustained growth of *Lactococcus lactis*. J Appl Bacteriol 79: 108-116.

23. Zhang G, Mills DA, Block DE (2009) Development of chemically defined media supporting high-cell-density growth of lactococci, enterococci, and streptococci. Appl Environ Microbiol 75: 1080-1087.

24. Tomasz A (1964) A chemically defined medium for *Streptococcus pneumoniae*. Bacteriol Proc 64: 29.

25. Sicard AM (1964) A new synthetic medium for *Diplococcus pneumoniae*, and its use for the study of reciprocal transformations at the *amiA locus*. Genetics 50: 31-44.

26. Kloosterman TG, Bijlsma JJ, Kok J, Kuipers OP (2006) To have neighbour's fare: extending the molecular toolbox for *Streptococcus pneumoniae*. Microbiology 152: 351-359.

27. Gonçalves VM, Takagi M, Lima RB, Massaldi H, Giordano RC, et al. (2003) Purification of capsular polysaccharide from *Streptococcus pneumoniae* serotype 23F by a procedure suitable for scale-up. Biotechnol Appl Biochem 37: 283-287.

28. Massaldi H, Bessio MI, Suarez N, Texeira E, Rossi S, et al. (2010) Features of bacterial growth and polysaccharide production of *Streptococcus pneumoniae* serotype 14. Biotechnol Appl Biochem 55: 37-43.

29. Avery OT, Macleod CM, McCarty M (1944) Studies on the chemical nature of the substance inducing transformation of pneumococcal types: induction of transformation by a desoxyribonucleic acid fraction isolated from pneumococcus type III. J Exp Med 79: 137-158.

30. Tomasz A (1967) Choline in the cell wall of a bacterium: novel type of polymer-linked choline in Pneumococcus. Science 157: 694-697.

31. Ramos-Montañez S, Kazmierczak KM, Hentchel KL, Winkler ME (2010) Instability of *ackA* (acetate kinase) mutations and their effects on acetyl phosphate and ATP amounts in *Streptococcus pneumoniae* D39. J Bacteriol 192: 6390-6400.

32. Alloing G, de Philip P, Claverys JP (1994) Three highly homologous membrane-bound lipoproteins participate in oligopeptide transport by the Ami system of the gram-positive *Streptococcus pneumoniae*. J Mol Biol 241: 44-58.

33. Béal C, Louvet P, Corrieu G (1989) Influence of controlled pH and temperature on the growth and acidification of pure cultures of *Streptococcus thermophilus* 404 and *Lactobacillus bulgaricus* 398. Appl Microbiol Biotechnol 32: 148-154.

34. Mercade M, Lindley ND, Loubière P (2000) Metabolism of *Lactococcus lactis* subsp. *cremoris* MG 1363 in acid stress conditions. Int J Food Microbiol 55: 161-165.

35. Tomasz A, Albino A, Zanati E (1970) Multiple antibiotic resistance in a bacterium with suppressed autolytic system. Nature 227: 138-140.

36. Lopez R, Garcia E (2004) Recent trends on the molecular biology of pneumococcal capsules, lytic enzymes, and bacteriophage. FEMS Microbiol Rev 28: 553-580.

37. Regev-Yochay G, Trzcinski K, Thompson CM, Lipsitch M, Malley R (2007) SpxB is a suicide gene of *Streptococcus pneumoniae* and confers a selective advantage in an *in vivo* competitive colonization model. J Bacteriol 189: 6532-6539.

38. Kolter R, Siegele DA, Tormo A (1993) The stationary phase of the bacterial life cycle. Annu Rev Microbiol 47: 855-874.

39. Navarro Llorens JM, Tormo A, Martínez-García E (2010) Stationary phase in Gram-negative bacteria. FEMS Microbiol Rev 34: 476-495.

40. Taniai H, Iida K, Seki M, Saito M, Shiota S, et al. (2008) Concerted action of lactate oxidase and pyruvate oxidase in aerobic growth of *Streptococcus pneumoniae*: role of lactate as an energy source. J Bacteriol 190: 3572-3579.

41. Belanger AE, Clague MJ, Glass JI, LeBlanc DJ (2004) Pyruvate oxidase is a determinant of Avery's rough morphology. J Bacteriol 186: 8164-8171.

42. Ramos-Montañez S, Tsui HC, Wayne KJ, Morris JL, Peters LE, et al. (2008) Polymorphism and regulation of the *spxB* (pyruvate oxidase) virulence factor gene by a CBS-HotDog domain protein (SpxR) in serotype 2 *Streptococcus pneumoniae*. Mol Microbiol 67: 729-746.

43. Echenique JR, Chapuy-Regaud S, Trombe MC (2000) Competence regulation by oxygen in *Streptococcus pneumoniae*: involvement of *ciaRH* and *comCDE*. Mol Microbiol 36: 688-696.

44. Higuchi M, Shimada M, Yamamoto Y, Hayashi T, Koga T, et al. (1993) Identification of two distinct NADH oxidases corresponding to H_2O_2-forming oxidase and H_2O-forming oxidase induced in *Streptococcus mutans*. J Gen Microbiol 139: 2343-2351.

45. Neves AR, Ventura R, Mansour N, Shearman C, Gasson MJ, et al. (2002) Is the glycolytic flux in *Lactococcus lactis* primarily controlled by the redox charge? Kinetics of NAD^+ and NADH pools determined *in vivo* by ^{13}C-NMR. J Biol Chem 277: 28088-28098.

46. Pericone CD, Overweg K, Hermans PW, Weiser JN (2000) Inhibitory and bactericidal effects of hydrogen peroxide production by *Streptococcus pneumoniae* on other inhabitants of the upper respiratory tract. Infect Immun 68: 3990-3997.

47. Pericone CD, Park S, Imlay JA, Weiser JN (2003) Factors contributing to hydrogen peroxide resistance in *Streptococcus pneumoniae* include pyruvate oxidase (SpxB) and avoidance of the toxic effects of the Fenton reaction. J Bacteriol 185: 6815-6825.

48. Auzat I, Chapuy-Regaud S, Le Bras G, dos Santos D, Ogunniyi AD, et al. (1999) The NADH oxidase of *Streptococcus pneumoniae*: its involvement in competence and virulence. Mol Microbiol 34: 1018-1028.

49. Yu J, Bryant AP, Marra A, Lonetto MA, Ingraham KA, et al. (2001) Characterization of the *Streptococcus pneumoniae* NADH oxidase that is required for infection. Microbiology 147: 431-438.

50. Seki M, Iida K, Saito M, Nakayama H, Yoshida S (2004) Hydrogen peroxide production in *Streptococcus pyogenes*: involvement of lactate oxidase and coupling with aerobic utilization of lactate. J Bacteriol 186: 2046-2051.

51. Quatravaux S, Remize F, Bryckaert E, Colavizza D, Guzzo J (2006) Examination of *Lactobacillus plantarum* lactate metabolism side effects in relation to the modulation of aeration parameters. J Appl Microbiol 101: 903-912.

52. Iannelli F, Pearce BJ, Pozzi G (1999) The type 2 capsule locus of *Streptococcus pneumoniae*. J Bacteriol 181: 2652-2654.

53. Papagianni M, Avramidis N, Filiousis G (2007) Glycolysis and the regulation of glucose transport in *Lactococcus lactis* spp. *lactis* in batch and fed-batch culture. Microb Cell Fact 6: 16.

54. Kilstrup M, Hammer K, Ruhdal JP, Martinussen J (2005) Nucleotide metabolism and its control in lactic acid bacteria. FEMS Microbiol Rev 29: 555-590.

55. Giudicelli S, Tomasz A (1984) Attachment of pneumococcal autolysin to wall teichoic acids, an essential step in enzymatic wall degradation. J Bacteriol 158: 1188-1190.

56. Balachandran P, Hollingshead SK, Paton JC, Briles DE (2001) The autolytic enzyme LytA of *Streptococcus pneumoniae* is not responsible for releasing pneumolysin. J Bacteriol 183: 3108-3116.

57. Steinmoen H, Knutsen E, Havarstein LS (2002) Induction of natural competence in *Streptococcus pneumoniae* triggers lysis and DNA release from a subfraction of the cell population. Proc Natl Acad Sci U S A 99: 7681-7686.

58. Turnbough CL, Switzer RL (2008) Regulation of pyrimidine biosynthetic gene expression in bacteria: repression without repressors. Microbiol Mol Biol Rev 72: 266-300.

59. Simmonds RJ, Harkness RA (1981) High-performance liquid chromatographic methods for base and nucleoside analysis in extracellular fluids and in cells. J Chromatog 226: 369-381.

60. Meiattini F (1988) Inorganic peroxides. In: Bergmeyer HU, Bergmeyer J, Grabl M, editors. Methods of enzymatic analysis. Verlag-Chemie, Weinheim, Germany: Vol. VII, pp. 566-571.

61. Gaspar P, Neves AR, Shearman CA, Gasson MJ, Baptista AM, et al. (2007) The lactate dehydrogenases encoded by the *ldh* and *ldhB* genes in *Lactococcus lactis* exhibit distinct regulation and catalytic properties - comparative modeling to probe the molecular basis. FEBS J 274: 5924-5936.

Figure Legends

Figure 1. Growth profiles of strains D39 and R6 without pH control. Growth of strains D39 (□) and R6 (■) in CDM containing 60 mM glucose, without pH control (initial pH of 6.5), at 37ºC, under different oxygen atmospheres. (A) precultures (graph above) and cultures (graph below) were grown under semi-aerobic conditions in static rubber-stoppered bottles. (B) cultures were grown aerobically in shake flasks at 150 rpm. OD_{600} was measured at 1-h intervals. Cultures were inoculated with a preculture in late-exponential phase (6-7 hours incubation at 37ºC, OD_{600} 0.8-1.0). The arrows indicate the time-points at which cells were harvested for measurement of NADH oxidase and LDH activities. The growth rates for each culture grown semi-aerobically and aerobically are also indicated and the values are averages ± SD.

Figure 2. Effect of preculture age on growth of strain R6 under controlled environmental conditions. Growth of strain R6 in CDM containing 60 mM glucose, under controlled conditions of pH (6.5), temperature (37ºC) and atmosphere (anaerobiosis with argon atmosphere), in a 2-l bioreactor. OD_{600} was measured at 1-h intervals. Symbols: (■), inoculation with a preculture in late-exponential phase (LExp, 6-7 hours of incubation at 37ºC); (◇), inoculation with a preculture in early-stationary phase (EStat, 8-9 hours of incubation at 37ºC); (△), inoculation with a preculture in late-stationary phase (LStat, 18 hours of incubation at 37ºC).

Figure 3. Effect of oxygen on growth profiles of strains D39 and R6 under controlled conditions. Growth of strains D39 (□) and R6 (■) in CDM containing 61 ± 1 mM glucose, under controlled conditions of pH (6.5), temperature (37ºC) and atmosphere (A) semi-aerobiosis (initial specific air tension of 50-60%) (B) anaerobiosis with argon atmosphere (C) aerobiosis (continuous specific air tension of 40%), in a 2-l bioreactor. OD_{600} was measured

at 1-h intervals or less for aerobic growths. All cultures were inoculated with a preculture in late-exponential phase (6-7 hours incubation at 37ºC, OD_{600} 0.8-1.0).

Figure 4. Schematic overview of the pathways for glucose metabolism and capsule production in *S. pneumoniae*. Glucose is oxidized to pyruvate via the Embden-Meyerhof-Parnas pathway (glycolysis); a common metabolic intermediate in glycolysis is glucose 6-phosphate (G6P), which is also a precursor for the biosynthesis of capsule NDP-sugars; NDP-sugars are synthesized at the expense of UTP. Pyruvate is the substrate of three competing enzymes: lactate dehydrogenase, pyruvate formate-lyase and pyruvate oxidase. Homolactic fermentation reduces pyruvate into lactate, through lactate dehydrogenase (LDH), whereas mixed-acid fermentation leads to formate, acetate and ethanol. Oxygen might be consumed at the level of lactate oxidase (LOX coded by *lcto*), pyruvate oxidase (SpxB) or H_2O-NADH oxidase (NOX). The occurrence of the pyruvate dehydrogenase complex (PDHc) depicted in grey remains to be proved. Proposed pathways were reconstructed based on genome database (http://www.ncbi.nlm.nih.gov/genomes/lproks.cgi), literature and database surveys (KEGG, MetaCyc). Gene annotation downloaded from NCBI: *nox*, NADH oxidase; *pyk,* pyruvate kinase; *ldh*, L-lactate dehydrogenase; *lcto*, lactate oxidase; *spxB*, pyruvate oxidase; *ackA*, acetate kinase; *pfl*, pyruvate formate-lyase; *pta*, phosphotransacetylase; *adh*, bifunctional acetaldehyde-CoA/alcohol dehydrogenase; *PDHc*, putative pyruvate dehydrogenase complex.

Figure 5. Fermentation profiles of strains D39 and R6 under aerobic conditions. Growth curves, substrate consumption and end-products formed by the D39 (A) and R6 (B) strains growing aerobically (continuous specific air tension of 40%) as in Fig. 3C. Culture supernatant samples for end-product analysis by HPLC and/or ^{1}H-NMR were harvested during growth. Symbols: (○), Glucose consumption; (□), growth curve of D39; (■), growth

curve of R6; (◆), lactate; (▲), acetate; (●), H_2O_2. The error was below 7% for major products (>2 mM) and 25% for minor products (<2 mM).

Figure 6. Growth profiles of D39 and R6 at different glucose concentrations. Growth of strains D39 (A) and R6 (B) in CDM containing (◇,◆) 0.5%, (□,■) 1% or (○,●) 3% (wt/vol) glucose, under controlled conditions of pH (6.5), temperature (37ºC) and atmosphere (initial specific air tension of 50-60%), in a 2-l bioreactor. OD_{600} was measured at 1-h intervals. All cultures were inoculated with a preculture in late-exponential phase (6-7 hours incubation at 37ºC, OD_{600} 0.8-1.0).

Figure 7. Effect of nucleobases concentration on growth profiles. Growth of strains D39 (A) and R6 (B) in CDM containing 1% (wt/vol) glucose, under controlled conditions of pH (6.5), temperature (37ºC), and atmosphere (initial specific air tension of 50-60%), in a 2-l bioreactor. The nucleobases were added to the medium as follows: (□,■), G, A, X, U 10 mg l^{-1} each; (△,▲), G, A, X, U 30 mg l^{-1} each; (○,●), G, A, X 10 mg l^{-1} each plus 30 mg l^{-1} U. G= guanine; A= Adenine; X= Xanthine; U= Uracil. OD_{600} was measured at 1-h intervals. All cultures were inoculated with a preculture in late-exponential phase (6-7 hours incubation at 37ºC, OD_{600} 0.8-1.0).

Figure 8. Effect of varying uracil concentration on growth of strain D39. (A) Growth of strain D39 in CDM containing 1% (wt/vol) glucose with different concentrations of uracil (U), as specified below. Growth was performed at 37ºC, without pH control (initial pH 6.5), in static rubber-stoppered bottles, after inoculation with a preculture in late-exponential phase (6-7 hours incubation at 37ºC, OD_{600} 0.8-1.0). OD_{600} was measured at 1-h intervals. Uracil concentrations in mg l^{-1}: (□), 0; (△), 0.67; (◇), 1; (○), 3.3; (▲), 5; (■), 10; (●), 30; (◆), 40. The other three nucleobases (G, X, A) were always present in the medium at a concentration of 10 mg l^{-1}. G=guanine; X, xanthine; A, adenine. (B) Linear correlation between the maximal D39 biomass (OD_{Max}) and the medium uracil concentration (from 0.67 to 10 mg l^{-1}).

Figure 9. Growth and fermentation products as a function of uracil concentration in pH-controlled batch cultures. Growth curves, substrate consumption and end-products formed by the D39 (A and C) and R6 (B and D) strains in CDM containing 10 mg l^{-1} uracil (A and C) or 30 mg l^{-1} uracil (B and D) under controlled conditions of pH (6.5), temperature (37ºC) and gas atmosphere (initial specific air tension of 50-60%) in a 2-l bioreactor after inoculation with a preculture in late-exponential phase (6-7 hours incubation at 37ºC, OD_{600} 0.8-1.0). Initial concentrations of glucose were for both strains 60 ± 1 mM. At 1-h time intervals OD_{600} was measured. Culture supernatant samples for end-product analysis by HPLC and/or ^{1}H-NMR were harvested for each of the conditions at time-points zero, exponential and growth arrest (maximal biomass) (bars in the plots). Symbols: (□,■), growth curves in 10 mg l^{-1} uracil; (○,●), growth curves in 30 mg l^{-1} uracil; (white bars), glucose; (grey bars), lactate; (hatched bars), formate; (black bars), acetate; (stripped bars), ethanol. D39 and R6 growth curves in 10 and 30 mg l^{-1} uracil as in Fig. 7, except that OD_{600} scale (y-axis) is logarithmic.

Figure 10. Glucose metabolism in resting cell suspensions of *S. pneumoniae* R6 monitored by *in vivo* ^{13}C-NMR. Kinetics of 20 mM [1-^{13}C]glucose consumption, end-products formation and build-up of glycolytic intermediate pools by a cell suspension (50 ml) of resting cells of *S. pneumoniae* R6 under anaerobic conditions as monitored *in vivo* by ^{13}C-NMR. R6 cells, grown anaerobically as in Fig. 3B until late-exponential phase (OD_{600} 1.9), were suspended in 50 mM KP_i with 2% (wt/vol) choline (pH 6.5) at a concentration of 13-14 mg protein ml^{-1}. [1-^{13}C]glucose was added at time zero and 30 s spectra were acquired sequentially after its addition. The extracellular pH was maintained at 6.5 during the experiment by automatic addition of NaOH. Symbols: (◆), glucose; (□), lactic acid; (○), acetate; (◇), glycerol; (△), fructose 1,6-bisphosphate.

Figure 11. Determination of the pools of NTP and P_i by *in vivo* ^{31}P-NMR. Time course for the concentration of NTP (◆) and total inorganic phosphate, P_i (●) in resting cells of *S. pneumoniae* R6 under anaerobic conditions as monitored *in vivo* by ^{31}P-NMR. R6 cells, grown anaerobically as in Fig. 3B until late-exponential phase (OD_{600} 1.9), were suspended in 50 mM MES buffer with 2% (wt/vol) choline (pH 6.5) at a concentration of 13-14 mg protein ml^{-1}. Glucose was added at time zero and spectra (126 s) were acquired sequentially after its addition. The extracellular pH was maintained at 6.5 during the experiment by automatic addition of NaOH.

Table 1. Composition of CDM used for growth of *S. pneumoniae* in pH-controlled batch cultures

**Components**	**Concentration (g l^{-1})**	**Components**	**Concentration (g l^{-1})**
**Buffers/Salts**		**Aminoacids (10^{-3})**	
KH_2PO_4	3.0	Alanine	0.24
K_2HPO_4[a]	2.5	Arginine	0.13
Na - acetate	1.0	Asparagine	0.35
$(NH_4)_3$ - citrate	0.6	Aspartate	0.40
Na- pyruvate	0.1	Cysteine-HCl	0.40
**Vitamins (10^{-3})**		Glutamate	0.50
Choline-HCl	0.01	Glutamine	0.39
Na-p-Aminobenzoate	5.0	Glycine	0.18
D-biotin	2.5	Histidine	0.15
Folic acid	1.0	Isoleucine	0.21
Nicotinic acid	1.0	Leucine	0.46
Ca (D^+) Pantothenate	1.0	Lysine	0.44
Pyridoxamine-HCL	2.5	Methionine	0.13
Pyridoxine-HCl	2.0	Phenylalanine	0.28
Riboflavin	1.0	Proline	0.68
Thiamine-HCl	1.0	Serine	0.34
DL-6,8-Thioctic acid	1.5	Threonine	0.22
Vitamin B_{12}	1.0	Tryptophane	0.05
**Nucleobases (10^{-2})**		Valine	0.33
Adenine	1.0	**Micronutrients (10^{-1})**	
Uracil	1.0	$MgCl_2$	2.0
Guanine	1.0	$CaCl_2$	0.4
Xanthine	1.0	$ZnSO_4$	0.05

[a]K_2HPO_4 is replaced by disodium β-glycerophosphate (21 g l^{-1}) for growth without pH control.

Table 2. Growth parameters (length of lag, maximal growth rate and biomass) obtained for strain R6 cultures started with precultures harvested in different phases of growth, as in Fig. 2. The values for maximal growth rate and maximal biomass are averages of at least two independent experiments and the errors are reported as ± SD.

**Preculture**			**Culture (as in Fig. 2)**		
**Growth phase**	**Time (h)**	**OD_{600}**	**Lag**	**μ_{max} (h^{-1})**	**OD_{Max}**
LExp	6-7	0.8-1.0	No	0.78 ± 0.03	3.7 ± 0.0
EStat	8-9	1.4-1.6	No	0.37 ± 0.00	2.0 ± 0.3
LStat	18	~1.0	No growth[a]	-	-

LExp, late-exponential, EStat, early-stationary, LStat, late-stationary phases of growth; [a]No growth observed during the 14 h of monitoring.

Table 3. Growth parameters (length of lag, maximal growth rate and biomass) obtained for strains R6 and D39 cultured in CDM containing 61 ± 1 mM glucose, with pH-controlled at 6.5, at 37ºC and semi-aerobic (initial air tension 50-60%), anaerobic (argon atmosphere) or aerobic (constant air tension 40%) conditions, as in Fig. 3. The values for maximal growth rate and maximal biomass are averages of at least two independent experiments and the errors are reported as ± SD.

	**Semi-aerobic**		**Anaerobic**		**Aerobic**	
	**D39**	**R6**	**D39**	**R6**	**D39**	**R6**
**Lag**	No	No	No	No	No	No
**μ_{max} (h^{-1})**	0.80 ± 0.00	0.69 ± 0.03	0.55 ± 0.04	0.78 ± 0.03	1.00 ± 0.05	1.07 ± 0.04
**OD_{Max}**	1.3 ± 0.1	3.6 ± 0.2	1.4 ± 0.2	3.7 ± 0.0	0.49 ± 0.03	0.23 ± 0.02

Table 4. Product yields, substrate consumption rate, total substrate consumed, carbon and redox balances and growth and energetic parameters as determined from substrate and fermentation product analysis at the time-point of maximal biomass achieved by D39 and R6 strains cultured in CDM containing 61 ± 1 mM glucose, with pH-controlled at 6.5, at 37ºC, under semi-aerobic (initial air tension 50-60%) and anaerobic (argon atmosphere) conditions. Values of at least two independent experiments were averaged and errors are reported as ± SD.

	**Semi-aerobic**		**Anaerobic**	
	**D39**	**R6**	**D39**	**R6**
**Product yields**[a]				
Lactate	1.63 ± 0.01	1.81 ± 0.10	1.77 ± 0.01	1.86 ± 0.04
Pyruvate[b]	0.04 ± 0.01		0.01 ± 0.00	
Formate	0.06 ± 0.01	0.06 ± 0.01	0.07 ± 0.01	0.06 ± 0.01
Acetate	BDL	0.04 ± 0.02	BDL	0.05 ± 0.00
Ethanol	BDL	0.02 ± 0.01	BDL	0.02 ± 0.00
H_2O_2 (µM)	10 ± 0	ND	ND	ND
**q_s^{max} (µmol min^{-1} mg^{-1} prot)[c]**	0.92 ± 0.04	0.76 ± 0.16	0.60 ± 0.16	0.94 ± 0.05
**Consumed substrate (%)**	28 ± 0	100 ± 0	34 ± 5	98 ± 1
**Carbon balance**[d]	87 ± 1	93 ± 5	93 ± 1	96 ± 2
**Redox balance**	85 ± 1	93 ± 6	89 ± 1	94 ± 2
**Biomass yield (g mol^{-1} Glc)**	26.2 ± 1.0	23.1 ± 1.2	24.5 ± 0.1	24.6 ± 1.9
**ATP yield (mol mol^{-1} Glc)**	1.7 ± 0.0	1.9 ± 0.1	1.8 ± 0.0	2.0 ± 0.0
**Y_{ATP} (g biomass mol^{-1} ATP)**	15.0 ± 0.7	19.3 ± 0.6	13.8 ± 0.0	12.6 ± 0.8

[a]Product yields, [End-product] / [Glucose consumed]; [b]Blank cells, negative yields were found for these conditions (cells used pyruvate from the medium); [c]q_s^{max} was estimated from a first-order derivative of a polynomial fit of the measured substrate consumption time series. [d]Carbon balance is the percentage of carbon in metabolized glucose that is recovered in the fermentation products (lactate, formate, acetate and ethanol) and pyruvate. Dry weight (DW) was used as a measure of cell mass. BDL, below detection limit; ND, not determined.

Table 5. Enzyme specific activities determined in late-exponential fresh cell lysates or cell-free extracts of the D39 and R6 strains grown in CDM containing 60 mM glucose under semi-aerobic (rubber-stoppered bottles) or aerobic conditions (constant air tension 40%).

	**Semi-aerobic**		**Aerobic**	
**Enzyme**[a]	**D39**	**R6**	**D39**	**R6**
**NADH oxidase**	1.34 ± 0.00	1.02 ± 0.05	0.99 ± 0.13	0.42 ± 0.01
**Pyruvate oxidase**	ND	ND	0.04 ± 0.00	0.15 ± 0.00
**Lactate dehydrogenase**	4.75 ± 0.24	3.98 ± 0.40	ND	ND

[a]Enzyme activities are expressed in micromoles per minute per milligram of protein and are means of at least two independent experiments. Errors are reported as ± SD. ND, not determined.

Table 6. Product yields, total substrate consumed, carbon and redox balances and growth and energetic parameters as determined from substrate and fermentation product analysis, at the time-points 2h (transition phase) and 3h (stationary), in D39 and R6 strains cultured in CDM containing 61 ± 1 mM glucose, with pH-controlled at 6.5, at 37ºC, under aerobic conditions (constant air tension of 40%). Values of at least two independent experiments were averaged and errors are reported as ± SD.

	**Aerobic**			
	**Transition phase**		**Stationary**	
	**D39**	**R6**	**D39**	**R6**
**Product yields**[a]				
Lactate	1.06 ± 0.07	0.67 ± 0.09	0.47 ± 0.09	0.34 ± 0.02
Pyruvate[b]				
Formate	BDL	BDL	BDL	BDL
Acetate	0.92 ± 0.11	1.32 ± 0.08	1.53 ± 0.09	1.64 ± 0.01
Ethanol	BDL	BDL	BDL	BDL
H_2O_2	0.37 ± 0.01	0.60 ± 0.01	0.82 ± 0.01	0.91 ± 0.01
**Consumed substrate (%)**	3.7 ± 0.5	2.8 ± 0.2	4.4 ± 0.1	2.8 ± 0.4
**Carbon balance**[c]	100 ± 0	100 ± 0	100 ± 0	99 ± 1
**Redox balance**	ND	ND	ND	ND
**Biomass yield (g mol^{-1} Glc)**	64.6 ± 0.4	52.6 ± 4.0	68.1 ± 2.4	50.9 ± 6.6
**ATP yield (mol mol^{-1} Glc)**	2.9 ± 0.2	3.3 ± 0.1	3.5 ± 0.1	3.6 ± 0.0
**Y_{ATP} (g biomass mol^{-1} ATP)**	22.2 ± 1.2	15.8 ± 1.6	19.3 ± 1.1	14.1 ± 1.8

[a]Product yields, [End-product] / [Glucose consumed]; [b]Blank cells, negative yields were found for these conditions (cells used pyruvate from the medium); [c]Carbon balance is the percentage of carbon in metabolized glucose that is recovered in the fermentation products (lactate and acetate). Dry weight (DW) was used as a measure of cell mass. BDL, below detection limit; ND, not determined.

Table 7. Growth parameters (length of lag, maximal growth rate and biomass) obtained for strains R6 and D39 cultured in CDM containing 0.5, 1 or 3% (wt/vol) glucose (Glc), with pH-controlled at 6.5, at 37ºC, and under semi-aerobic conditions (initial air tension 50-60%). The values for maximal growth rate and maximal biomass are averages of at least two independent experiments and the errors are reported as ± SD.

	**0.5% Glc**		**1% Glc**		**3% Glc**	
	**D39**	**R6**	**D39**	**R6**	**D39**	**R6**
**Lag**	No	No	No	No	No	Yes (1h)
**μ_{max} (h^{-1})**	0.80 ± 0.06	0.68 ± 0.01	0.80 ± 0.00	0.69 ± 0.03	0.70 ± 0.01	0.47 ± 0.01
**OD_{Max}**	1.5 ± 0.0	2.2 ± 0.2	1.3 ± 0.1	3.6 ± 0.2	1.4 ± 0.0	3.1 ± 0.1

Table 8. Product yields, total substrate consumed, carbon and redox balances and growth and energetic parameters as determined from substrate and fermentation product analysis, at the time-point of maximal biomass achieved by D39 and R6 strains cultured in CDM containing 61 ± 1 mM glucose and supplemented with 30 mg l^{-1} uracil, with pH-controlled at 6.5, at 37ºC, under semi-aerobic conditions (initial air tension 50-60%). Values of at least two independent experiments were averaged and errors are reported as ± SD.

	**Semi-aerobic 30 mg l^{-1} uracil**	
	**D39**	**R6**
**Product yields**[a]		
Lactate	1.62 ± 0.04	1.82 ± 0.01
Pyruvate[b]	0.01 ± 0.00	
Formate	0.20 ± 0.02	0.05 ± 0.00
Acetate	0.10 ± 0.02	0.04 ± 0.00
Ethanol	0.09 ± 0.03	0.01 ± 0.00
**Consumed substrate (%)**	55 ± 0.4	100 ± 0
**Carbon balance**[c]	91 ± 1	93 ± 1
**Redox balance**	90 ± 1	92 ± 1
**Biomass yield (g mol^{-1} Glc)**	34.7 ± 0.1	22.9 ± 0.2
**ATP yield (mol mol^{-1} Glc)**	1.8 ± 0.1	1.9 ± 0.1
**Y_{ATP} (g biomass mol^{-1} ATP)**	19.3 ± 0.6	12.1 ± 0.2

[a]Product yields, [End-product] / [Glucose consumed]; [b]Blank cells, negative yields were found for these conditions (cells used pyruvate from the medium); [c]Carbon balance is the percentage of carbon in metabolized glucose that is recovered in the fermentation products (lactate, formate, acetate and ethanol) and pyruvate; Dry weight (DW) was used as a measure of cell mass.

Supporting Information

Figure S1. Kinetics of oxygen consumption of strains D39 and R6 grown under semi-aerobic conditions. Strains D39 (□) and R6 (■) were grown under semi-aerobic conditions (initial specific air tension of 50-60%) as in Fig. 3A. The oxygen consumption rates (q_s^{max}) are also shown. The plotted curves are averages of two independent experiments ± SD.

Figure S2. Effect on growth of increasing a single nucleobase. Growth profile of strain D39 in CDM containing 0.25% (wt/vol) glucose with 30 mg l^{-1} of the specified nucleobase. Cultures were prepared in 250 µl in 96-well microtiter plates and growth monitored at 595 nm and 37ºC. Symbols: (◇),G, A, X, U 10 mg l^{-1} each; (□), G, A, X 10 mg l^{-1} each plus 30 mg l^{-1} U; (△), G, A, U 10 mg l^{-1} each plus 30 mg l^{-1} X; (○), G, X, U 10 mg l^{-1} each plus 30 mg l^{-1} A; (- - -), A, X, U 10 mg l^{-1} each plus 30 mg l^{-1} G. G= guanine; A= Adenine; X= Xanthine; U= Uracil.

Figure S3. Pneumococcal lysis in resting cell suspensions. Optical density variation during glucose (20 mM) metabolism of resting cells of strain R6, grown as for *in vivo* NMR, suspended in 50 mM KP_i with (■) 2% or (●) 0% (wt/vol) choline. OD_{600} was measured with intervals of 15 minutes.
